# Conserved and variable correlated mutations in the plant MADS protein network

**DOI:** 10.1186/1471-2164-11-607

**Published:** 2010-10-28

**Authors:** Aalt DJ van Dijk, Roeland CHJ van Ham

**Affiliations:** 1 Applied Bioinformatics, PRI, Wageningen UR, Droevendaalsesteeg 1, 6708 PB Wageningen, The Netherlands

## Abstract

**Background:**

Plant MADS domain proteins are involved in a variety of developmental processes for which their ability to form various interactions is a key requisite. However, not much is known about the structure of these proteins or their complexes, whereas such knowledge would be valuable for a better understanding of their function. Here, we analyze those proteins and the complexes they form using a correlated mutation approach in combination with available structural, bioinformatics and experimental data.

**Results:**

Correlated mutations are affected by several types of noise, which is difficult to disentangle from the real signal. In our analysis of the MADS domain proteins, we apply for the first time a correlated mutation analysis to a family of interacting proteins. This provides a unique way to investigate the amount of signal that is present in correlated mutations because it allows direct comparison of mutations in various family members and assessing their conservation. We show that correlated mutations in general are conserved within the various family members, and if not, the variability at the respective positions is less in the proteins in which the correlated mutation does not occur. Also, intermolecular correlated mutation signals for interacting pairs of proteins display clear overlap with other bioinformatics data, which is not the case for non-interacting protein pairs, an observation which validates the intermolecular correlated mutations. Having validated the correlated mutation results, we apply them to infer the structural organization of the MADS domain proteins.

**Conclusion:**

Our analysis enables understanding of the structural organization of the MADS domain proteins, including support for predicted helices based on correlated mutation patterns, and evidence for a specific interaction site in those proteins.

## Background

New mutations continually arise and are the source of genetic diversity. They provide the material on which selection acts; in large, sexual populations, beneficial mutations will reach fixation, and most deleterious mutations will be lost. However, in the case of deleterious mutations, a compensatory mutation may occur that renders the two mutations neutral or beneficial as a pair and causes them to be preserved by selection. In protein-coding sequences, coevolution of residues can occur as compensation of changes in e.g. volume or charge, or because of the simultaneous involvement of residues in e.g. ligand binding. This implies that residues which show such correlated mutations are expected to be located close to each other in the 3 D structure of a protein. An early observation of this kind was obtained in a set of virus sequences, where the positions in the sequence that showed an identical pattern of variation were in most cases close together in the 3 D structure [1]. Several studies have reported similar observations and have made use of such information e.g. to engineer artificial domains [2], to predict interhelical contacts in transmembrane proteins [3], to analyze functional dependencies observed within HIV genes [4], to predict functionally important residues [5] or to distinguish between correct and incorrect models for the 3 D structure of proteins [6].

A number of methods have been developed to search for correlated mutations, and their results are mostly validated by comparing with distances between residues in crystal structures. A distinction can be made between pairwise correlation methods (which might be based on substitution matrix scores or related physicochemical characteristics) [7,8] and information-theory based methods [9-11]. The former seem to outperform the latter when using enrichment of residue pairs at short distances as a criterion [12,13]. Although several correlated mutation measurements yield reasonable accuracy for intramolecular contact map prediction, the accuracy level drops in intermolecular contact prediction [14].

On a higher level, similarity between phylogenetic trees is related to protein interactions in large sets of interacting families [15-22]. However, it has been heavily debated whether this signal is due to true coevolution, i.e. compensatory mutations between residues in the binding partners [23]. A number of factors affecting sets of proteins, such as similar expression patterns or functioning in a given biochemical pathway, can generate similarity in evolutionary rates [24]. Families with similar evolutionary rates in different organisms will present similar trees, without the need for co-adaptation between the corresponding proteins. Although this confounding effect takes place at the level of phylogeny, residue-level correlated mutations also contain noise caused by evolutionary processes related to common ancestry, such as changes in codon usage or amino acid frequencies [25,26]. Hence, misleading signal can be caused by phylogenetic correlations between homologous sequences and from correlation due to factors other than spatial proximity. This highlights the need to distinguish between observed "covariation", and true "coevolution", which is what we would like to infer based on those observed signals which do however contain noise.

Plant MADS domain transcription factors (TFs) are involved in regulation of a variety of developmental processes such as floral transition and flower development [27,28]. They "do it together" [29] in the sense that they are engaged in protein interactions and form protein complexes that are required for binding DNA. An analysis of the interaction capacity of all members of the family in *Arabidopsis *revealed the ability to form 110 different dimers [30] among 27 members of the subfamily of MIKC-type (or type II) MADS domain proteins. These TFs have in addition to the MADS (M) domain an I, K and C-domain [31,32].

A couple of structures are available for dimers of MADS domains (followed by a domain with some homology to the I domain) [33-38], but structural information for the other domains is lacking. The structures show that two MADS domains extensively contact each other, but mutagenesis data indicate that also other parts of the MIKC proteins contact each other. In particular, the I-domain is involved in determining interaction specificity [39,40] and the K-domain is important for dimerization [41-45]. A few computational studies previously analyzed plant MADS domain protein sequences in order to find functionally important regions, albeit without explicit reference to their role in interaction specificity [46-48]. Other computational studies focused on the evolution of the interaction network via duplications [49] or on simulating models for gene- and/or protein-interactions [50-52]. Recently, we developed a method aimed at predicting interaction sites using experimental interaction data and applied it to the MADS domain protein family [53] followed by experimental testing of sites governing interaction specificity [54].

Here, we present a novel approach to analyzing correlated mutations and testing their validity. We analyze correlated mutations in a family of interacting proteins. This provides a convenient way to compare correlated mutations between those proteins and assess whether correlated mutations are 'conserved' between them. Secondly, it allows comparison of correlated mutations observed between pairs of interacting proteins with those observed between pairs of non-interacting proteins, where the latter provide a unique background-model for assessment of significance of the observed intermolecular correlated mutations. Hence, our results contribute to the interpretation of correlated evolution signals.

We integrate our results with available structural, bioinformatics and experimental data for the plant MADS domain proteins and in this way we obtain clues about the structural organization of these proteins and their complexes.

## Results

We will first discuss sequence retrieval, followed by correlated mutation analysis and validation of the results using various types of independent data. Next, conservation of correlated mutations between homologous positions in various proteins will be analyzed, which provides a novel way to assess the amount of information correlated mutations contain. Finally, our results will be applied in prediction of protein interactions and scrutinized to obtain structural insight into the MADS proteins.

### Sequence data

**Figure 1:**
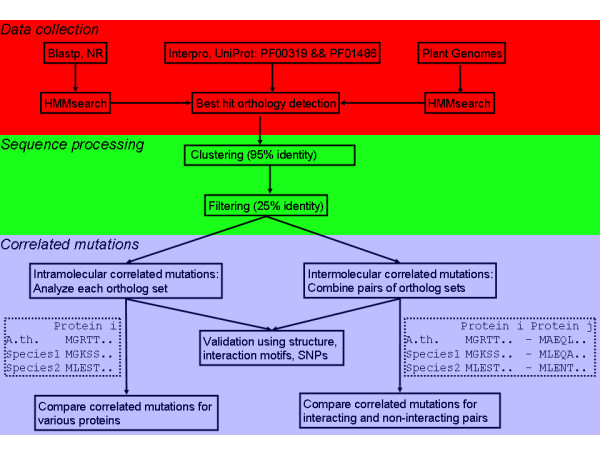
**Correlated mutation analysis of MADS domain proteins: workflow diagram**. Sequences were obtained via blast, interpro and from a set of sequenced plant genomes. Orthologs were assigned using a best-hit criterium, followed by clustering to group sequences within each species with a very high sequence identity and filtering to remove sequences with low sequence identity to Arabidopsis MADS sequences. Intramolecular and intermolecular correlated mutations were obtained, and validated using various datasets. Subsequently, conservation of correlated mutations between proteins was analyzed, and correlated mutations were compared between interacting and non-interacting proteins. *A. th*. is *Arabidopsis thaliana*.

The workflow followed to obtain sequences and perform further analysis is illustrated in Figure 1. In total, 1760 sequences were obtained using Interpro, 2043 using blast and 303 from sequenced plant genomes; after clustering and filtering with a minimum of 25% sequence identity to one of the Arabidopsis MADS proteins, 2080 sequences were retained. As explained in the Methods section, we do not strictly define 1-to-1 orthology, but for the sake of clarity we will refer in the sequel to these sequences as (putative) orthologs. For the following 12 MADS proteins there were at least 30 sequences of putative orthologs available: AG (114 sequences), AGL6 (56), AP1 (127), AP3 (339), FUL (117), PI (235), SEP1 (95), SEP3 (107), SHP1 (77), SOC1 (65), STK (42) and SVP (34). These proteins were analyzed for intramolecular correlated mutations (sequence identifiers are listed in Additional File 1).

Combining the sequences with existing interaction data [30] allowed in total 34 different pairs of interacting Arabidopsis proteins to be analyzed, with a minimum of 30 ortholog pair sequences (Additional File 2). As background model, 34 pairs of non-interacting MADS pairs were used for which a minimum of 30 ortholog pairs were available. Because of the way we deal with co-orthologs (see Methods), there are cases of MADS domain proteins that pass the threshold of 30 sequences only in the intermolecular analysis and not in the intramolecular analysis.

### Validation of correlated mutation analysis

Correlated mutations were obtained for intra- and inter-molecular sequence alignments using CAPS (see Methods). Additional Files 3 and 4 contain lists of these results. To validate the observed correlated mutation pairs, we compared them with available structural data (a crystal structure is available for the human MADS domain), previously predicted interaction motifs and Single Nucleotide Polymorphisms (SNPs).

### Validation: structure data

**Figure 2:**
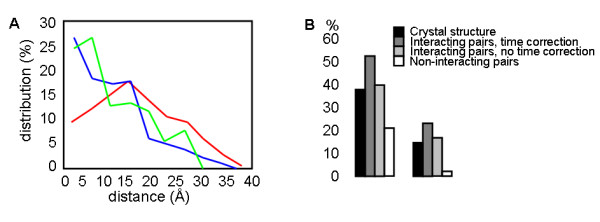
**Short distance enrichment of correlated mutations in MADS domain**. **(A) **Distribution of distances in intramolecular correlated mutation residue pairs. All residue pairs in crystal structure (red), correlated mutation analysis without time correction (blue) and correlated mutation analysis with time correction (green); **(B) **Percentage of pairs within 15 Å (left panel) and within 10 Å (right panel) for all intermolecular residue pairs in the crystal structure (black), residue pairs resulting from correlated mutation analysis of interacting protein pairs using time correction (dark grey), residue pairs from correlated mutation analysis of interacting pairs without using time correction (light grey) and residue pairs from correlated mutation analysis of non-interacting protein pairs (white).

Residues which show correlated mutations are expected to be located close to each other in tertiary and quaternary structure. Comparison of the distribution of distances for intramolecular correlated mutation residue pairs in the crystal structure of the MADS domain shows that there is indeed a clear enrichment at low distances, compared to all residue pairs in the crystal structure (Figure 2A). The enrichment for the analysis with time correction (see Methods) is similar to the enrichment for the analysis without time correction; for the former, 74% of the correlated mutation residue pairs are within 15Å, whereas for the latter this is 77%. These numbers should be compared with the percentage of all residue pairs within 15Å in the crystal structure, which is 51%. Comparison with randomly selected subsets of residue pairs in the crystal structure indicates that this enrichment is statistically significant (p < 0.001).

For the intermolecular correlated mutation analysis, the analysis of interacting protein pairs using time correction (see Methods) shows an enrichment in residues within 15Å, compared to all residue pairs (Figure 2B). Such enrichment is not found for interacting protein pairs analyzed without time correction, nor for non-interacting pairs analyzed either with or without time correction (Figure 2B). Hence, these two background models strongly support the significance of the distance enrichment for the resulting residue pairs in the correlated mutation analysis of the interacting MADS domain proteins. Note that the correlated mutation analysis of non-interacting pairs results in a strikingly lower percentage of pairs of residues with small distance (Figure 2), an observation for which we miss a clear interpretation.

The enrichment of residues which are in contact (within 15Å) across the interface is reasonably strong (55% of the correlated mutation residue pairs are in contact vs. 39% for all residue pairs), but less so than what is seen for the intramolecular correlated mutation analysis. This is in line with what has been observed previously for intermolecular correlated mutation analysis (see introduction). One reason could be that the correlated mutation analysis will inherently focus on residues which are not conserved (because otherwise there will be no coevolution effect). For a large part, residues at the interface will be conserved, meaning that a lot of residue-pairs will not show up in the correlated mutation analysis. Another factor obviously is the assumption (inherent to intermolecular correlated mutation analysis) that orthologs will have similar interaction partners, a hypothesis for which evidence exists [55] but that also has been challenged [56]. The clear difference between the interacting and non-interacting protein pairs does however strongly argue for the importance of the correlated residue pairs that we recover. The results presented here are for using a cutoff for the correlation coefficient of 0.4, but qualitatively they are similar for higher cutoffs (only the number of reported pairs is lower). Because enrichment of residue pairs at small distances was only observed for the analysis with time correction, in the sequel we use results from that analysis only. To further analyze the significance of the observed short distance enrichment for the intermolecular correlated mutations, a resampling analysis was performed. This is described in detail in Additional File 5; it clearly confirmed the significance of our results.

### Validation: comparison with predicted interaction motifs

For the intermolecular correlated mutation results, a comparison was made with motif pairs which were previously predicted to determine MADS interaction specificity [53,54]. The rationale behind this comparison is that both motifs and correlated mutations should contain information about interaction residues. Overall, there are large differences between different interacting protein pairs with respect to the number of correlated mutation positions and motifs that coincide. The lowest coincidence was found for the AGL12-AGL16 interaction for which only 10% of the residues involved in correlated mutation were overlapped by predicted interaction motifs. In contrast, three interacting protein pairs (ANR1-SOC1, AGL21-FUL, and SOC1-SVP) showed over 70% of their correlated mutation positions overlapped by predicted interaction motifs. However, there was a clear difference between the results for the interacting pairs and non-interacting pairs. For the interacting pairs, 55% of the motif positions was overlapped by at least one correlated mutation position, and 39% of the correlated mutation positions was covered by a motif, whereas for the non-interacting pairs, 42% of the motif positions was overlapped by at least one correlated mutation position, and 32% of the correlated mutation positions was covered by a motif. Comparison with randomly generated position pairs (see Methods) showed that the F-score (harmonic mean of coverage of correlated mutation positions and of predicted interaction motifs, 0.46 for the interacting pairs and 0.37 for the non-interacting pairs) was significantly different from random for the interacting protein pairs (p < 0.001), but not for the non-interacting protein pairs (p~0.5).

**Figure 3:**
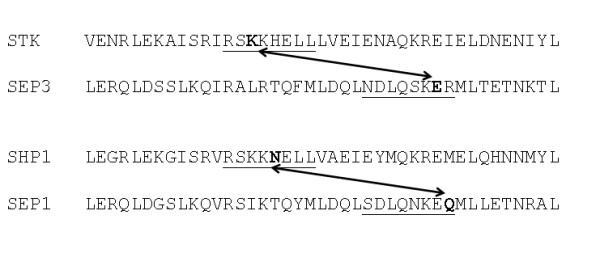
**Example of consistent overlap between motif pair and correlated mutation pair**. Sequence alignment of part of the K-domain region is shown. In the interacting pairs STK-SEP3 and SHP1-SEP1, there is a correlated mutation pair (bold residues) which overlaps with the same motif pair (underlined).

Both the previously predicted motif pairs and the correlated mutation position pairs predict connections between regions in pairs of interacting sequences. In the comparison above, this was not taken into account, but we would expect that overlap between motif pairs and correlated mutation pairs would be 'consistent' in the sense that the two motifs that constitute a motif pair each overlap with one of the two positions of a given correlated mutation pair. Analysis of the number of ortholog pairs in which a given motif pair had such consistent overlap with correlated mutation positions indicates that this is higher for the interacting pairs than for the non-interacting pairs (data not shown). There is one motif pair which overlaps consistently with a correlated mutation pair in four different interacting protein pairs, and one which overlaps consistently in three different interacting pairs. The former connects two parts of the K-domain (Figure 3), whereas the latter connects two parts of the I-domain. The various correlated mutations which show consistent overlap with motif pairs are shown in Additional File 6. These positions are strong candidates for further investigation as important residues for protein-protein interactions of the MADS domain proteins.

### Validation: comparison with SNP data

Finally, we compared the intermolecular correlated mutation positions with available Arabidopsis SNP data [57]. For the interacting pairs, we found 207 non-synonymous SNPs without overlap with a correlated mutation position, and 19 with overlap with a correlated mutation position. For the non-interacting pairs, these values are 581 and 74, respectively. This means that the fraction of non-synonymous SNPs covering a correlated mutation site is smaller for the interacting pairs (8.4%) than for the non-interacting pairs (11.3%). Of course at longer evolutionary distances one would expect a correlated mutation position to be variable (otherwise it would not be detected as a correlated mutation position), but if these sites are functional (i.e. in our context, important for the interaction) then at short evolutionary distances it is reasonable to expect that they are conserved, and the fact that they are more conserved for the interacting compared to the non-interacting protein pairs is additional validation of our results. These results are reinforced by the fact that for the synonymous SNPs, no such difference between interacting and non-interacting pairs is observed (both display an overlap of ~10% between synonymous SNPs and correlated mutations).

### Validation: general trends

Overall, the comparison of correlated mutation positions with structural data, interaction motifs and SNPs show the same trend: correlated mutations from interacting pairs have enrichment in signals compared to non-interacting pairs. In addition, the intramolecular correlated mutations show clear distance enrichment. Hence, all observed trends, although sometimes weak, are consistent and point towards biological significance of the observed signals.

### Conserved correlated mutations

An intriguing question is whether positions with correlated mutations in various protein subfamily members are conserved for being correlated or not, because this would give further insight into the mechanism behind correlated mutations. Note that the use of the term "conservation" here is somewhat different from its most common use to describe sequence conservation, but was chosen because it best describes the phenomenon of observing a feature (correlated mutation in this case) in multiple instances of a sequence alignment (such use is not unprecedented, compare for example with the use of "structure conservation"). To answer this question for the MADS proteins, we investigated for all intramolecular correlated mutation pairs in a given protein whether they were detected in other MADS proteins as well, in which case they were called "conserved" in these other proteins. We first analyzed whether there is more conservation of correlated mutations for pairs of proteins with higher sequence identity, but this was not the case. Overall, 63% of the correlated mutation pairs are conserved in at least one other MADS protein, and 37% are not (conserved intramolecular correlated mutations are listed in Additional File 7). For the non-conserved cases, there are two possibilities: either a correlated mutation is not conserved because the residues themselves at these positions are conserved, i.e. not varying, in other MADS domain proteins (which would support their functional importance) or there is variation at the positions in other MADS domain proteins but it is not correlated. To distinguish between these two possibilities, sequence entropy was calculated for each column in the multiple sequence alignments (see Methods). Next, homologous positions in various MADS domain protein alignments were divided into two groups, one with correlated mutation occurring at that position, and one without. Sequence entropy was compared between those groups. This showed that correlated mutation positions which were conserved in at least one other protein had on average a higher sequence entropy (2.2 +/- 0.5) than the homologous positions where the correlated mutations were not conserved (1.9 +/- 0.2). Indeed, in 74% of the cases conserved correlated mutation positions had a higher entropy than the homologous positions where no correlated mutation was detected. This means that no correlated mutation was observed in those homologous positions because they were less variable. Correlated mutation positions that were not conserved in any other protein did not show such difference in sequence entropy. Hence, for correlated mutations that are not conserved at all, the homologous positions in other proteins are as variable as the position where the correlated mutation occurs, but in these other proteins no compensatory correlated mutation occurs. These results fit within the framework of correlated mutations occurring when a second mutation compensates for an earlier deleterious one and indicate that this is most likely the case for correlated mutations which are conserved in at least one other protein. For those correlated mutations that are not conserved at all this interpretation is less likely because these positions show as much variation in other proteins as in the protein where the correlated mutation occurs.

**Figure 4:**
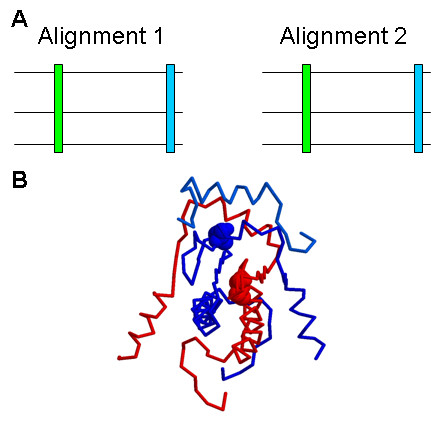
**Conserved intermolecular correlated mutation**. **(A) **Illustration of conserved correlated mutation concept: a correlated mutation is observed at homologous positions in two sequence alignments for two different MADS-domain protein subfamilies (or for two different pairs of interacting MADS domain protein subfamilies). **(B) **Two chains of MADS domain protein dimer (structure with PDB id 1n6j) are shown in blue and red. Spacefill indicates residues 47 and 66, which are located close to each other and which are detected as a correlated mutation pair both in the interacting pair AGL6-SOC1 and SEP1-SOC1.

For the intermolecular correlated mutations, several correlated residue pairs are found in the MADS domain for two interacting pairs of Arabidopsis MADS proteins which can be compared with structure data as presented above. These conserved correlated mutation pairs show similar enrichment for short distances as observed for all correlated mutation pairs: 11 out of 20 (55%) of these pairs are located in each others neigbourhood in the 3 D structure (within 15Å; see Additional File 7). One example is the residue pair 47 - 66, which is found as a correlated residue pair in AGL6 - SOC1 and SEP 1 - SOC1. The residues involved are located within a predicted distance of 8.8Å (Figure 4). Among the correlated mutation pairs resulting from the non-interacting MADS pairs, only one pair is found in two different protein pairs; these two residues are not located in each others neighbourhood. For the domains outside the MADS domain, comparison with protein structure data is not possible; however, again the resulting correlated mutation positions are more conserved among the interacting pairs than among the non-interacting pairs. There are in total 64 conserved groups of correlated mutation positions in the interacting protein pairs (i.e. correlated mutation positions that are observed in more than one pair of interacting proteins), whereas there are only 49 such groups in the non-interacting protein pairs. Compared tot the total number of correlated mutation pairs, for the interacting proteins this is ~0.9% and for the non-interacting protein pairs this is ~0.4%. In addition, each group for the non-interacting protein pairs consists of only two different protein pairs, whereas for the interacting protein pairs there are two larger groups. The conserved intermolecular correlated mutations are shown in Additional File 7.

### Analysis of MADS domain protein and complex structure

Based on the analyses described above we conclude that the correlated mutation analysis results clearly contain biological signal. We now describe application of these results in order to obtain insight into the structural organization of MADS domain proteins and their complexes. In particular, we focus on the K-domain, because structure information is already available for the MADS and I domain (see above), and the C-terminal domain is predicted to be unstructured.

### Intramolecular organization of K-domain helices

**Figure 5:**
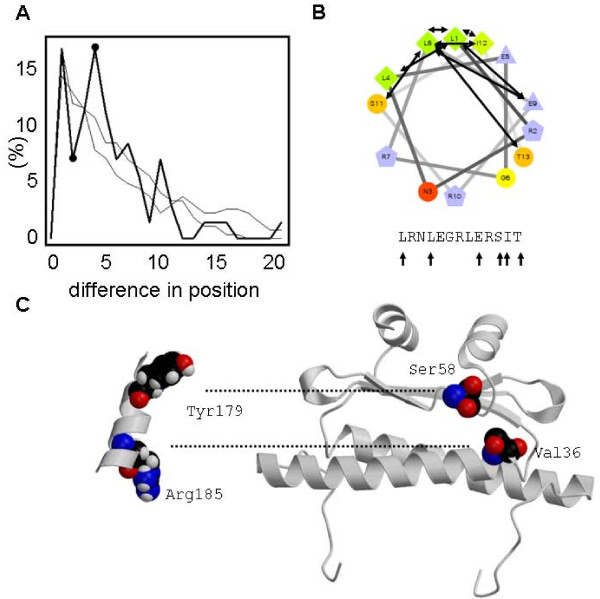
**Structural implications of intramolecular correlated mutations for MADS proteins**. **(A) **Comparison of correlated mutation pairs with predicted helices in the K-domain: histogram of differences in sequence position for observed correlated mutation positions within predicted coiled coil helices in the K-domain (thick line) and two representative examples of such distributions for positions within randomly selected sequence stretches. Note that these display the same decreasing trend (as expected) but the relevant feature is the absence of the difference between i+2 (low) and i+4 (peak), which for the real correlated mutation pairs is indicated by dots on top of the thick line. **(B) **Illustration of the observed preferred distance within helices for AG. Helical wheel illustrates coil, arrows indicate correlated mutation residue pairs. Sequence with arrows indicates correlated mutation residue positions. **(C) **Correlated mutation between residues from K-domain helix (left) and MADS domain (right) is conserved between SEP3 and AP1; based on this analysis, Tyr179 is predicted to contact Ser58, and Arg185 to contact Val36. Note that the distances between these residues within the K-domain and within the MADS domain are consistent with this hypothesis. Dotted lines indicate observed correlated mutations.

Although structure information is only available for the MADS and I domain, it is generally assumed that the K-domain consists of coiled coils. Our correlated mutation analysis can be used to validate this assumption. We predicted coiled coils in this domain (see Methods) and compared the correlated mutation positions with these predictions. The predicted helices are listed in Additional File 8. Based on the intra-helical organization of residues, one would expect a periodic pattern of connections between residues within predicted helices. This is indeed the case: on top of the expected decay with longer distance, there is a clear preference for residues *i-i+3*, *i-i*+5 and especially *i-i*+4 to be connected to each other, and for residues *i-i+2 *to be not connected. Note that, for example, the notation i-i+3 refers to pairs of residues which are separated in the amino acid sequence by two intervening residues. This pattern is not found when instead of the predicted helices, random stretches of equal length are defined and compared with the correlated mutation positions (Figure 5A, B); the difference in preference for *i-i+4 *vs *i-i+2 *is 9.8% (16.7% vs 6.9%, i.e. over two-fold enrichment for *i-i+4*) for the predicted helices, whereas only 10 out of 1000 randomizations (randomly selecting sequence stretches) have a similar or higher difference (p~0.01).

Next, we analyzed whether correlations were observed between helices, in order to infer their orientation with respect to each other. Because only a few intramolecular correlated mutation positions occur between predicted K-domain helices (15 pairs of positions, in 3 different proteins: AP1, SEP1 and SEP3; these predicted connections are listed in Additional File 9), our results suggest that these helices do not directly contact each other intramolecularly in most MADS domain proteins. This is in line with suggestions in the literature that these helices would be involved in intermolecular contact [43,44]. This suggestion is reinforced by the fact that we do observe intramolecular correlated mutations between the K-domain helices and the MADS/I domain: 115 pairs of positions in 8 different proteins (Additional File 10). These predicted connections mainly involve the first K-domain helix, which is indeed expected to contact the MADS/I domain as it is directly connected via the primary sequence. Of these pairs, only 10 are showing conservation, which is quite low compared to the overall conservation for correlated mutation pairs (63%, see above); however, one reason might be that the I domain is more variable and less well alignable than the MADS or K-domain. These cases of conserved correlated mutations are shown in Additional File 10. Two examples of such conserved predicted contacts are between Val36 resp. Ser58 and two residues in the first predicted K-domain helix of SEP3, and the same positions in AP1. An interesting aspect here is that Val36 and Ser58 are located close to each other (~9 Å) in a structure model of SEP3 based on the available crystal structure of the MADS domain, and the residues in the K-domain helix which show correlated mutation with these two residues have a sequential distance of 6 residues, corresponding with almost two turns of a helix, which corresponds to ~3 Å. Taking into account that contacts will be made via side chains, which bridge several Å, these distances show a nice match (Figure 5C).

### Analysis of intermolecular interactions

**Figure 6:**
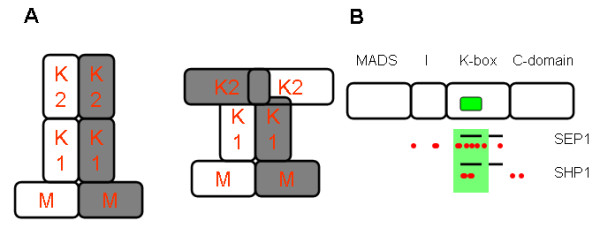
**Structural implications of intermolecular correlated mutations for MADS proteins**. **(A) **Intermolecular correlated mutations between K-domain helices do not support an "extended helix organization" (left panel), but suggest a more compact configuration (right panel). (**B) **Correlated mutation results support motif-based predicted interaction site. Predicted K-domain coiled coil regions (black lines), overlaid with predicted correlated mutation positions complementary to the 'hotspot region' in SOC1, for SEP1 and SHP1 (red circles). Most of the correlated mutation positions fall inside the first K-domain helix (green shade); based on motif predictions in combination with structural information, an interaction between the 'hotspot' region and the K-domain was hypothesized, for which the correlated mutation results provide additional support.

We also analyzed whether patterns of correlated mutations could provide insight into the intermolecular structural organization of the K-domain helices. One possible organization would be that the two or three K-domain helices from one protein form one extended "superhelix", which contacts an equivalent "superhelix" of the other protein. In this case, one would expect mainly intermolecular contacts between homologous helices. Some suggestive drawings have appeared in literature [58,59], but the correlated mutation analysis might give some more clear-cut insight here. The correlated mutation pairs that we observed indicated that out of all possible helix-helix contacts, there was not a specific preference for certain helix-helix contacts to be present (Additional File 11). This could indicate a more compact organization of those helices (Figure 6A). Note that the "superhelix" organization would also not be consistent with evidence that the last part of the K-domain is involved in formation of higher order complexes [43,60].

In a recent analysis of MADS interaction specificity [54] we in particular focused on one part of the I domain where we found a 'motif hotspot': experimental investigation with yeast-two-hybrid validated the importance of this region, and using available structural information we hypothesized that there would be an interaction between this region and a complementary region in the K-domain. As the motif in this region was specifically validated for the SOC1 protein, we analyzed correlated mutation pairs for SOC1 with interaction partners where the position in SOC1 overlapped with this 'hotspot' region. Several of the complementary correlated mutation pairs fall specifically in the first predicted helix in the K-region, providing additional validation for our original hypothesis (Figure 6B).

## Discussion

Our analysis of correlated mutations in the MADS domain protein family provides a unique way to investigate the amount of signal that such mutations leave in protein sequences. We studied correlated mutations in various family members in terms of their conservation, and were able to compare correlated mutations between interacting pairs of proteins and non-interacting pairs of proteins. The intramolecular correlated mutation results show a clear enrichment of residue pairs located close to each other in the MADS domain. There are some variations between proteins in the number of correlated mutation pairs and the percentage located close to each other. We did not observe a clear correlation between the number of sequences available for each protein and the number of correlated mutation pairs or the short distance enrichment. We also tested whether the number of predicted correlated mutation positions or the distance enrichment depended on quality measures of the alignments that were used (e.g. fraction of gaps in the alignment) but found no such correlation.

The majority of the intramolecular correlated mutations were observed in at least two MADS proteins, i.e. they showed conservation. We found that when such conserved correlated mutations were not observed in other MADS proteins, this is mostly because these positions are more conserved and not because of uncorrelated variability in these other proteins. This analysis gives additional support to the interpretation of correlated mutations as "one mutation followed by a compensatory mutation". Such support is important because of the need to infer "coevolution" based on observed "covariation", a process in which noise can be present, as discussed in the Introduction.

A possible confounding factor for intermolecular correlated mutation analysis is that we cannot be sure that the predicted orthologs in all the various species that we analyze do indeed interact. To get some further insight into this issue, we assembled a set of interacting MADS domain proteins from various species from literature [30,61-67]. Using sequence identity with Arabidopsis proteins as criterium, orthology relationships were predicted, and next we assessed whether the interaction would have been correctly predicted based on the Arabidopsis interaction data. This was the case in over 60% of the interactions (data not shown). A random prediction would have much lower success rate because there are much more non-interacting than interacting pairs of Arabidopsis MADS domain proteins. Still, this number clearly illustrates a problem with which all intermolecular correlated mutation approaches have to deal, i.e. that many interactions will be missed and/or incorrectly assigned. Indeed, validation by for example structure information shows that the fraction of residue pairs in close contact is lower for the intermolecular correlated mutations than for the intramolecular correlated mutations.

Our approach is unique in using a set of interacting protein pairs and a set of related non-interacting protein pairs as a reference. As the latter would be expected not to have correlations with each other, they serve as negative controls. Using these, we found i) that the overrepresentation of intermolecular residues at short distances is higher for interacting protein pairs than for non-interacting pairs; ii) that there is more consistency between results from different interacting pairs than between results from different non-interacting pairs; iii) that there is a better overlap between correlated mutation results from interacting protein pairs and our previously predicted interaction motifs than between correlated mutation results from non-interacting protein pairs and those motifs; and iv) that they have less overlap with SNPs. Although some trends are weak on their own, they are all consistent.

Our results here are complementary to our previous analysis of sequence determinants of MADS protein interaction specificity [53]. In particular, that analysis focused on using sequences from Arabidopsis MADS domain proteins in order to find motifs that are responsible for interaction specificity. In our current study, we use the large amount of sequence data that is available, in order to find correlated mutation pairs. There is no reason why these pairs should specifically contain information about interaction specificity, but rather one would expect that they contain information about interaction sites in general. As such, the predicted interaction motifs would be expected to form a subset of the correlated mutation sites, and in line with that, indeed the coverage of predicted interaction motifs by correlated mutation positions is higher than the coverage of correlated mutation positions by predicted interaction motifs. An important point is also that correlated mutation positions per definition are sites which are not conserved evolutionarily, whereas the motif positions are relatively conserved; this again limits the possible amount of overlap between these two analyses. Still, the fact that we do find significant overlap indicates that a combination of these two approaches might be particularly powerful.

## Conclusions

Our results provide understanding of structural properties of the important plant MADS proteins. In particular, our correlated mutation analysis confirms predicted helices in the K-domain, and supports a specific organization of these helices in the MADS dimers. Also, we obtain further support for an interaction region in the I domain. Hence, in addition to obtaining general insight into coevolution signals at the protein level, we also demonstrate the use of these signals to test specific hypothesis about structural properties of proteins.

## Methods

### Datasets

A set of type II MADS proteins was obtained as follows (Figure 1). Interpro [68] was used to obtain UniProtKB identifiers of sequences in various species that contained both a MADS domain and a K-domain (PFAM domains PF00319 and PF01486, respectively); these sequences were retrieved from UniProt [69]. Secondly, the NCBI web_blast.pl script was used with in turn each Arabidopsis type II sequence as query, searching the NR database with blastp. Hmmsearch [70] was used to select sequences with both a MADS domain and a K-domain. Thirdly, the genome sequences of rice [71], poplar [72], grape vine [73], Physcomitrella patens [74], maize http://www.maizesequence.org, medicago http://www.medicago.org/genome, papaya [75] and sorghum [76] were scanned using hmmsearch [70] to obtain sequences with both a MADS domain and a K-domain.

Next, orthologs were assigned to the various Arabidopsis sequences. We used a "best hit" criterion, based on the value of the sequence identity (calculated using gaps as non-indentical residues) after separately aligning each of the obtained sequences with each of the Arabidopsis sequences, using Muscle [77]. For the sequences obtained from the eight genomes (where we are relatively sure that all relevant sequences are obtained) this criterium was applied bi-directional, whereas for the other sequences it was only required that the respective Arabidopsis sequence was their best hit (and not that they were also the best hit of that Arabidopsis sequence). We tested however also the use of a bidirectional best-hit criterium for these other sequences, and found that it did not improve results. Note that a recent study suggested that it is beneficial to include both orthologs and paralogs in the multiple sequence alignment used as input for correlated mutation analysis [78]. Hence, a more restrictive bi-directional best hit approach would not necessarily be expected to give better results.

Subsequently, in each species separately, blastclust with sequence identity cutoff of 95% was used for each group of sequences which simultaneously were "best hits" for a given Arabidopsis sequence (the cutoff of 95% was based on the observation that this keeps the Arabidopsis MADS proteins apart). A representative for each cluster was chosen randomly, except that preference was given to Interpro-based sequences compared to blast-based sequences and sequences from the genomes were preferred over both Interpro-based sequences and blast-based sequences. In addition, at least 25% sequence identity between a sequence and the Arabidopsis sequence which was it best hit, was required.

Our choice to detect orthologs using blast hits is a pragmatic one. A more elaborate and time-consuming approach would be to make use of phylogenetic trees, which however have their own degree of uncertainty. We tested how different the results would be upon application of phylogenetic relationships from previously published phylogenetic trees for the MADS domain proteins AP1 and FUL [79]. When comparing with structure data, resulting correlated mutations for these cases did not contain more residue pairs at lower distances than what was obtained when using blast (data not shown). Hence we do not further discuss these results.

To analyze intramolecular correlated mutations, the only step to take next was to align the sequences of each Arabidopsis MADS domain protein with all its associated sequences, for which Muscle [77] was used. The alignments were used for the correlated mutation analysis if at least 30 sequences were present. For intermolecular correlated mutation analysis, interaction data from De Folter et al. were used [30]. We combined for each pair of interacting Arabidopsis sequences their predicted orthologs within each species. If in one species multiple sequences were best hits with one of the two interacting sequences we combined each of those with the best hits in that species of its interaction partner. For example, if Arabidopsis protein X and Arabidopsis protein Y interact and both have two best hits in a given species, in that species there are 2 * 2 = 4 combinations.

After alignment, the resulting sets of interaction pairs (each consisting of one original Arabidopsis interaction pair and the ortholog pairs obtained for various other species) were used as input for the correlated mutation analysis if at least 30 pairs were present. As a background model, non-interacting pairs with at least 30 associated sequence pairs were used as input.

Note that the cutoff on the number of sequences (30) that we use is somewhat arbitrarily but such cutoff is clearly needed because the smaller the number of sequences, the less reliable the correlated mutation results are.

### Correlated Mutation analysis with CAPS

CAPS [8] compares the correlated variance of the evolutionary rates at two sites in a multiple sequence alignment by comparing the transition probabilities between each pair of amino acids at the two sites, using the BLOSUM substitution matrix [80]. Because sequences that diverged longer ago are more likely to fix mutations at two sites by chance, BLOSUM values are normalized by the time of divergence between sequences using Poisson corrected amino acid distances; we performed analysis both with and without this time correction. The coevolution between two sites is then estimated as the correlation in the pairwise amino acid variability, relative to the mean variability per site. Correlated mutation pairs are grouped based on their connectivity to each other; only those "correlated groups" were analyzed.

To determine significance of these correlations, re-sampling can be performed. However, because this is computationally expensive (keeping in mind that we perform correlated mutation analysis for various MADS domain proteins and various pairs of MADS domain proteins), we chose to use a cutoff on the value of the correlation coefficient, which we set to 0.4, in agreement with previous correlated mutation analyses [3]. This is a conservative threshold as it is slightly above the lowest correlation coefficient values found to be significant in an earlier application of CAPS [81]. We performed resampling afterwards for a number of MADS protein pairs to analyze the significance of the results obtained when comparing the correlated mutations with available structural data (see below, randomization trials). We also tested a previously described approach to remove spurious phylogenetic correlation by using subalignments where specific clades are removed [8]. This approach was implemented by using small subunit ribosomal RNA sequences obtained from http://gobase.bcm.umontreal.ca/searches/gene.php to obtain distances between species and using Clustalw [82] to build a tree. As the results of this analysis did not improve compared to the results without this correction, we only present the latter results. This is in line with an analysis that showed that tree-aware correlated mutation methods did not outperform tree-ignorant methods [83].

### Comparison with protein structure data and predicted interaction motifs

Although no structure for plant MADS domain proteins is available, a couple of structures of human MADS domains are available. Of these, 1tqe, 1n6j, 1egw and 3kov are crystal structures of MEF2-type MADS domains, which are most related to plant MIKC (type II) MADS domains [84]. Because 1egw, the structure of human MEF2A [35] has the best resolution of these structures we chose this structure for comparison of the correlated mutation analysis results with structure data. The structure of human MEF2B, 1n6j [33], has the second-best resolution and we used this structure for comparison. Results of using this structure are almost indistinguishable from that of using 1egw, so we only report results for the latter.

Correlated mutation pairs were compared with protein structure data as follows. For all intra-and inter-molecular pairs of residues in the PDB structure 1egw, the shortest heavy-atom distance was obtained. Mapping of the Arabidopsis sequence to the structure was obtained via Muscle alignment. For this, residues 2-69 of the structure were used. For residues 2-59, which constitute the MADS domain, there is high overall sequence similarity with the plant MADS domain, and for residues 60-69 there is also reasonable sequence similarity with the first part of the plant I domain. For the various proteins, this similarity (amount of conservative substitutions) is at least 7 out of 10. However, the sequence identity with the plant I domain is lower than for the MADS domain, meaning that the results of comparison with this part of the structure could be more noisy.

In addition, correlated mutation pairs were compared with previously predicted interaction motifs [53]. Because these interaction motifs are grouped into pairs of complementary motifs, correlated mutation positions were compared both to individual motifs and to pairs of complementary motifs.

To predict coiled coils in the K-domain, a method which compares sequences with sequences of known coiled-coil proteins [85] was used, which is available via http://npsa-pbil.ibcp.fr/cgi-bin/npsa_automat.pl?page=npsa_lupas.html. Default settings were used (scoring matrix 2-MTIDK, no upweighting of positions a and d), and a window length of 14, minimum coil probability of 0.5 and minimum length of 4 residues was applied to predict coiled coil helices based on the predicted coil probabilities. Helical wheel representations were generated with http://rzlab.ucr.edu/scripts/wheel/wheel.cgi

Modelling of the structure of the MADS domain of SEP3 was performed using Modeller [86]. Out of 1000 generated models, the 10 best based on the objective score were used for docking. Modelling of a K-domain helix was performed in CNS [87]. Dihedral angle restraints were defined for backbone angles phi, -65° ± 20° and psi -40° ± 20°, respectively, and hydrogen bond restraints were defined between each O(*i*)-N(*i*+4) pair (lower and upper bound 2.3 and 3.5 Å, respectively) and O(*i*)-HN(*i*+4) pair (lower and upper bound 1.7 and 2.5 Å, respectively). The anneal.inp CNS-script was used, which applies a high-temperature torsion-angle dynamics phase followed by a torsion angle dynamics cooling phase and a second cartesian dynamics cooling phase. Ten structures were calculated, and the lowest energy structure was used. Protein structure figures were prepared using Molscript [88] and Raster3 D [89].

### Correlated mutations and sequence entropy

For the intramolecular correlated mutation analysis, correlated mutation positions in a given protein were compared with the homologous positions in all other proteins, and these pairs were either designated 'conserved' correlated mutation if they showed a correlated mutation as well, and 'non-conserved' correlated mutation if they did not. To compare sequence variability at 'conserved' vs 'non-conserved' sites, sequence entropy was used, which for alignment column *k *is defined as

$$S_{k}=-\sum_{j=1,20}P_{jk}\ln P_{jk}$$

where P_jk _is the frequency of amino acid *j *at position *k*.

### Randomization trials

Here we describe the various random trials that were performed in order to test for statistical significance. To assess the statistical significance of the intramolecular distance enrichment, 1000 random subsets of residue pairs were generated (with the size of the subset equal to the number of correlated mutation residue pairs). For these, the fraction of residues within 15Å of each other was calculated.

To assess the significance of observed intermolecular short distance enrichment for correlated mutation positions, we applied a randomization procedure where the original pairs of sequences that formed an input set for CAPS were randomly shuffled. This was repeated 1000 times.

To assess the significance of the observed overlap between correlated mutation residues and predicted interaction motifs, random 'correlated mutation' pairs were generated by replacing each position in an observed correlated mutation position pair with a randomly generated sequence position. In doing so, we took into account that a position could occur in several correlated mutation pairs; such position was replaced by the same random position in all its correlated mutation position pairs.

Finally, to assess the statistical significance of the observed preferred sequence-distance for correlated mutation positions within helices in the K-domain, we analyzed whether similar preferred sequence-distances occurred within randomly generated stretches of the sequence. The number and length distribution of these stretches was similar to that of the predicted K-domain coils, but their position within the sequence was randomized.

## Authors' contributions

ADJvD and RCHJvH conceived and designed the study, and wrote the paper. ADJvD performed the experiments and analyzed the data. All authors read and approved the final manuscript

## Supplementary Material

Additional file 1 **Sequence identifiers for intramolecular correlated mutation analysis**. This file contains the sequences identifiers of the sequences used in the intramolecular correlated mutation analysis. Click here for file

Additional file 2 **Input data for intermolecular correlated mutation analysis**. This file contains the pairs of interacting proteins used for the intermolecular correlated mutation analysis. Click here for file

Additional file 3 **Intramolecular correlated mutation results**. This files contains the correlated mutation pairs obtained from the intramolecular analysis. Click here for file

Additional file 4 **Intermolecular correlated mutation results**. This files contains the correlated mutation pairs obtained from the intermolecular analysis. Click here for file

Additional file 5 **Short distance enrichment significance**. This file contains an analysis of the statistical significance of the observed short distance enrichment for the intermolecular correlated mutations. Click here for file

Additional file 6 **Correlated mutations that have consistent overlap with predicted interaction motifs**. This file contains correlated mutations that have consistent overlap with predicted interaction motifs and are strong candidates to be important residues for protein-protein interactions of the MADS domain proteins. Click here for file

Additional file 7 **Conserved correlated mutations**. This file contains correlated mutations which are conserved, i.e. appearing in more than one MADS domain protein (both intramolecular and intermolecular correlated mutations). Click here for file

Additional file 8 **Prediction of helices in K-domain**. This file contains predicted helices in the K-domain. Click here for file

Additional file 9 **Predicted intramolecular contacts between helices**. This file contains contacts predicted between K-domain helices using correlated mutations. Click here for file

Additional file 10 **Predicted intramolecular contacts helices - MADS/I domain**. This file contains contacts predicted between K-domain helices and the MADS/I domain. Click here for file

Additional file 11 **Correlated mutations based analysis of intermolecular MADS domain protein helix - helix interactions**. This file contains an analysis of intermolecular contacts predicted between K-domain helices. Click here for file

## References

[B1] Altschuh D Lesk AM Bloomer AC Klug A Correlation of Coordinated Amino-Acid Substitutions with Function in Viruses Related to Tobacco Mosaic-Virus Journal of Molecular Biology 1987 193 4 693 707 10.1016/0022-2836(87)90352-4 3612789

[B2] Socolich M Lockless SW Russ WP Lee H Gardner KH Ranganathan R Evolutionary information for specifying a protein fold Nature 2005 437 7058 512 518 10.1038/nature03991 16177782

[B3] Fuchs A Martin-Galiano AJ Kalman M Fleishman S Ben-Tal N Frishman D Co-evolving residues in membrane proteins Bioinformatics 2007 23 24 3312 3319 10.1093/bioinformatics/btm515 18065429

[B4] Travers SAA Tully DC McCormack GP Fares MA A study of the coevolutionary patterns operating within the env gene of the HIV-1 group M subtypes Molecular Biology and Evolution 2007 24 12 2787 2801 10.1093/molbev/msm213 17921487

[B5] Kuipers RKP Joosten HJ Verwiel E Paans S Akerboom J van der Oost J Leferink NGH van Berkel WJH Vriend G Schaap PJ Correlated mutation analyses on super-family alignments reveal functionally important residues Proteins-Structure Function and Bioinformatics 2009 76 3 608 616 10.1002/prot.22374 19274741

[B6] Miller CS Eisenberg D Using inferred residue contacts to distinguish between correct and incorrect protein models Bioinformatics 2008 24 14 1575 1582 10.1093/bioinformatics/btn248 18511466 PMC2638260

[B7] Afonnikov DA Kolchanov NA CRASP: a program for analysis of coordinated substitutions in multiple alignments of protein sequences Nucleic Acids Research 2004 32 W64 W68 10.1093/nar/gkh451 15215352 PMC441589

[B8] Fares MA Travers SAA A novel method for detecting intramolecular coevolution: Adding a further dimension to selective constraints analyses Genetics 2006 173 1 9 23 10.1534/genetics.105.053249 16547113 PMC1461439

[B9] Martin LC Gloor GB Dunn SD Wahl LM Using information theory to search for co-evolving residues in proteins Bioinformatics 2005 21 22 4116 4124 10.1093/bioinformatics/bti671 16159918

[B10] Buslje CM Santos J Delfino JM Nielsen M Correction for phylogeny, small number of observations and data redundancy improves the identification of coevolving amino acid pairs using mutual information Bioinformatics 2009 25 9 1125 1131 10.1093/bioinformatics/btp135 19276150 PMC2672635

[B11] Buck MJ Atchley WR Networks of coevolving sites in structural and functional domains of serpin proteins Molecular Biology and Evolution 2005 22 7 1627 1634 10.1093/molbev/msi157 15858204

[B12] Horner DS Pirovano W Pesole G Correlated substitution analysis and the prediction of amino acid structural contacts Briefings in Bioinformatics 2008 9 1 46 56 10.1093/bib/bbm052 18000015

[B13] Fodor AA Aldrich RW Influence of conservation on calculations of amino acid covariance in multiple sequence alignments Proteins-Structure Function and Bioinformatics 2004 56 2 211 221 10.1002/prot.20098 15211506

[B14] Halperin I Wolfson H Nussinov R Correlated mutations: Advances and limitations. A study on fusion proteins and on the cohesin-dockerin families Proteins-Structure Function and Bioinformatics 2006 63 4 832 845 10.1002/prot.20933 16508975

[B15] Pazos F Valencia A Similarity of phylogenetic trees as indicator of protein-protein interaction Protein Engineering 2001 14 9 609 614 10.1093/protein/14.9.609 11707606

[B16] Goh CS Bogan AA Joachimiak M Walther D Cohen FE Co-evolution of proteins with their interaction partners Journal of Molecular Biology 2000 299 2 283 293 10.1006/jmbi.2000.3732 10860738

[B17] Sato T Yamanishi Y Horimoto K Kanehisa M Toh H Partial correlation coefficient between distance matrices as a new indicator of protein-protein interactions Bioinformatics 2006 22 20 2488 2492 10.1093/bioinformatics/btl419 16882650

[B18] Pazos F Ranea JAG Juan D Sternberg MJE Assessing protein co-evolution in the context of the tree of life assists in the prediction of the interactome Journal of Molecular Biology 2005 352 4 1002 1015 10.1016/j.jmb.2005.07.005 16139301

[B19] Izarzugaza JMG Juan D Pons C Ranea JAG Valencia A Pazos F TSEMA: interactive prediction of protein pairings between interacting families Nucleic Acids Research 2006 34 W315 W319 10.1093/nar/gkl112 16845017 PMC1538787

[B20] Ramani AK Marcotte EM Exploiting the co-evolution of interacting proteins to discover interaction specificity Journal of Molecular Biology 2003 327 1 273 284 10.1016/S0022-2836(03)00114-1 12614624

[B21] Waddell PJ Kishino H Ota R Phylogenetic methodology for detecting protein interactions Molecular Biology and Evolution 2007 24 3 650 659 10.1093/molbev/msl193 17158779

[B22] Juan D Pazos F Valencia A High-confidence prediction of global interactomes based on genome-wide coevolutionary networks Proceedings of the National Academy of Sciences of the United States of America 2008 105 3 934 939 10.1073/pnas.0709671105 18199838 PMC2242690

[B23] Hakes L Lovell SC Oliver SG Robertson DL Specificity in protein interactions and its relationship with sequence diversity and coevolution Proceedings of the National Academy of Sciences of the United States of America 2007 104 19 7999 8004 10.1073/pnas.0609962104 17468399 PMC1876561

[B24] Pazos F Valencia A Protein co-evolution, co-adaptation and interactions Embo Journal 2008 27 20 2648 2655 10.1038/emboj.2008.189 18818697 PMC2556093

[B25] Noivirt O Eisenstein M Horovitz A Detection and reduction of evolutionary noise in correlated mutation analysis Protein Engineering Design & Selection 2005 18 5 247 253 10.1093/protein/gzi029 15911538

[B26] Fraser HB Hirsh AE Wall DP Eisen MB Coevolution of gene expression among interacting proteins Proceedings of the National Academy of Sciences of the United States of America 2004 101 24 9033 9038 10.1073/pnas.0402591101 15175431 PMC439012

[B27] Angenent G de Folter S Nougalli I Immink R Protein complexes make the flower Comparative Biochemistry and Physiology a-Molecular & Integrative Physiology 2006 143 4 S167 S167

[B28] Ng M Yanofsky MF Function and evolution of the plant MADS-box gene family Nat Rev Genet 2001 2 3 186 195 10.1038/35056041 11256070

[B29] Immink RGH Angenent GC Transcription factors do it together: the hows and whys of studying protein-protein interactions Trends in Plant Science 2002 7 12 531 534 10.1016/S1360-1385(02)02343-9 12475492

[B30] de Folter S Immink RGH Kieffer M Parenicova L Henz SR Weigel D Busscher M Kooiker M Colombo L Kater MM Comprehensive interaction map of the Arabidopsis MADS box transcription factors Plant Cell 2005 17 5 1424 1433 10.1105/tpc.105.031831 15805477 PMC1091765

[B31] Parenicova L de Folter S Kieffer M Horner DS Favalli C Busscher J Cook HE Ingram RM Kater MM Davies B Molecular and phylogenetic analyses of the complete MADS-box transcription factor family in Arabidopsis: New openings to the MADS world Plant Cell 2003 15 7 1538 1551 10.1105/tpc.011544 12837945 PMC165399

[B32] De Bodt S Raes J Van de Peer YV Theissen G And then there were many: MADS goes genomic Trends in Plant Science 2003 8 10 475 483 10.1016/j.tplants.2003.09.006 14557044

[B33] Han AD Pan F Stroud JC Youn HD Liu JO Chen L Sequence-specific recruitment of transcriptional co-repressor Cabin1 by myocyte enhancer factor-2 Nature 2003 422 6933 730 734 10.1038/nature01555 12700764

[B34] Pellegrini L Tan S Richmond TJ Dominguez R Souchon H Spinelli S Dauter Z Wilson KS Chauvaux S Beguin P Structure of serum response factor core bound to DNA Nature 1995 376 6540 490 498 10.1038/376490a0 7637780

[B35] Santelli E Richmond TJ Crystal structure of MEF2A core bound to DNA at 1.5 A resolution J Mol Biol 2000 297 2 437 449 10.1006/jmbi.2000.3568 10715212

[B36] Huang K Louis JM Donaldson L Lim FL Sharrocks AD Clore GM Solution structure of the MEF2A-DNA complex: structural basis for the modulation of DNA bending and specificity by MADS-box transcription factors Embo J 2000 19 11 2615 2628 10.1093/emboj/19.11.2615 10835359 PMC212754

[B37] Mo Y Ho W Johnston K Marmorstein R Crystal structure of a ternary SAP-1/SRF/c-fos SRE DNA complex J Mol Biol 2001 314 3 495 506 10.1006/jmbi.2001.5138 11846562

[B38] Hassler M Richmond TJ The B-box dominates SAP-1-SRF interactions in the structure of the ternary complex Embo J 2001 20 12 3018 3028 10.1093/emboj/20.12.3018 11406578 PMC150215

[B39] Krizek BA Meyerowitz EM Mapping the protein regions responsible for the functional specificities of the Arabidopsis MADS domain organ-identity proteins Proceedings of the National Academy of Sciences of the United States of America 1996 93 9 4063 4070 10.1073/pnas.93.9.4063 8633017 PMC39487

[B40] Riechmann JL Krizek BA Meyerowitz EM Dimerization specificity of Arabidopsis MADS domain homeotic proteins APETALA1, APETALA3, PISTILLATA, and AGAMOUS Proceedings of the National Academy of Sciences of the United States of America 1996 93 10 4793 4798 10.1073/pnas.93.10.4793 8643482 PMC39358

[B41] Hill K Wang H Perry SE A transcriptional repression motif in the MADS factor AGL15 is involved in recruitment of histone deacetylase complex components Plant Journal 2008 53 1 172 185 10.1111/j.1365-313X.2007.03336.x 17999645

[B42] Lim J Moon YH An G Jang SK Two rice MADS domain proteins interact with OsMADS1 Plant Molecular Biology 2000 44 4 513 527 10.1023/A:1026517111843 11197326

[B43] Yang YZ Jack T Defining subdomains of the K domain important for protein-protein interactions of plant MADS proteins Plant Molecular Biology 2004 55 1 45 59 10.1007/s11103-004-0416-7 15604664

[B44] Yang YZ Fanning L Jack T The K domain mediates heterodimerization of the Arabidopsis floral organ identity proteins, APETALA3 and PISTILLATA Plant Journal 2003 33 1 47 59 10.1046/j.0960-7412.2003.01473.x 12943540

[B45] Kaufmann K Anfang N Saedler H Theissen G Mutant analysis, protein-protein interactions and subcellular localization of the Arabidopsis B-sister (ABS) protein Molecular Genetics and Genomics 2005 274 2 103 118 10.1007/s00438-005-0010-y 16080001

[B46] Martinez-Castilla LP Alvarez-Buylla ER Adaptive evolution in the Arabidopsis MADS-box gene family inferred from its complete resolved phylogeny Proceedings of the National Academy of Sciences of the United States of America 2003 100 23 13407 13412 10.1073/pnas.1835864100 14597714 PMC263827

[B47] Nam J Kaufmann K Theiben G Nei M A simple method for predicting the functional differentiation of duplicate genes and its application to MIKC-type MADS-box genes Nucleic Acids Research 2005 33 2 10.1093/nar/gki978 PMC548370 15659573

[B48] Hernandez-Hernandez T Martinez-Castilla LP Alvarez-Buylla ER Functional diversification of B MADS-Box homeotic regulators of flower development: Adaptive evolution in protein-protein interaction domains after major gene duplication events Molecular Biology and Evolution 2007 24 2 465 481 10.1093/molbev/msl182 17135333

[B49] Veron AS Kaufmann K Bornberg-Bauer E Evidence of interaction network evolution by whole-genome duplications: A case study in MADS-box proteins Molecular Biology and Evolution 2007 24 3 670 678 10.1093/molbev/msl197 17175526

[B50] Lenser T Theissen G Dittrich P Developmental Robustness by Obligate Interaction of Class B Floral Homeotic Genes and Proteins Plos Computational Biology 2009 5 1 10.1371/journal.pcbi.1000264 19148269 PMC2612583

[B51] Espinosa-soto C Padilla-Longoria P Alvarez-Buylla ER A gene regulatory network model for cell-fate determination during Arabidopsis thalianal flower development that is robust and recovers experimental gene expression profiles Plant Cell 2004 16 11 2923 2939 10.1105/tpc.104.021725 15486106 PMC527189

[B52] Mendoza L Thieffry D Alvarez-Buylla ER Genetic control of flower morphogenesis in Arabidopsis thaliana: a logical analysis Bioinformatics 1999 15 7-8 593 606 10.1093/bioinformatics/15.7.593 10487867

[B53] van Dijk ADJ Ter Braak CJF Immink RG Angenent GC van Ham RCHJ Predicting and understanding transcription factor interactions based on sequence level determinants of combinatorial control Bioinformatics 2008 24 1 26 33 10.1093/bioinformatics/btm539 18024974

[B54] van Dijk ADJ Morabito G Fiers M Van Ham RCHJ Angenent GC Immink RGH Sequence motifs in MADS transcription factors responsible for specificity and diversification of protein-protein interaction Plos Computational Biology in press 10.1371/journal.pcbi.1001017 PMC2991254 21124869

[B55] Yu HY Luscombe NM Lu HX Zhu XW Xia Y Han JDJ Bertin N Chung S Vidal M Gerstein M Annotation transfer between genomes: Protein-protein interologs and protein-DNA regulogs Genome Research 2004 14 6 1107 1118 10.1101/gr.1774904 15173116 PMC419789

[B56] Mika S Rost B Protein-protein interactions more conserved within species than across species Plos Computational Biology 2006 2 7 698 709 10.1371/journal.pcbi.0020079 PMC1513270 16854211

[B57] Clark RM Schweikert G Toomajian C Ossowski S Zeller G Shinn P Warthmann N Hu TT Fu G Hinds DA Common sequence polymorphisms shaping genetic diversity in Arabidopsis thaliana Science 2007 317 5836 338 342 10.1126/science.1138632 17641193

[B58] Kaufmann K Melzer R Theissen G MIKC-type MADS-domain proteins: structural modularity, protein interactions and network evolution in land plants Gene 2005 347 2 183 198 10.1016/j.gene.2004.12.014 15777618

[B59] Melzer R Theissen G Reconstitution of floral quartets in vitro involving class B and class E floral homeotic proteins Nucleic Acids Research 2009 37 8 2723 2736 10.1093/nar/gkp129 19276203 PMC2677882

[B60] Immink RGH Tonaco IAN de Folter S Shchennikova A van Dijk ADJ Busscher-Lange J Borst JW Angenent GC SEPALLATA3: the 'glue' for MADS box transcription factor complex formation Genome Biology 2009 10 2 10.1186/gb-2009-10-2-r24 PMC2688274 19243611

[B61] Ciannamea S Kaufmann K Frau M Tonaco IAN Petersen K Nielsen KK Angenent GC Immink RGH Protein interactions of MADS box transcription factors involved in flowering in Lolium perenne Journal of Experimental Botany 2006 57 13 3419 3431 10.1093/jxb/erl144 17005923

[B62] Cseke LJ Ravinder N Pandey AK Podila GK Identification of PTM5 protein interaction partners, a MADS-box gene involved in aspen tree vegetative development Gene 2007 391 1-2 209 222 10.1016/j.gene.2006.12.033 17331677

[B63] Fornara F Parenicova L Falasca G Pelucchi N Masiero S Ciannamea S Lopez-Dee Z Altamura MM Colombo L Kater MM Functional characterization of OsMADS18, a member of the AP1/SQUA subfamily of MADS box genes Plant Physiology 2004 135 4 2207 2219 10.1104/pp.104.045039 15299121 PMC520791

[B64] Kane NA Danyluk J Tardif G Ouellet F Laliberte JF Limin AE Fowler DB Sarhan F TaVRT-2, a member of the StMADS-11 clade of flowering repressors, is regulated by vernalization and photoperiod in wheat Plant Physiology 2005 138 4 2354 2363 10.1104/pp.105.061762 16024692 PMC1183421

[B65] Shan HY Su KM Lu WL Kong HZ Chen ZD Meng Z Conservation and divergence of candidate class B genes in Akebia trifoliata (Lardizabalaceae) Development Genes and Evolution 2006 216 12 785 795 10.1007/s00427-006-0107-2 17086426

[B66] Shitsukawa N Tahira C Kassai KI Hirabayashi C Shimizu T Takumi S Mochida K Kawaura K Ogihara Y Murai K Genetic and epigenetic alteration among three homoeologous genes of a class E MADS box gene in hexaploid wheat Plant Cell 2007 19 6 1723 1737 10.1105/tpc.107.051813 17586655 PMC1955732

[B67] Sundstrom J Engstrom P Conifer reproductive development involves B-type MADS-box genes with distinct and different activities in male organ primordia Plant Journal 2002 31 2 161 169 10.1046/j.1365-313X.2002.01343.x 12121446

[B68] Mulder NJ Apweiler R Attwood TK Bairoch A Bateman A Binns D Bork P Buillard V Cerutti L Copley R New developments in the InterPro database Nucleic Acids Research 2007 35 D224 D228 10.1093/nar/gkl841 17202162 PMC1899100

[B69] Bairoch A Consortium U Bougueleret L Altairac S Amendolia V Auchincloss A Argoud-Puy G Axelsen K Baratin D Blatter MC The Universal Protein Resource (UniProt) 2009 Nucleic Acids Research 2009 37 D169 D174 10.1093/nar/gkn664 18836194 PMC2686606

[B70] Eddy SR Profile hidden Markov models Bioinformatics 1998 14 9 755 763 10.1093/bioinformatics/14.9.755 9918945

[B71] Goff SA Ricke D Lan TH Presting G Wang RL Dunn M Glazebrook J Sessions A Oeller P Varma H A draft sequence of the rice genome (Oryza sativa L. ssp japonica) Science 2002 296 5565 92 100 10.1126/science.1068275 11935018

[B72] Tuskan GA DiFazio S Jansson S Bohlmann J Grigoriev I Hellsten U Putnam N Ralph S Rombauts S Salamov A The genome of black cottonwood, Populus trichocarpa (Torr. & Gray) Science 2006 313 5793 1596 1604 10.1126/science.1128691 16973872

[B73] Velasco R Zharkikh A Troggio M Cartwright DA Cestaro A Pruss D Pindo M FitzGerald LM Vezzulli S Reid J A High Quality Draft Consensus Sequence of the Genome of a Heterozygous Grapevine Variety PLoS ONE 2007 2 12 e1326 10.1371/journal.pone.0001326 18094749 PMC2147077

[B74] Rensing SA Lang D Zimmer AD Terry A Salamov A Shapiro H Nishiyama T Perroud PF Lindquist EA Kamisugi Y The Physcomitrella genome reveals evolutionary insights into the conquest of land by plants Science 2008 319 5859 64 69 10.1126/science.1150646 18079367

[B75] Ming R Hou SB Feng Y Yu QY Dionne-Laporte A Saw JH Senin P Wang W Ly BV Lewis KLT The draft genome of the transgenic tropical fruit tree papaya (Carica papaya Linnaeus) Nature 2008 452 7190 991 U997 10.1038/nature06856 18432245 PMC2836516

[B76] Paterson AH Bowers JE Bruggmann R Dubchak I Grimwood J Gundlach H Haberer G Hellsten U Mitros T Poliakov A The Sorghum bicolor genome and the diversification of grasses Nature 2009 457 7229 551 556 10.1038/nature07723 19189423

[B77] Edgar RC MUSCLE: multiple sequence alignment with high accuracy and high throughput Nucleic Acids Research 2004 32 5 1792 1797 10.1093/nar/gkh340 15034147 PMC390337

[B78] Ashkenazy H Unger R Kliger Y Optimal data collection for correlated mutation analysis Proteins-Structure Function and Bioinformatics 2009 74 3 545 555 10.1002/prot.22168 18655065

[B79] Shan HY Zhan N Liu CJ Xu GX Zhang J Chen ZD Kong HZ Patterns of gene duplication and functional diversification during the evolution of the AP1/SQUA subfamily of plant MADS-box genes Molecular Phylogenetics and Evolution 2007 44 1 26 41 10.1016/j.ympev.2007.02.016 17434760

[B80] Henikoff S Henikoff JG Amino-Acid Substitution Matrices from Protein Blocks Proceedings of the National Academy of Sciences of the United States of America 1992 89 22 10915 10919 10.1073/pnas.89.22.10915 1438297 PMC50453

[B81] Travers SAA Fares MA Functional coevolutionary networks of the Hsp70-Hop-Hsp90 system revealed through computational analyses Molecular Biology and Evolution 2007 24 4 1032 1044 10.1093/molbev/msm022 17267421

[B82] Thompson JD Higgins DG Gibson TJ Clustal-W - Improving the Sensitivity of Progressive Multiple Sequence Alignment through Sequence Weighting, Position-Specific Gap Penalties and Weight Matrix Choice Nucleic Acids Research 1994 22 22 4673 4680 10.1093/nar/22.22.4673 7984417 PMC308517

[B83] Caporaso JG Smit S Easton BC Hunter L Huttley GA Knight R Detecting coevolution without phylogenetic trees? Tree-ignorant metrics of coevolution perform as well as tree-aware metrics Bmc Evolutionary Biology 2008 8: 10.1186/1471-2148-8-327 19055758 PMC2637866

[B84] Becker A Theissen G The major clades of MADS-box genes and their role in the development and evolution of flowering plants Molecular Phylogenetics and Evolution 2003 29 3 464 489 10.1016/S1055-7903(03)00207-0 14615187

[B85] Lupas A Vandyke M Stock J Predicting Coiled Coils from Protein Sequences Science 1991 252 5009 1162 1164 10.1126/science.252.5009.1162 2031185

[B86] Sali A Blundell TL Comparative Protein Modeling by Satisfaction of Spatial Restraints Journal of Molecular Biology 1993 234 3 779 815 10.1006/jmbi.1993.1626 8254673

[B87] Brunger AT Adams PD Clore GM DeLano WL Gros P Grosse-Kunstleve RW Jiang JS Kuszewski J Nilges M Pannu NS Crystallography & NMR system: A new software suite for macromolecular structure determination Acta Crystallographica Section D-Biological Crystallography 1998 54 905 921 10.1107/S0907444998003254 9757107

[B88] Kraulis PJ Molscript - a Program to Produce Both Detailed and Schematic Plots of Protein Structures Journal of Applied Crystallography 1991 24 946 950 10.1107/S0021889891004399

[B89] Merritt EA Murphy MEP Raster3 d Version-2.0 - a Program for Photorealistic Molecular Graphics Acta Crystallographica Section D-Biological Crystallography 1994 50 869 873 10.1107/S0907444994006396 15299354

