# Supplementary material for: Conserved and variable correlated mutations in the plant MADS protein network
Source: BMC Genomics. 2010 Oct 28;11:607. doi: 10.1186/1471-2164-11-607 (PMC3017862; doi:10.1186/1471-2164-11-607)
Supplement: Additional file 1 — Sequence identifiers for intramolecular correlated mutation analysis. This file contains the sequences identifiers of the sequences used in the intramolecular correlated mutation analysis. [file 1471-2164-11-607-S1.DOC]

**Additional File 1. Sequence identifiers for sequences used in correlated mutation analysis.^a^**

**AG**

155967404_102107

Q05KK3_55188

Q8GUZ3_3711

Q40168_4081

Q8VWZ2_3750

Q6EM18_3719

183014293_68875

Q40885_4102

O64958_3659

GRMZM2G059402_P01_4577

1006768_4577

195622196_4577

Q84V75_4577

Q6S6L9_257611

Q6S6M0_257611

48727610_155132

PTAG1_3694

Q41352_37657

Q2NNC2_51953

Q2NNC3_51953

Q533S1_34305

Q533S0_34305

Q2XUP3_148910

209414516_23110

Q948V3_81865

Q2WBM7_102599

A2Y1E3_4530

LOC_OS01G10504_4530

Q2V0P1_4530

O65802_49495

115441497_39947

91207156_39947

115462579_39947

Q6S6N0_3527

193248815_487795

Q5G0F8_46970

197690821_375287

161158766_4565

Q1G170_4565

Q1G188_4565

Q8L5F4_4039

Q3YAG2_60419

Q84LC3_4232

Q8GTY3_4232

Q690M8_3562

Q6S6K9_79245

IMGA_AC153460_43.2_3880

Q2TDX7_13098

Q9ZPK9_82025

148540542_4407

148540540_4407

Q56NI3_3888

602900_52853

22091479_79200

Q2TUV5_3847

Q64FN4_3760

A1EAG0_161934

Q9MBD9_74645

Q6S6L7_218854

Q8RVK1_3635

A2IBU9_3635

Q76N62_35883

Q6S6L0_218853

Q6S6L2_218850

Q68RI3_125259

Q2TDX6_77108

Q5G0F2_46969

Sb03g002525.1_4558

Q1WG48_3673

Q84LD1_41568

Q2FC26_117978

Q6RFR2_4690

Q6S6K6_264188

Q6S6M6_3472

Q8RU44_4513

171194269_4513

A3QQT3_3435

A3QQT4_3435

A3QQS3_3436

A3QQS2_3436

SIM4.ALN-TCVV002732_29760

147853709_29760

19698536_112509

Q6S6M7_16752

Q43585_4097

94983051_164110

Q93XL1_51240

Q6S6M5_257523

Q9SBT4_3747

33772667_13288

33772657_228869

Q01540_3708

Q9ZS29_18101

Q9ZS30_18101

1345505_3702

Q4PRG5_3707

Q6EM05_228867

Q6EM10_228867

Q2N2U2_3467

Q6S6L4_171898

Q6S6L5_171898

Q9XFM8_4151

33772671_29727

33772663_29727

Q2TDX5_13333

Q6S6L6_13331

Q9LEP2_3505

26517024_51351

A3F6M9_105599

Q6Q6W8_82528

Q42457_41241

Q2IA04_51096

Q40872_4054

**AGL6**

23304692_3715

Q1G172_4565

Q8GTE8_3712

O82699_3750

212525794_3635

Q7XBI8_34270

110164822_3435

110164824_3435

Q9MB91_4102

Sb04g031750.1_4558

GRMZM2G003514_P01_4577

Q1KTF3_3673

168020161_145481

168055719_145481

Q84LC8_41568

Q9XGJ6_3382

190183773_3369

94754_3218

Q6QHI1_4513

A2X8C5_4530

13442962_4530

LOC_OS02G45770_4530

Q40970_3347

Q7XBL8_13612

Q7XBL9_13612

Q400H6_51953

Q2NNC0_51953

A0MTC2_82528

FGENESH.VV78X028385.7_1_29760

44888603_112509

Q7XAP8_16752

PMADS34_3694

218191365_39946

115447939_39947

115460098_39947

Q6EUV6_18101

evm.TU.supercontig_414.3_3649

Q2TDX3_3406

Q58A75_3311

154482028_391288

160713959_391288

Q6TXR3_4686

Q56NI6_3888

Q56NI5_3888

IMGA_AC147014_36.2_3880

Q6J554_257763

Q2TDX2_13333

Q508G3_4641

Q84UA0_4522

Q948V2_81865

Q948V1_81865

Q50H38_82025

223548945_3988

Q84L85_186128

P93468_54921

**AP1**

887392_3715

Q6EEV9_2711

A2IB53_2711

evm.TU.supercontig_1.158_3649

Q39371_3712

146160688_94219

Q0PPV5_3750

O82695_3750

183014287_68875

Q7XBK6_4102

GRMZM2G112947_P01_4577

32478017_50994

20799370_59689

Q0PLP3_15368

Q0PLP5_15368

A1XRM8_140101

A1XRM9_140101

Q9SQJ8_3693

Q9SQJ9_3693

15824795_4530

LOC_OS03G54160_4530

Q6Q9I2_4530

A2YHF1_4530

41323982_3696

Q41355_37657

Q400H7_51953

Q6EJA4_13006

Q533S7_34305

Q533S8_34305

219964711_29780

219964713_29780

Q9SNX1_84618

1561778_36774

Q2WBM2_102599

659188_3694

719996_3694

47934199_3694

PTAP1-2_3694

Q2XUP6_148910

Q2XUP7_148910

Q9ZTT6_34176

Q9ZTT7_34176

218198968_39946

158517760_39946

33146447_39947

Q7XBL4_3527

32478045_3527

32478051_3527

193248813_487795

Q41276_3728

32478105_59016

Q7XBI5_59016

Q7XBI4_59016

Q7XBI3_59016

Q8L5F7_4039

Q7XBI2_45171

Q84LC0_4232

Q8RVR0_4232

194500615_3562

194500617_3562

217072094_3880

IMGA_AC144726_1.2_3880

148540532_4407

A1IIU5_225117

Q9AR13_3888

602906_52853

22091473_79200

32478015_71251

156454654_3760

Q0PLN6_4498

Q0PLN5_4498

Q8S4L3_4081

Q8S4L5_4081

Q7XBJ6_74828

Q7XBJ4_74828

Q7XBJ3_74828

Q7XBL1_74825

Q7XBL2_74825

A1XRM7_74825

A1XRM5_4432

Q1G179_4565

Q1G194_4565

Q7XBJ1_34270

Q7XBK1_74823

Q7XBK0_74823

Q2TDY3_77108

Sb02g001090.1_4558

O04068_4558

Q0PLQ2_38733

Q84LD5_41568

Q84LD6_41568

Q9XHR6_4096

205345279_35883

Q0PLN2_29674

Q9XGJ4_3382

Q70ZY4_60874

Q9LEI0_4513

A1XRM0_4002

A1XRL8_4002

A1XRL9_4002

145693005_3767

145693003_3767

Q0PLM9_38735

Q0PLM6_4555

Q0PLM5_4555

Q3YL56_148545

SIM4.ALN-TCVV015011_29760

9367313_112509

Q0PLP8_96049

Q1ZZ73_171567

Q9XHR8_4097

Q4G282_117978

Q689E7_55190

A4GVT9_97445

Q4PLC4_75709

Q9ZS25_18101

Q0PLP7_38706

Q0PLM2_4543

154551051_359415

Q38742_4151

Q1ZZ77_376443

Q39400_3505

Q39399_3505

Q7XBJ8_3469

Q84UA3_4522

Q84UA2_4522

**AP3**

1621333_3715

Q05KJ9_55188

Q6IV03_3711

Q9XHT7_3711

40644780_4565

O82130_4565

Q96359_3712

Q9SX16_3712

Q96358_3712

O65145_3415

Q58IJ2_3659

GRMZM2G139073_P01_4577

Q6T4U0_3419

Q6T4U1_3419

Q6T4U2_3419

Q6T4U3_3419

5825625_102746

186909205_13569

Q6GWV3_155132

Q6GWV2_155132

Q0ZPQ3_155132

148913111_4232

Q84LC1_4232

148913109_4232

Q84Y21_46993

Q157N2_227724

Q157P5_227724

Q533S3_34305

Q533S2_34305

Q9SWL0_76672

1561786_36774

1561782_36774

Q948U8_81865

Q5KTK2_124794

Q5KTK3_124794

Q2WBM8_102599

A3BFC0_4530

LOC_OS06G49840_4530

A2YGX6_4530

Q68BH1_4424

Q8H2C4_54806

223588249_39946

223588253_39947

222636213_39947

193248817_487795

147868421_3649

evm.TU.supercontig_6.195_3649

37993055_3406

Q8L5F5_4039

186909231_3449

186909229_3449

Q1HLD4_45171

Q1HLD3_45171

Q6QVY1_260612

Q6QVY0_260612

Q9LLA8_49456

Q6E215_3562

148734373_13216

148734369_13216

Q2TDY0_13098

Q2TDX9_13098

Q2TDY1_13098

186909167_13523

89887324_175104

Q6T4U9_213843

Q6QVX9_262426

Q6QVY6_262426

Q84Y44_4403

Q84Y43_4403

Q84Y34_46983

Q84Y31_46983

Q84Y33_46983

37993051_13521

Q6T4V0_13258

148734367_447319

Q5VKS3_3847

148734363_35927

148734361_35927

Q19R21_255211

Q93X10_74645

Q7X9I8_74645

91106158_4407

Q9XF52_74828

O65143_74828

O65138_74825

O65137_74825

O65146_74826

Q9SWK8_76686

Q9SWK7_76687

O65141_74823

O65140_74823

Q2QL32_77108

Q84Y61_64040

Q84Y62_64040

Q84Y63_64040

602904_52853

Sb10g029810.1_4558

Q6QPY3_188307

Q6QPY7_188307

Q5XKT4_200642

Q5SBH5_158555

Q5SBH4_158555

Q9XF51_3472

Q84M22_13306

Q84M21_13306

Q68BH7_4414

Q42498_3570

O65144_13612

Q41477_4113

22091477_79200

A1XSX7_311263

A1XSX8_311263

Q157N9_103475

Q157N4_103475

37993049_251264

Q157P4_389206

Q157N7_389206

Q157N6_389207

Q157P6_389207

44888599_112509

186909213_46987

186909211_46987

186909209_46987

Q5NU35_77340

Q5VJN0_3747

40646976_3747

Q6UVI1_118781

Q9LLA0_118780

A4L7M7_97445

Q19R25_127127

Q19R24_127127

Q157P0_258457

Q157P1_258457

Q157N5_258457

Q9ZS28_18101

Q9ZS27_18101

186909219_522534

186909217_522534

186909221_522534

Q6T4T6_114516

Q6T4T5_114516

Q6T4T3_114516

A3FJ53_3467

A3FJ54_3467

Q84Y51_171898

Q84Y50_171898

Q84Y48_171898

Q84Y49_171898

Q7XAQ4_16752

Q1HLE1_4298

Q1HLE0_4298

37993067_175201

37993069_175201

37993037_13333

Q68BH9_13333

Q84Y85_13331

Q84Y86_13331

Q6QVY3_69919

Q6QVX7_69919

Q68BH3_4426

Q50HZ1_3505

A3R0V8_3469

A3R0V7_3469

186909177_49683

186909175_49683

186909173_49683

186909171_49683

Q8LT10_82328

Q4KPI7_82528

Q42503_41241

Q42500_41241

186909183_522535

186909181_522535

Q84Y81_37489

Q84Y83_37489

Q84Y84_37489

Q1HLD8_45339

Q1HLD9_45339

Q9SWK3_4263

Q68BH5_126639

Q68A85_126639

Q8VWZ4_3750

Q8L5M8_3750

183014289_68875

Q0QCW8_4100

Q6PUJ5_4100

Q07472_4102

Q9LLA1_4102

Q1G4P5_4432

Q1HLE3_50993

Q1HLE2_50993

Q1HLE4_50993

6707088_59689

A4UU43_3451

A4UU42_3451

A4UU41_3451

Q157N3_310467

Q157P2_310467

Q6T4S2_257611

Q6T4S4_257611

Q6T4S5_257611

A4UTT8_140101

PMADS3_3694

Q2NNC1_51953

Q5NU33_4744

31879355_37657

Q41354_37657

Q7X9P4_37657

8163944_13006

Q6TH79_13006

Q2XUP5_148910

197244657_23110

Q9LLA3_23110

Q9LLA2_23110

Q940S7_37492

Q40352_3879

186909241_215066

186909237_215066

186909239_215066

Q6QVY2_79772

Q6QVY7_79772

A2PZF5_71825

A2PZF4_71825

8163942_3429

Q9LLA6_3429

Q84Y55_218853

Q84Y54_218853

Q1HLD1_3527

Q1HLD2_3527

150404770_4378

150404772_4378

Q5G0F5_46970

Q5G0F4_46970

Q5G0F3_46970

Q6QPY2_188319

Q6QPY6_188319

Q9SWK9_76685

A1XSX6_38723

Q4LEZ4_4686

O65121_79245

Q84Y42_79245

Q9XF53_79245

Q1HLD5_83909

Q1HLD6_83909

Q1HLD7_83909

Q68BG9_54801

223588215_4529

A1IIU3_225117

Q6T4S8_128639

Q6T4S9_128639

186909199_13762

186909197_13762

Q6QPY1_262469

Q6QPY5_262469

148734359_304186

Q6T4U5_12953

Q9SWK5_76702

Q9SWK6_76700

Q9SWK4_76705

O65142_41767

3170490_4081

Q2UVA8_4081

Q40171_4081

Q1ERG7_34198

213688850_52518

217337300_52518

Q84Y68_218854

Q84Y66_218854

Q9SWM1_4245

Q9SWL1_4245

Q6QVY9_34270

Q84Y75_218850

Q84Y74_218850

Q84Y76_218850

Q68RI4_125259

186909191_244027

186909193_244027

148734365_76097

Q84Y37_46969

Q84Y39_46969

Q84Y38_46969

Q6QPY8_188322

Q6QPY4_188322

A1XSX5_38733

186909187_46961

Q8LRS9_4690

Q1HLC9_264188

Q1HLD0_264188

Q157N8_33115

Q157P3_33115

Q6QHI3_4513

124484515_208882

124484513_208882

37993047_3435

215433735_136990

215433737_136990

Q84LD0_41568

Q84LC7_41568

Q6QVX8_39353

Q6QVY5_39353

Q9LL99_85283

IMGA_AC151483_32.2_3880

A3RJI1_29760

SIM4.ALN-TCVV004432_29760

Q69BL2_78828

Q69BL3_78828

Q69BL1_78828

Q69BL5_78828

Q40513_4097

Q93XL0_51240

Q6T4V5_257523

Q6T4V4_257523

Q6T4V6_257523

Q9SWK1_4254

Q6T4V1_257524

Q7XBE9_3708

Q7XBE8_3708

Q9XHU3_3708

87133584_3708

Q53US5_186128

Q710I2_76084

38229881_76084

154551055_359415

Q4PRG7_3707

Q7X9P3_39879

Q7X9P2_39879

Q5KTK1_105750

P23706_4151

Q7XJE0_4155

Q6QVY4_4155

42795301_4155

Q7XJD4_4158

Q7XJD6_4158

A3F6R0_105599

223526870_3988

Q2IA05_51096

Q2IA06_51096

218118130_342001

**FUL**

23304676_3715

23304674_3715

23304672_3715

Q7XBM1_4081

Q7XBM0_4081

Q8H284_4081

Q40170_4081

Q05KK1_55188

Q7XZQ5_224085

evm.TU.supercontig_14.227_3649

157674589_3649

Q8GTF8_3712

Q8GTF9_3712

Q8GTF7_3712

Q7X9I5_3750

Q9ZRA5_3750

Q283Q2_3750

32452882_3750

A1XRN0_41786

A1XRN1_41786

Q3KSZ1_3755

Q948U1_81865

Q948U5_81865

Q39401_3505

Q5ETU9_4571

Q9SBQ0_4102

Q9SBQ1_4102

32478069_4102

Q9ATE2_4102

Q7XBJ9_3469

Sb01g007790.1_4558

GRMZM2G112947_P04_4577

Q84V71_4577

150834521_200642

Q0Q5E9_4097

Q8GT99_4097

33342030_16752

Q7XBK9_72116

Q7XBK8_72116

Q84LD4_41568

32478019_50994

Q4G281_117978

Q84KI0_4568

Q0PLN4_29674

Q8L6I8_60874

Q56NI7_3888

Q6GWV1_155132

Q20CR2_4120

PMADS28_3694

661810_3694

195109_3694

A3QQT1_3435

Q7XBL7_13612

Q41356_37657

33309870_51953

Q400H8_51953

33309864_51953

Q2LE00_51953

Q400H9_51953

197244649_23110

197244651_23110

Q3YL55_148545

Q7XAT7_82528

33355661_82528

33355665_82528

Q58A73_3311

157356284_29760

SIM4.ALN-TCVV007083_29760

9367307_112509

9367309_112509

Q0PLN7_4498

Q64FN3_3760

O23765_58167

Q9XHR7_4096

A2YNI2_4530

Q0IN73_4530

LOC_OS07G41370_4530

188531744_35883

Q8H0J2_35883

205345277_35883

29825728_39947

115473253_39947

Q7XBL5_3527

Q1ZZV2_3708

Q41274_3728

Q42429_4113

O82128_4565

Q2TM81_3406

145334861_3702

Q3YAG0_60419

154551049_359415

Q0PLQ3_38723

A1XRM1_161261

A1XRM2_161261

194500619_3562

Q710M5_54806

Q7XBN7_4151

Q941M9_4151

Q710I1_76084

IMGA_AC137823_3.2_3880

217074016_3880

Q6TKR6_257763

A1XRM3_106141

A1XRM4_106141

32478013_71251

148540534_4407

Q84UA1_4522

602908_52853

Q9SEG7_4072

Q2TM82_13521

223546227_3988

Q9FUH8_34317

Q9FUI2_34317

Q9FUI3_34317

Q9LEI3_4513

Q58XC6_4513

**PI**

O64934_71139

Q68RI2_125259

186909185_522535

evm.TU.supercontig_26.318_3649

Q0PXC2_4081

Q1L7F1_4714

Q1L7F0_4714

Q9AVU6_3750

22775406_3750

Q84L10_3505

183014291_68875

O65131_3415

186909169_13523

Q40883_4102

Q6QNL4_4102

Q9ZTQ9_3659

GRMZM2G110153_P01_4577

O65128_34270

Q9SED8_3329

37993031_13521

Q710M4_54806

Q1L7E7_160690

Q1L7E8_160690

Q6T4T7_3419

Q6T4T8_3419

6707091_59689

A4UU40_3451

190183771_3369

Q93X05_3369

186909207_13569

Q6T4S1_257611

Q6GWU9_155132

Q68BH4_126639

PMADS11_3694

Q71TT0_51953

Q5K6A0_51953

154243317_3696

Q5NU34_4744

Q41353_37657

Q84Y19_46993

Q84Y20_46993

Q9LL95_13006

Q533S5_34305

Q533S4_34305

Q2XUP4_148910

197244659_23110

Q9LL94_23110

Q948U7_81865

Q940S6_37492

Q2WBM4_102599

50082561_3562

186909243_215066

O65133_74827

O65134_74827

O65132_74826

LOC_OS05G34940_4530

Q40703_4530

A2WXN3_4530

A2PZF6_71825

158853186_35883

Q84Y45_171898

Q84Y46_171898

Q84Y47_171898

218196828_39946

115463981_39947

115441465_39947

193248827_487795

Q5G0F7_46970

Q5G0F6_46970

197690833_375287

Q76DP9_4565

Q1G182_4565

Q2TM79_3406

Q8L5F6_4039

Q9M7M7_3347

A1XSY3_38723

Q8GTY2_4232

Q84LC2_4232

Q9LL98_49456

46020020_4686

Q76G51_4686

A3R0V9_3469

A3R0W0_3469

Q84Y40_79245

Q84Y41_79245

Q9XF55_79245

O65125_79245

148734379_304186

186909233_3449

Q68BG8_54801

Q68BG6_54801

Q68BG7_54801

Q2TDY2_13098

186909195_244027

89887326_175104

145843814_175104

148540538_4407

148540536_4407

Q6T4S6_128639

Q6T4S7_128639

186909203_13762

186909201_13762

Q5SBH2_158555

Q84Y26_46983

Q84Y24_46983

Q84Y25_46983

Q84Y22_46983

Q84Y29_46983

22091475_79200

Q6T4U7_13258

44888601_112509

152926225_3760

148734383_76097

218118120_342001

218118122_342001

148734375_35927

Q19R22_255211

Q84Y36_46969

O65124_41767

Q9FZN1_74645

Q6T4U8_213843

Q6T4V7_213843

O65127_74828

O65126_74828

Q1ERG8_34198

Q84Y65_218854

Q84Y64_218854

212525792_3635

Q6T4T1_114516

Q6T4T0_114516

Q84Y53_218853

Q84Y52_218853

Q84Y73_218850

O65123_74823

O65122_74823

Q0KKE9_59340

Q1L7E5_330488

Q1L7E6_330488

37993023_77108

Q84Y60_64040

Q84Y58_64040

Q84Y59_64040

O65130_13612

602902_52853

Sb03g041860.1_4558

Q508G4_4641

Q5XKT6_200642

Q03416_4097

A1XSY2_38733

A1XSX9_38733

186909189_46961

68159393_117978

Q1W5B7_4690

Q9XGK5_3382

Q710D8_3382

Q9XF54_3472

Q5MAR0_3888

Q84M20_13306

Q6QHI2_4513

124484517_208882

Q68BH6_4414

A3QQT2_3435

37993027_3435

A3QQS7_3436

A3QQS6_3436

Q84LC9_41568

Q9LL92_85283

A1XSY4_311263

212656633_3880

212656635_3880

Q309I1_82528

Q309I2_82528

Q309H9_82528

37993029_251264

Q9LL97_3429

Q9LL96_3429

157336091_29760

Q0HA25_29760

SIM4.ALN-TCVV000379_29760

148734381_447319

186909215_46987

Q49P80_78828

Q1ZZ72_171567

A1XSY0_302921

A1XSY1_302921

Q5NU36_77340

Q5NU37_77340

94983067_164110

Q6T4U4_12953

Q6T4V3_257523

Q9XF83_82025

Q9XF84_82025

Q9LL93_118780

Q19R23_127127

Q6T4U6_257524

158563756_3708

158563747_3708

Q0KKF8_59336

Q84L87_186128

186909227_522534

186909225_522534

Q58A79_3311

Q58A74_3311

Q2V8B0_359415

Q4PRG6_3707

A3FJ55_3467

57157437_105750

Q68BI1_105750

Q03378_4151

Q7XAQ2_16752

Q7XAQ3_16752

Q6T4V8_16752

37993035_175201

Q5KTK5_13333

51889428_13333

Q1ZZ76_376443

Q84Y88_13331

Q68BH2_4426

150404768_4378

150404766_4378

Q68BH0_4424

186909179_49683

Q8LT09_82328

Q8LT08_82328

223540527_3988

148734385_13216

Q1L7G8_58286

Q1L7G7_58286

Q1L7F9_82322

Q1L7G0_82322

Q9ZS26_18101

Q84Y79_37489

Q84Y77_37489

Q94FT9_3879

**SEP1**

23304688_3715

Q7X9I7_74645

163929884_55188

Q93X03_3693

evm.TU.supercontig_14.225_3649

Q8GTF1_3712

O82084_3750

Q9ST53_3750

O82694_3750

O82696_3750

212525790_3635

Q3KSZ0_3755

Q948U2_81865

Q6T4V2_218850

Q40969_3347

O04406_3347

Q8S4L4_4081

Q7XBM4_4081

Q8H278_4081

Q7Y040_4081

Q8L5F3_4039

Q6UGQ8_4102

Q9ATF3_4102

Q9ATF1_4102

Q9ATF2_4102

Q7XBK3_4102

Q9ATE6_4102

Q9SEG4_3659

Sb01g042840.1_4558

Q84V74_4577

Q7XBI9_34270

Q40765_3329

Q2TM77_3406

168008840_145481

Q2PNX7_29674

Q9XGJ8_3382

190183765_3369

Q56NI4_3888

Q9LEI1_4513

Q84U54_3747

90657597_228870

90657552_228870

PMADS23_3694

Q39685_3570

188485317_3696

42491276_37657

Q400H5_51953

33309882_51953

Q5K6A1_51953

32478057_74825

Q9SNX0_84618

147765958_29760

147865283_29760

SIM4.ALN-TCVV012585_29760

157356285_29760

Q9ST06_33153

Q6PL58_96049

Q0JRV8_102599

Q9LM09_4097

94983069_164110

LOC_OS03G11614_4530

Q689E4_55190

Q0PM90_118781

115451551_39947

50470536_18101

161158772_4565

Q70JR1_4565

Q1G184_4565

A7BJ59_4565

Q1G181_4565

Q6PL56_38706

Q58A82_3311

Q45VT1_60419

Q3YAG1_60419

Q84JE1_4568

O23767_58167

Q5D723_3467

Q38734_4151

Q7XAQ0_16752

33342042_16752

217071166_3880

148540548_4407

Q84U95_4522

Q84U98_4522

Q9SEG8_4072

A0FIJ3_4072

22091481_79200

9367311_112509

Q3Y4G8_3847

Q4F8B3_3760

A4GVG4_3760

Q6PL61_4498

O64935_71139

52548010_59691

**SEP3**

O64933_71139

Q7XBM3_4081

31747210_4081

Q42464_4081

Q05KK2_55188

Q508G2_4641

evm.TU.supercontig_43.69_3649

LOC_OS09G32948_4530

A2Z2X2_4530

A3BV62_4530

A3AXC8_4530

Q8H6F9_3635

Q6W3F2_3755

Q7XBJ0_34270

Q7XBK2_74823

Q6QHI0_4513

171194265_4513

Q03489_4102

Q7XBK5_4102

O04069_4558

Sb02g029310.1_4558

GRMZM2G159397_P01_4577

Q71UQ7_3329

Q2TM78_13521

Q6PL52_4555

Q84LD3_41568

Q84LD2_41568

Q9XHR9_4096

O65874_3888

Q6GWV5_155132

Q84NB6_3693

PMADS29_3694

647036_3694

Q5D718_3435

Q5D719_3435

A3QQS9_3436

A3QQT0_3436

Q6PL60_38735

42491278_37657

Q5K6A5_51953

Q5K6A3_51953

Q400I4_51953

Q6TH78_13006

Q533S6_34305

Q2EMR9_148910

Q2EMR8_148910

SIM4.ALN-TCVV000587_29760

Q8LLR0_29760

9367315_112509

Q0JRV6_102599

Q5D724_3467

Q9LEP3_3505

Q5GMP6_3847

O82092_49495

A1XDT0_253809

A1XDT1_253809

114309696_3818

218202426_39946

222640923_39947

222629540_39947

115477479_39947

115480039_39947

33304376_39947

115466584_39947

193248835_487795

O04067_3728

Q19R26_127127

Q1G155_4565

Q1G178_4565

Q718F3_4565

Q1G180_4565

Q1G163_4565

Q2TM76_3406

62321064_3702

Q6PL54_4540

Q5PSQ1_4543

Q6PL53_4543

Q84LB9_4232

Q1W2I6_118781

A1XDT4_4686

Q6TYI7_4686

Q38735_4151

Q7XBN5_4151

Q38733_4151

32478005_4151

Q7XAQ1_16752

IMGA_AC144644_9.2_3880

Q6J556_257763

Q6J551_257763

Q5D726_13333

148540546_4407

148540544_4407

148540550_4407

A1IIU4_225117

Q84U96_4522

Q84U99_4522

Q948U3_81865

164507101_3708

Q5D722_3415

172034212_82528

172034216_82528

74053671_51096

151564223_140101

A4GVG3_3760

Q9FST1_18101

194247734_212142

**SHP1**

Q8H280_4081

Q70JR2_4565

161158828_4565

146399991_391288

146160690_94219

189339105_3750

O82698_3750

A2IBV0_3635

Q8H6F8_3635

Q3KSZ2_3755

Q6S6L1_218853

Q6S6L3_218850

Q0QCX0_4100

Q40882_4102

Q43616_4102

Q9SBK3_3659

Q84XW0_3673

Q2ABW9_200642

Q40766_3329

168043223_145481

Q2FC25_117978

Q6RFR1_4690

Q6QX55_4690

Q5MGT5_4690

Q6S6K7_264188

Q9XGK0_3382

Q9XGK4_3382

83991_3218

Q6S6M1_257611

Q6GWV4_155132

287971_3694

Q9ZTW4_3347

2252482_49495

41387778_52518

Q6S6M4_13006

Q533R8_34305

Q58A81_3311

Q8LLQ9_29760

SIM4.ALN-TCVV003413_29760

Q9ST05_33153

148535236_3760

Q2N2U0_3467

87138095_3847

Q40704_4530

Q2QW53_4530

45385946_4530

A2YG00_4530

Q6S6L8_257523

Q5KT55_253809

45387427_39947

75303114_39947

115487796_39947

222636051_39947

108862317_39947

Q6S6K5_3527

Q6S6M9_46970

Q84L86_186128

197690823_375287

4033710_3335

30681253_3702

145332997_3702

Q2V8A9_359415

O23768_58167

evm.TU.supercontig_50.71_3649

Q6S6K8_79245

Q40871_4054

Q9SNY4_82025

Q4TTS9_4641

Q17UR4_3505

Q948U4_81865

19698538_112509

Q2IA03_51096

171194267_4513

Q8RU43_4513

Q9ZTY6_54921

Q5G0F1_46969

**SOC1**

Q0JRV7_102599

Q0JRV9_102599

Q40169_4081

154720967_2711

Q6VAK7_3711

Sb01g049020.1_4558

Q40591_4097

Q09JE1_3750

109627813_118781

evm.TU.supercontig_13.86_3649

Q948U6_81865

Q948U9_81865

218189872_39946

222622003_39947

116794278_3332

116783919_3332

116780851_3332

Q9ATE8_4102

Q9ATE9_4102

Q9ATE3_4102

Q41275_3728

Q9AYR8_3659

Q1G171_4565

Q1G195_4565

Q1G162_4565

Q1G185_4565

195622124_4577

223946823_4577

GRMZM2G171365_P01_4577

219885699_4577

Q58A72_3311

Q58A76_3311

Q58A77_3311

Q40767_3329

Q84LC6_41568

Q7Y137_3693

157101710_3696

Q711P2_4151

Q711P3_4151

Q9XGK6_3382

217075556_3880

IMGA_AC184047_15.2_3880

Q8L6I9_60874

Q52ZP8_3888

Q52ZI9_3888

Q6GWV0_155132

A0EIX6_4120

PTM5_3694

644373_3694

244198_3694

O24488_3347

O24487_3347

30171307_51351

33771690_439823

Q400I3_51953

Q400I0_51953

Q710I0_76084

A1XG54_3847

171194263_4513

145617255_93385

Q7XZQ4_224085

SIM4.ALN-TCVV022785_29760

A1BQ41_29760

Q84LP1_71139

**STK**

Q2WBM3_102599

Q9MBE2_74645

94983056_164110

Q1ZZ71_171567

O65801_49495

Q8VWZ3_3750

33308109_3750

A2ID27_3635

evm.TU.supercontig_471.4_3649

183014295_68875

Q8H281_4081

Q2XUP2_148910

150404774_4378

Q08711_4102

Q43422_3659

Q9ARE9_3659

Sb03g042080.1_4558

GRMZM2G018589_P01_4577

Q710H9_76084

168034724_145481

Q2N2U1_3467

LOC_OS01G66290_4530

Q41195_4151

IMGA_CT868737_5.2_3880

Q689E5_55190

Q689E6_55190

140819_3218

Q1ZZ75_376443

52548104_59691

PMADS21_3694

Q9AXZ1_3708

Q76N61_35883

Q6S6M3_13006

Q533R9_34305

Q75V01_253809

Q0GPY8_3760

Q6S6M2_13258

848999_212142

Q93XH4_29760

157344655_29760

Q56NI2_3888

**SVP**

evm.TU.supercontig_55.32_3649

148912095_126910

148912091_126910

167859853_3329

190183777_3369

190183779_3369

148912105_180580

148912107_180580

LOC_OS06G11330_4530

148912103_126907

PMADS25_3694

224095824_3694

258177_3694

654586_3694

216909_3694

778182_3694

148912085_52874

148912087_52874

148912131_374022

116792789_3332

116788510_3332

148907900_3332

148912089_374025

148912119_304153

148912121_304153

Sb04g033930.2_4558

223944443_4577

GRMZM2G008537_P03_4577

148912143_33120

148912113_300354

157358150_29760

SIM4.ALN-TCVV004900_29760

IMGA_AC135848_22.2_3880

^a^ Number after last underscores indicates species (NCBI taxonomy identifier). For intermolecular analysis, sequences within the same species are combined.
