# Supplementary material for: Conserved and variable correlated mutations in the plant MADS protein network
Source: BMC Genomics. 2010 Oct 28;11:607. doi: 10.1186/1471-2164-11-607 (PMC3017862; doi:10.1186/1471-2164-11-607)
Supplement: Additional file 2 — Input data for intermolecular correlated mutation analysis. This file contains the pairs of interacting proteins used for the intermolecular correlated mutation analysis. [file 1471-2164-11-607-S2.DOC]

Additional File 2. Input data for intermolecular correlated mutation analysis^a^

| **Protein** | **Protein** | **N_pairs_^b^** | **N_species_^b^** |
| --- | --- | --- | --- |
| AG | AGL16 | 32 | 10 |
| AG | FUL | 97 | 44 |
| AGL12 | AGL16 | 35 | 11 |
| AGL12 | AGL21 | 40 | 12 |
| AGL16 | SEP3 | 51 | 12 |
| AGL16 | SHP1 | 37 | 7 |
| AGL19 | AGL21 | 34 | 10 |
| AGL21 | AP1 | 37 | 14 |
| AGL21 | FUL | 36 | 15 |
| AGL21 | SHP1 | 35 | 7 |
| AGL6 | AP1 | 48 | 27 |
| AGL6 | FUL | 75 | 32 |
| AGL6 | SEP1 | 59 | 32 |
| AGL6 | SEP3 | 76 | 33 |
| AGL6 | SOC1 | 45 | 20 |
| AG | SEP1 | 88 | 40 |
| AG | SEP3 | 123 | 47 |
| ANR1 | SOC1 | 47 | 12 |
| AP1 | SEP1 | 89 | 44 |
| AP1 | SEP3 | 114 | 46 |
| AP1 | SOC1 | 64 | 28 |
| AP1 | SVP | 38 | 12 |
| AP3 | PI | 351 | 142 |
| FUL | SEP1 | 137 | 42 |
| FUL | SEP3 | 129 | 42 |
| FUL | SOC1 | 89 | 31 |
| SEP1 | SHP1 | 76 | 27 |
| SEP1 | SOC1 | 98 | 31 |
| SEP1 | STK | 48 | 23 |
| SEP3 | SHP1 | 93 | 27 |
| SEP3 | SOC1 | 87 | 26 |
| SEP3 | STK | 39 | 26 |
| SHP1 | SOC1 | 46 | 18 |
| SOC1 | SVP | 45 | 14 |

^b^ Intermolecular correlated mutations were obtained for pairs of MADS domain proteins shown here, for which at least 30 pairs of sequences of putative orthologs were available. See Supporting Information Table S1 for identifiers.

^a^ N_pairs_, number of different ortholog pairs available; N_species_, number of different species. Note that we do not require 1-to-1 orthology, which means that there can be more than one putative ortholog in a given species.
