# Supplementary material for: Conserved and variable correlated mutations in the plant MADS protein network
Source: BMC Genomics. 2010 Oct 28;11:607. doi: 10.1186/1471-2164-11-607 (PMC3017862; doi:10.1186/1471-2164-11-607)
Supplement: Additional file 3 — Intramolecular correlated mutation results. This files contains the correlated mutation pairs obtained from the intramolecular analysis. [file 1471-2164-11-607-S3.DOC]

**Additional File 3. Intramolecular correlated mutation results**

**AG**

76 N 120 I

76 N 121 Q

79 K 223 R

83 E 90 S

83 E 92 N

83 E 95 T

83 E 101 I

83 E 111 A

83 E 115 Q

83 E 138 K

83 E 142 N

83 E 145 G

83 E 146 R

83 E 159 N

83 E 174 V

83 E 179 D

83 E 181 Q

83 E 194 N

83 E 195 P

83 E 201 P

83 E 203 G

83 E 205 N

114 R 210 M

122 N 156 S

122 N 181 Q

122 N 209 L

124 N 125 R

124 N 141 R

140 L 143 L

140 L 147 L

140 L 148 E

140 L 151 I

147 L 148 E

147 L 150 S

147 L 151 I

147 L 152 T

147 L 156 S

147 L 179 D

147 L 181 Q

147 L 199 L

147 L 209 L

147 L 230 A

161 L 166 I

166 I 168 Y

166 I 171 K

186 K 188 A

186 K 189 E

186 K 190 N

186 K 192 R

186 K 200 M

186 K 206 Y

186 K 220 F

186 K 221 D

186 K 223 R

186 K 224 N

186 K 225 Y

186 K 227 Q

189 E 190 N

189 E 192 R

189 E 206 Y

189 E 220 F

189 E 221 D

189 E 223 R

189 E 227 Q

192 R 206 Y

206 Y 207 E

206 Y 209 L

206 Y 220 F

206 Y 221 D

206 Y 223 R

206 Y 224 N

206 Y 225 Y

206 Y 227 Q

206 Y 228 V

206 Y 229 A

206 Y 238 Y

206 Y 245 D

206 Y 249 L

206 Y 250 Q

212 P 213 P

212 P 215 T

212 P 216 Q

212 P 217 S

212 P 218 Q

212 P 225 Y

212 P 226 F

212 P 233 P

212 P 234 N

212 P 236 H

212 P 237 H

212 P 240 S

212 P 241 A

212 P 250 Q

213 P 215 T

213 P 216 Q

213 P 217 S

213 P 218 Q

213 P 226 F

213 P 233 P

213 P 234 N

213 P 236 H

213 P 237 H

213 P 240 S

213 P 241 A

213 P 250 Q

215 T 216 Q

215 T 217 S

215 T 218 Q

215 T 219 P

215 T 225 Y

215 T 226 F

215 T 233 P

215 T 234 N

215 T 236 H

215 T 237 H

215 T 240 S

215 T 241 A

215 T 250 Q

216 Q 217 S

216 Q 218 Q

216 Q 219 P

216 Q 225 Y

216 Q 226 F

216 Q 233 P

216 Q 234 N

216 Q 236 H

216 Q 237 H

216 Q 240 S

216 Q 241 A

216 Q 250 Q

217 S 234 N

217 S 237 H

217 S 240 S

218 Q 219 P

218 Q 220 F

218 Q 224 N

218 Q 225 Y

218 Q 226 F

218 Q 229 A

218 Q 230 A

218 Q 233 P

218 Q 234 N

218 Q 235 N

218 Q 236 H

218 Q 237 H

218 Q 239 S

218 Q 240 S

218 Q 241 A

218 Q 243 R

218 Q 244 Q

218 Q 245 D

218 Q 246 Q

218 Q 247 T

218 Q 248 A

218 Q 250 Q

219 P 220 F

219 P 224 N

219 P 225 Y

219 P 226 F

219 P 229 A

219 P 230 A

219 P 233 P

219 P 235 N

219 P 236 H

219 P 237 H

219 P 239 S

219 P 240 S

219 P 243 R

219 P 244 Q

219 P 245 D

219 P 246 Q

219 P 247 T

219 P 248 A

219 P 250 Q

221 D 223 R

221 D 224 N

221 D 225 Y

221 D 227 Q

221 D 238 Y

221 D 250 Q

223 R 224 N

223 R 225 Y

223 R 227 Q

227 Q 228 V

227 Q 229 A

227 Q 230 A

227 Q 231 L

227 Q 232 Q

227 Q 233 P

227 Q 235 N

227 Q 236 H

227 Q 237 H

227 Q 238 Y

227 Q 239 S

227 Q 242 G

227 Q 243 R

227 Q 244 Q

227 Q 245 D

227 Q 246 Q

227 Q 247 T

227 Q 248 A

227 Q 249 L

227 Q 250 Q

227 Q 251 L

228 V 229 A

228 V 230 A

228 V 231 L

228 V 232 Q

228 V 234 N

228 V 235 N

228 V 236 H

228 V 237 H

228 V 239 S

228 V 242 G

228 V 243 R

228 V 244 Q

228 V 245 D

228 V 246 Q

228 V 247 T

228 V 248 A

228 V 249 L

228 V 250 Q

228 V 251 L

228 V 252 V

229 A 230 A

229 A 232 Q

229 A 233 P

229 A 234 N

229 A 235 N

229 A 236 H

229 A 237 H

229 A 239 S

229 A 243 R

229 A 244 Q

229 A 245 D

229 A 246 Q

229 A 247 T

229 A 248 A

229 A 249 L

229 A 250 Q

229 A 251 L

229 A 252 V

230 A 231 L

230 A 232 Q

230 A 233 P

230 A 234 N

230 A 235 N

230 A 236 H

230 A 237 H

230 A 239 S

230 A 242 G

230 A 243 R

230 A 244 Q

230 A 245 D

230 A 246 Q

230 A 247 T

230 A 248 A

230 A 249 L

230 A 250 Q

230 A 251 L

230 A 252 V

231 L 232 Q

231 L 235 N

231 L 237 H

231 L 239 S

231 L 242 G

231 L 243 R

231 L 244 Q

231 L 245 D

231 L 246 Q

231 L 247 T

231 L 248 A

231 L 249 L

231 L 250 Q

231 L 251 L

232 Q 234 N

232 Q 235 N

232 Q 236 H

232 Q 237 H

232 Q 239 S

232 Q 242 G

232 Q 243 R

232 Q 244 Q

232 Q 245 D

232 Q 246 Q

232 Q 247 T

232 Q 248 A

232 Q 249 L

232 Q 250 Q

232 Q 251 L

232 Q 252 V

233 P 234 N

233 P 235 N

233 P 236 H

233 P 237 H

233 P 239 S

233 P 240 S

233 P 241 A

233 P 242 G

233 P 243 R

233 P 244 Q

233 P 245 D

233 P 246 Q

233 P 247 T

233 P 248 A

233 P 249 L

233 P 250 Q

233 P 251 L

234 N 235 N

234 N 236 H

234 N 237 H

234 N 239 S

234 N 240 S

234 N 241 A

234 N 242 G

234 N 243 R

234 N 244 Q

234 N 245 D

234 N 247 T

234 N 249 L

234 N 250 Q

234 N 251 L

234 N 252 V

235 N 236 H

235 N 237 H

235 N 239 S

235 N 242 G

235 N 243 R

235 N 244 Q

235 N 245 D

235 N 246 Q

235 N 247 T

235 N 248 A

235 N 249 L

235 N 250 Q

235 N 251 L

235 N 252 V

236 H 237 H

236 H 239 S

236 H 240 S

236 H 241 A

236 H 242 G

236 H 243 R

236 H 244 Q

236 H 245 D

236 H 246 Q

236 H 247 T

236 H 248 A

236 H 249 L

236 H 250 Q

236 H 251 L

237 H 239 S

237 H 240 S

237 H 241 A

237 H 242 G

237 H 243 R

237 H 244 Q

237 H 245 D

237 H 246 Q

237 H 247 T

237 H 248 A

237 H 249 L

237 H 250 Q

237 H 251 L

237 H 252 V

239 S 242 G

239 S 243 R

239 S 244 Q

239 S 245 D

239 S 246 Q

239 S 247 T

239 S 248 A

239 S 249 L

239 S 250 Q

239 S 251 L

239 S 252 V

240 S 241 A

240 S 250 Q

241 A 250 Q

242 G 243 R

242 G 244 Q

242 G 245 D

242 G 246 Q

242 G 247 T

242 G 248 A

242 G 249 L

242 G 250 Q

242 G 251 L

243 R 244 Q

243 R 245 D

243 R 246 Q

243 R 247 T

243 R 248 A

243 R 249 L

243 R 250 Q

243 R 251 L

243 R 252 V

244 Q 245 D

244 Q 246 Q

244 Q 247 T

244 Q 248 A

244 Q 249 L

244 Q 250 Q

244 Q 251 L

244 Q 252 V

245 D 246 Q

245 D 247 T

245 D 248 A

245 D 249 L

245 D 250 Q

245 D 251 L

245 D 252 V

246 Q 247 T

246 Q 248 A

246 Q 249 L

246 Q 250 Q

246 Q 251 L

247 T 248 A

247 T 249 L

247 T 250 Q

247 T 251 L

247 T 252 V

248 A 249 L

248 A 250 Q

248 A 251 L

248 A 252 V

249 L 250 Q

249 L 251 L

249 L 252 V

250 Q 251 L

**AGL6**

30 K 31 A

30 K 32 Y

30 K 34 L

30 K 35 S

31 A 32 Y

31 A 34 L

31 A 35 S

34 L 35 S

108 N 109 L

186 S 187 A

186 S 192 G

186 S 213 T

186 S 214 E

186 S 223 Q

187 A 192 G

187 A 213 T

187 A 214 E

187 A 223 Q

192 G 213 T

192 G 214 E

192 G 223 Q

193 D 200 P

193 D 211 C

193 D 218 Q

211 C 218 Q

211 C 219 I

211 C 229 G

213 T 214 E

214 E 223 Q

218 Q 229 G

218 Q 230 E

218 Q 231 G

218 Q 234 V

218 Q 235 S

218 Q 240 A

218 Q 241 G

218 Q 242 E

236 K 237 S

236 K 238 N

236 K 239 V

236 K 241 G

236 K 242 E

236 K 243 T

236 K 244 N

236 K 245 F

236 K 246 V

236 K 247 Q

236 K 248 G

236 K 249 W

236 K 250 V

237 S 238 N

237 S 239 V

237 S 241 G

237 S 242 E

237 S 243 T

237 S 244 N

237 S 245 F

237 S 246 V

237 S 247 Q

237 S 248 G

237 S 249 W

237 S 250 V

238 N 239 V

238 N 241 G

238 N 242 E

238 N 243 T

238 N 244 N

238 N 245 F

238 N 246 V

238 N 247 Q

238 N 248 G

238 N 249 W

238 N 250 V

239 V 241 G

239 V 242 E

239 V 243 T

239 V 244 N

239 V 245 F

239 V 246 V

239 V 247 Q

239 V 248 G

239 V 249 W

239 V 250 V

241 G 242 E

241 G 243 T

241 G 244 N

241 G 245 F

241 G 246 V

241 G 247 Q

241 G 248 G

241 G 249 W

241 G 250 V

242 E 243 T

242 E 244 N

242 E 245 F

242 E 246 V

242 E 247 Q

242 E 248 G

242 E 249 W

242 E 250 V

243 T 244 N

243 T 245 F

243 T 246 V

243 T 247 Q

243 T 248 G

243 T 249 W

243 T 250 V

244 N 245 F

244 N 246 V

244 N 247 Q

244 N 248 G

244 N 249 W

244 N 250 V

245 F 246 V

245 F 247 Q

245 F 248 G

245 F 249 W

245 F 250 V

246 V 247 Q

246 V 248 G

246 V 249 W

246 V 250 V

247 Q 248 G

247 Q 249 W

247 Q 250 V

248 G 249 W

248 G 250 V

249 W 250 V

**AP1**

36 V 73 S

36 V 80 I

36 V 100 K

36 V 112 L

36 V 146 M

36 V 159 A

36 V 168 S

36 V 37 L

36 V 40 A

36 V 195 P

36 V 211 L

36 V 243 E

37 L 73 S

37 L 74 Y

37 L 80 I

37 L 100 K

37 L 159 A

37 L 168 S

37 L 40 A

37 L 41 E

37 L 195 P

37 L 242 L

40 A 73 S

40 A 74 Y

40 A 80 I

40 A 100 K

40 A 159 A

40 A 41 E

40 A 195 P

40 A 242 L

46 V 157 E

46 V 158 K

46 V 160 I

46 V 162 E

46 V 163 Q

46 V 167 L

46 V 169 K

46 V 173 E

46 V 176 K

59 D 94 Y

62 M 171 I

62 M 173 E

70 E 76 E

72 Y 149 S

73 S 74 Y

73 S 80 I

73 S 100 K

73 S 133 T

73 S 147 Y

73 S 148 E

73 S 159 A

73 S 168 S

73 S 192 H

73 S 195 P

73 S 211 L

73 S 215 P

73 S 225 Y

73 S 232 A

73 S 238 L

73 S 243 E

74 Y 80 I

74 Y 89 N

74 Y 100 K

74 Y 106 R

74 Y 159 A

76 E 89 N

110 H 162 E

155 K 158 K

155 K 160 I

155 K 163 Q

155 K 170 Q

155 K 174 R

155 K 175 E

155 K 181 Q

155 K 212 S

157 E 158 K

157 E 160 I

157 E 163 Q

157 E 167 L

162 E 163 Q

167 L 169 K

167 L 173 E

167 L 176 K

169 K 173 E

171 I 173 E

171 I 174 R

171 I 175 E

171 I 176 K

171 I 181 Q

171 I 185 W

171 I 217 P

171 I 220 N

171 I 236 N

171 I 238 L

171 I 240 L

171 I 244 P

171 I 246 Y

173 E 174 R

173 E 176 K

173 E 185 W

176 K 185 W

176 K 217 P

200 P 201 Q

200 P 202 Q

200 P 203 H

200 P 204 Q

200 P 205 I

200 P 206 Q

200 P 207 H

200 P 208 P

200 P 209 Y

200 P 225 Y

201 Q 202 Q

201 Q 203 H

201 Q 204 Q

201 Q 206 Q

201 Q 207 H

201 Q 208 P

201 Q 209 Y

201 Q 214 Q

201 Q 225 Y

202 Q 203 H

202 Q 204 Q

202 Q 205 I

202 Q 206 Q

202 Q 207 H

202 Q 208 P

202 Q 209 Y

202 Q 225 Y

203 H 204 Q

203 H 205 I

203 H 206 Q

203 H 207 H

203 H 208 P

203 H 209 Y

204 Q 205 I

204 Q 206 Q

204 Q 207 H

204 Q 208 P

204 Q 209 Y

204 Q 225 Y

205 I 206 Q

205 I 207 H

205 I 208 P

205 I 209 Y

206 Q 207 H

206 Q 208 P

206 Q 209 Y

207 H 208 P

217 P 220 N

217 P 240 L

217 P 242 L

217 P 244 P

221 M 222 G

221 M 223 G

221 M 224 L

221 M 225 Y

221 M 226 Q

221 M 227 E

221 M 228 D

221 M 230 P

221 M 231 M

221 M 233 M

221 M 234 R

221 M 235 R

221 M 237 D

221 M 238 L

221 M 240 L

221 M 241 T

221 M 243 E

221 M 244 P

221 M 245 V

221 M 254 A

229 D 230 P

229 D 231 M

229 D 232 A

229 D 233 M

229 D 234 R

229 D 235 R

229 D 236 N

229 D 237 D

229 D 238 L

229 D 239 E

229 D 240 L

229 D 241 T

229 D 242 L

229 D 243 E

229 D 244 P

229 D 245 V

229 D 246 Y

229 D 247 N

229 D 248 C

229 D 249 N

229 D 250 L

229 D 251 G

229 D 252 C

229 D 253 F

229 D 254 A

232 A 233 M

232 A 234 R

232 A 235 R

232 A 236 N

232 A 237 D

232 A 238 L

232 A 239 E

232 A 240 L

232 A 241 T

232 A 242 L

232 A 243 E

232 A 244 P

232 A 245 V

232 A 246 Y

232 A 247 N

232 A 248 C

232 A 249 N

232 A 250 L

232 A 251 G

232 A 252 C

232 A 253 F

232 A 254 A

233 M 234 R

233 M 235 R

233 M 236 N

233 M 237 D

233 M 238 L

233 M 239 E

233 M 240 L

233 M 241 T

233 M 242 L

233 M 243 E

233 M 244 P

233 M 245 V

233 M 246 Y

233 M 247 N

233 M 248 C

233 M 249 N

233 M 250 L

233 M 251 G

233 M 252 C

233 M 253 F

233 M 254 A

234 R 235 R

234 R 236 N

234 R 237 D

234 R 238 L

234 R 239 E

234 R 240 L

234 R 241 T

234 R 242 L

234 R 243 E

234 R 244 P

234 R 245 V

234 R 246 Y

234 R 247 N

234 R 248 C

234 R 249 N

234 R 250 L

234 R 251 G

234 R 252 C

234 R 253 F

234 R 254 A

235 R 236 N

235 R 237 D

235 R 238 L

235 R 239 E

235 R 240 L

235 R 241 T

235 R 243 E

235 R 244 P

235 R 245 V

235 R 246 Y

235 R 247 N

235 R 248 C

235 R 249 N

235 R 250 L

235 R 251 G

235 R 252 C

235 R 253 F

235 R 254 A

236 N 237 D

236 N 238 L

236 N 239 E

236 N 240 L

236 N 241 T

236 N 242 L

236 N 243 E

236 N 244 P

236 N 245 V

236 N 246 Y

236 N 247 N

236 N 248 C

236 N 249 N

236 N 250 L

236 N 251 G

236 N 252 C

236 N 253 F

236 N 254 A

237 D 238 L

237 D 239 E

237 D 240 L

237 D 241 T

237 D 243 E

237 D 244 P

237 D 245 V

237 D 246 Y

237 D 247 N

237 D 248 C

237 D 249 N

237 D 250 L

237 D 251 G

237 D 252 C

237 D 253 F

237 D 254 A

238 L 239 E

238 L 240 L

238 L 241 T

238 L 242 L

238 L 243 E

238 L 244 P

238 L 245 V

238 L 246 Y

238 L 247 N

238 L 248 C

238 L 249 N

238 L 250 L

238 L 251 G

238 L 252 C

238 L 253 F

238 L 254 A

239 E 240 L

239 E 241 T

239 E 242 L

239 E 243 E

239 E 244 P

239 E 245 V

239 E 246 Y

239 E 247 N

239 E 248 C

239 E 249 N

239 E 250 L

239 E 251 G

239 E 252 C

239 E 253 F

239 E 254 A

240 L 241 T

240 L 243 E

240 L 244 P

240 L 245 V

240 L 246 Y

240 L 247 N

240 L 248 C

240 L 249 N

240 L 250 L

240 L 251 G

240 L 252 C

240 L 253 F

240 L 254 A

241 T 242 L

241 T 243 E

241 T 244 P

241 T 245 V

241 T 246 Y

241 T 247 N

241 T 248 C

241 T 249 N

241 T 250 L

241 T 251 G

241 T 252 C

241 T 253 F

241 T 254 A

242 L 243 E

242 L 244 P

243 E 244 P

243 E 245 V

243 E 246 Y

243 E 250 L

243 E 251 G

243 E 252 C

243 E 253 F

243 E 254 A

244 P 245 V

244 P 246 Y

244 P 247 N

244 P 248 C

244 P 249 N

244 P 250 L

244 P 251 G

244 P 252 C

244 P 253 F

244 P 254 A

245 V 250 L

245 V 252 C

245 V 254 A

246 Y 247 N

246 Y 248 C

246 Y 249 N

246 Y 250 L

246 Y 251 G

246 Y 252 C

246 Y 253 F

246 Y 254 A

247 N 248 C

247 N 249 N

247 N 250 L

247 N 251 G

247 N 252 C

247 N 253 F

248 C 249 N

248 C 250 L

248 C 251 G

248 C 252 C

248 C 253 F

249 N 250 L

249 N 251 G

249 N 252 C

249 N 253 F

249 N 254 A

250 L 251 G

250 L 252 C

250 L 253 F

250 L 254 A

251 G 252 C

251 G 253 F

251 G 254 A

252 C 253 F

252 C 254 A

253 F 254 A

**FUL**

35 S 46 V

35 S 49 S

35 S 54 F

35 S 57 S

35 S 76 D

35 S 82 R

35 S 86 Q

35 S 165 N

35 S 177 K

35 S 36 V

35 S 40 A

35 S 41 E

36 V 46 V

36 V 49 S

36 V 54 F

36 V 58 T

36 V 81 G

36 V 86 Q

36 V 165 N

36 V 177 K

36 V 40 A

36 V 41 E

46 V 49 S

46 V 50 K

46 V 52 K

46 V 57 S

51 G 70 D

51 G 81 G

51 G 129 H

51 G 163 H

51 G 188 C

51 G 199 Y

66 L 119 L

130 Q 153 L

135 I 191 S

135 I 201 V

135 I 207 G

135 I 208 F

153 L 156 K

153 L 158 K

153 L 163 H

153 L 172 K

153 L 177 K

153 L 191 S

153 L 193 S

153 L 196 L

153 L 198 Q

153 L 209 V

153 L 222 L

160 L 164 N

207 G 208 F

207 G 210 E

207 G 213 G

207 G 215 E

207 G 216 N

207 G 217 G

207 G 219 A

207 G 220 S

207 G 225 P

207 G 226 N

207 G 228 L

208 F 210 E

208 F 213 G

208 F 215 E

208 F 216 N

208 F 217 G

208 F 219 A

208 F 220 S

208 F 225 P

219 A 220 S

219 A 221 S

219 A 222 L

219 A 223 T

219 A 224 E

219 A 225 P

219 A 226 N

219 A 227 S

219 A 228 L

219 A 229 L

219 A 231 A

219 A 235 R

219 A 236 P

219 A 237 T

219 A 238 T

220 S 221 S

220 S 222 L

220 S 223 T

220 S 224 E

220 S 225 P

220 S 226 N

220 S 227 S

220 S 228 L

220 S 229 L

220 S 231 A

220 S 235 R

220 S 236 P

220 S 237 T

220 S 238 T

221 S 222 L

221 S 223 T

221 S 224 E

221 S 225 P

221 S 226 N

221 S 227 S

221 S 228 L

221 S 229 L

221 S 231 A

221 S 235 R

221 S 236 P

221 S 237 T

221 S 238 T

222 L 223 T

222 L 224 E

222 L 225 P

222 L 226 N

222 L 227 S

222 L 228 L

222 L 229 L

222 L 231 A

222 L 234 L

222 L 235 R

222 L 236 P

222 L 237 T

222 L 238 T

223 T 224 E

223 T 225 P

223 T 226 N

223 T 227 S

223 T 228 L

223 T 229 L

223 T 231 A

223 T 234 L

223 T 235 R

223 T 236 P

223 T 237 T

223 T 238 T

224 E 225 P

224 E 226 N

224 E 227 S

224 E 228 L

224 E 229 L

224 E 231 A

224 E 234 L

224 E 235 R

224 E 236 P

224 E 237 T

224 E 238 T

225 P 226 N

225 P 227 S

225 P 228 L

225 P 229 L

225 P 231 A

225 P 234 L

225 P 235 R

225 P 236 P

225 P 237 T

225 P 238 T

226 N 227 S

226 N 228 L

226 N 229 L

226 N 231 A

226 N 234 L

226 N 235 R

226 N 236 P

226 N 237 T

226 N 238 T

227 S 228 L

227 S 229 L

227 S 231 A

227 S 234 L

227 S 235 R

227 S 236 P

227 S 237 T

227 S 238 T

228 L 229 L

228 L 231 A

228 L 234 L

228 L 235 R

228 L 236 P

228 L 237 T

228 L 238 T

229 L 230 P

229 L 231 A

229 L 233 M

229 L 234 L

229 L 235 R

229 L 236 P

229 L 237 T

229 L 238 T

230 P 232 W

231 A 235 R

231 A 236 P

231 A 237 T

231 A 238 T

234 L 235 R

234 L 236 P

234 L 237 T

234 L 238 T

235 R 236 P

235 R 237 T

235 R 238 T

236 P 237 T

236 P 238 T

237 T 238 T

240 N 241 E

**PI**

36 V 62 D

36 V 121 M

36 V 131 L

36 V 39 D

36 V 40 A

36 V 42 V

36 V 43 A

36 V 44 L

36 V 45 I

36 V 46 I

36 V 47 F

36 V 48 A

36 V 49 S

36 V 51 G

36 V 52 K

36 V 53 M

40 A 62 D

40 A 121 M

40 A 131 L

40 A 42 V

40 A 43 A

40 A 44 L

40 A 45 I

40 A 46 I

40 A 47 F

40 A 48 A

40 A 49 S

40 A 51 G

40 A 52 K

40 A 53 M

42 V 62 D

42 V 121 M

42 V 131 L

42 V 154 E

42 V 43 A

42 V 44 L

42 V 45 I

42 V 46 I

42 V 47 F

42 V 48 A

42 V 49 S

42 V 51 G

42 V 52 K

42 V 53 M

45 I 62 D

45 I 88 N

45 I 121 M

45 I 131 L

45 I 139 M

45 I 154 E

45 I 46 I

45 I 47 F

45 I 48 A

45 I 49 S

45 I 51 G

45 I 52 K

45 I 53 M

46 I 62 D

46 I 68 D

46 I 88 N

46 I 121 M

46 I 131 L

46 I 139 M

46 I 154 E

46 I 47 F

46 I 48 A

46 I 49 S

46 I 51 G

46 I 52 K

46 I 53 M

51 G 62 D

51 G 121 M

51 G 131 L

51 G 52 K

51 G 53 M

52 K 62 D

52 K 121 M

52 K 131 L

52 K 154 E

52 K 53 M

58 C 137 H

58 C 153 A

58 C 202 M

58 C 203 S

58 C 204 L

58 C 205 V

58 C 206 I

110 E 111 D

111 D 112 I

111 D 115 L

111 D 119 N

111 D 123 V

111 D 127 I

114 S 138 Q

119 N 123 V

119 N 127 I

119 N 130 G

119 N 137 H

119 N 140 E

119 N 153 A

119 N 154 E

119 N 155 E

119 N 156 Q

119 N 159 L

119 N 196 N

135 R 138 Q

135 R 140 E

135 R 141 I

135 R 143 I

135 R 146 R

135 R 147 R

135 R 150 K

135 R 157 R

135 R 164 Q

135 R 168 M

135 R 175 R

135 R 179 M

135 R 185 Q

137 H 153 A

137 H 155 E

137 H 156 Q

137 H 185 Q

137 H 186 F

137 H 202 M

137 H 203 S

137 H 204 L

137 H 205 V

137 H 206 I

153 A 154 E

153 A 155 E

181 D 182 H

181 D 184 G

181 D 185 Q

181 D 186 F

181 D 187 G

181 D 189 R

181 D 190 V

181 D 191 Q

181 D 192 P

181 D 193 I

181 D 194 Q

181 D 195 P

181 D 196 N

181 D 197 L

181 D 198 Q

181 D 199 E

181 D 200 K

182 H 183 D

182 H 184 G

182 H 185 Q

182 H 186 F

182 H 187 G

182 H 188 Y

182 H 189 R

182 H 190 V

182 H 191 Q

182 H 193 I

182 H 194 Q

182 H 198 Q

182 H 199 E

182 H 200 K

183 D 184 G

183 D 185 Q

183 D 186 F

183 D 187 G

183 D 193 I

183 D 199 E

183 D 200 K

184 G 185 Q

184 G 186 F

184 G 187 G

184 G 188 Y

184 G 190 V

184 G 193 I

184 G 194 Q

184 G 198 Q

184 G 199 E

184 G 200 K

185 Q 186 F

185 Q 187 G

185 Q 188 Y

185 Q 189 R

185 Q 190 V

185 Q 191 Q

185 Q 192 P

185 Q 193 I

185 Q 194 Q

185 Q 195 P

185 Q 197 L

185 Q 198 Q

185 Q 199 E

185 Q 200 K

186 F 187 G

186 F 188 Y

186 F 189 R

186 F 190 V

186 F 191 Q

186 F 193 I

186 F 194 Q

186 F 198 Q

186 F 199 E

186 F 200 K

187 G 188 Y

187 G 189 R

187 G 190 V

187 G 191 Q

187 G 192 P

187 G 193 I

187 G 194 Q

187 G 195 P

187 G 196 N

187 G 197 L

187 G 198 Q

187 G 199 E

187 G 200 K

188 Y 190 V

188 Y 191 Q

188 Y 192 P

188 Y 193 I

188 Y 194 Q

188 Y 195 P

188 Y 196 N

188 Y 197 L

188 Y 198 Q

188 Y 199 E

188 Y 200 K

189 R 190 V

189 R 191 Q

189 R 192 P

189 R 193 I

189 R 194 Q

189 R 195 P

189 R 196 N

189 R 197 L

189 R 198 Q

189 R 199 E

189 R 200 K

190 V 191 Q

190 V 192 P

190 V 193 I

190 V 194 Q

190 V 195 P

190 V 196 N

190 V 197 L

190 V 198 Q

190 V 199 E

190 V 200 K

191 Q 192 P

191 Q 194 Q

191 Q 195 P

191 Q 196 N

192 P 194 Q

192 P 195 P

192 P 196 N

193 I 194 Q

193 I 198 Q

193 I 199 E

193 I 200 K

194 Q 195 P

194 Q 196 N

194 Q 197 L

194 Q 198 Q

194 Q 199 E

194 Q 200 K

195 P 196 N

198 Q 199 E

198 Q 200 K

199 E 200 K

202 M 203 S

202 M 204 L

202 M 205 V

202 M 206 I

203 S 204 L

203 S 205 V

203 S 206 I

204 L 205 V

204 L 206 I

205 V 206 I

**SEP1**

25 N 160 L

25 N 171 L

25 N 27 L

25 N 32 Y

25 N 34 L

25 N 36 V

27 L 160 L

27 L 171 L

27 L 32 Y

27 L 34 L

27 L 36 V

58 S 94 Y

58 S 95 L

58 S 108 Q

58 S 136 K

58 S 140 S

58 S 146 M

58 S 170 K

66 L 93 E

66 L 96 K

66 L 97 L

66 L 98 K

66 L 100 R

66 L 102 E

66 L 105 Q

66 L 108 Q

66 L 115 D

66 L 123 E

66 L 130 Q

66 L 132 D

66 L 134 S

66 L 135 L

93 E 97 L

93 E 98 K

93 E 102 E

93 E 114 E

93 E 115 D

93 E 123 E

93 E 130 Q

93 E 134 S

97 L 98 K

97 L 102 E

97 L 134 S

98 K 102 E

98 K 105 Q

98 K 108 Q

98 K 115 D

98 K 123 E

98 K 130 Q

98 K 134 S

102 E 108 Q

102 E 115 D

102 E 123 E

102 E 130 Q

102 E 134 S

105 Q 123 E

105 Q 134 S

130 Q 131 L

130 Q 134 S

130 Q 135 L

138 V 139 R

144 Q 148 D

160 L 161 L

160 L 168 A

160 L 171 L

160 L 177 V

160 L 199 A

160 L 204 L

175 I 176 G

176 G 179 S

176 G 190 Q

176 G 192 V

176 G 194 Y

176 G 195 A

176 G 198 Q

176 G 209 E

176 G 212 P

176 G 224 S

176 G 225 E

176 G 234 Q

176 G 235 A

218 Y 244 G

230 T 231 T

230 T 232 Q

230 T 234 Q

230 T 235 A

230 T 236 Q

230 T 237 P

230 T 238 G

230 T 239 N

230 T 240 G

230 T 241 Y

230 T 242 I

230 T 243 P

230 T 244 G

230 T 245 W

230 T 246 M

231 T 232 Q

231 T 233 A

231 T 234 Q

231 T 235 A

231 T 236 Q

231 T 237 P

231 T 238 G

231 T 239 N

231 T 240 G

231 T 241 Y

231 T 242 I

231 T 243 P

231 T 244 G

231 T 245 W

231 T 246 M

232 Q 233 A

232 Q 234 Q

232 Q 235 A

232 Q 236 Q

232 Q 238 G

232 Q 240 G

232 Q 241 Y

232 Q 242 I

232 Q 243 P

232 Q 244 G

232 Q 246 M

233 A 235 A

233 A 236 Q

233 A 238 G

233 A 239 N

233 A 240 G

233 A 241 Y

233 A 242 I

233 A 243 P

233 A 244 G

234 Q 235 A

234 Q 236 Q

234 Q 237 P

234 Q 238 G

234 Q 239 N

234 Q 240 G

234 Q 241 Y

234 Q 242 I

234 Q 243 P

234 Q 244 G

234 Q 245 W

234 Q 246 M

235 A 236 Q

235 A 237 P

235 A 238 G

235 A 239 N

235 A 240 G

235 A 241 Y

235 A 242 I

235 A 243 P

235 A 244 G

235 A 245 W

235 A 246 M

236 Q 237 P

236 Q 238 G

236 Q 239 N

236 Q 240 G

236 Q 241 Y

236 Q 242 I

236 Q 243 P

236 Q 244 G

236 Q 245 W

236 Q 246 M

237 P 238 G

237 P 239 N

237 P 240 G

237 P 241 Y

237 P 242 I

237 P 243 P

237 P 244 G

237 P 246 M

238 G 239 N

238 G 240 G

238 G 241 Y

238 G 242 I

238 G 243 P

238 G 244 G

238 G 245 W

238 G 246 M

239 N 240 G

239 N 241 Y

239 N 242 I

239 N 243 P

239 N 244 G

239 N 245 W

239 N 246 M

240 G 241 Y

240 G 242 I

240 G 243 P

240 G 244 G

240 G 245 W

240 G 246 M

241 Y 242 I

241 Y 243 P

241 Y 244 G

241 Y 246 M

242 I 243 P

242 I 244 G

242 I 245 W

242 I 246 M

243 P 244 G

243 P 245 W

243 P 246 M

244 G 245 W

244 G 246 M

**SEP3**

31 A 32 Y

31 A 34 L

31 A 36 V

31 A 41 E

31 A 55 E

31 A 66 L

34 L 36 V

34 L 41 E

34 L 55 E

34 L 58 S

34 L 64 R

34 L 68 R

34 L 101 K

34 L 103 R

36 V 41 E

36 V 55 E

36 V 58 S

36 V 64 R

36 V 74 Y

36 V 99 K

36 V 103 R

36 V 177 G

36 V 230 A

36 V 235 N

41 E 43 A

41 E 50 R

41 E 53 L

41 E 55 E

41 E 58 S

41 E 62 M

41 E 65 T

41 E 66 L

41 E 68 R

41 E 74 Y

41 E 97 Y

41 E 99 K

41 E 101 K

41 E 103 R

41 E 107 L

41 E 108 Q

41 E 111 Q

41 E 244 Y

41 E 245 D

41 E 246 T

41 E 247 N

50 R 55 E

50 R 58 S

50 R 62 M

50 R 65 T

50 R 66 L

50 R 68 R

50 R 74 Y

50 R 97 Y

50 R 98 L

50 R 99 K

50 R 101 K

50 R 103 R

50 R 107 L

50 R 108 Q

50 R 111 Q

50 R 113 N

50 R 244 Y

50 R 245 D

50 R 246 T

50 R 247 N

55 E 58 S

55 E 62 M

55 E 65 T

55 E 66 L

55 E 68 R

55 E 74 Y

55 E 97 Y

55 E 99 K

55 E 101 K

55 E 103 R

55 E 107 L

55 E 108 Q

55 E 111 Q

55 E 193 H

55 E 244 Y

55 E 245 D

55 E 246 T

55 E 247 N

58 S 62 M

58 S 66 L

58 S 68 R

58 S 71 K

58 S 74 Y

58 S 85 E

58 S 97 Y

58 S 99 K

58 S 101 K

58 S 103 R

58 S 107 L

58 S 108 Q

58 S 111 Q

58 S 193 H

58 S 244 Y

58 S 245 D

58 S 246 T

58 S 247 N

62 M 66 L

62 M 68 R

62 M 74 Y

62 M 97 Y

62 M 99 K

62 M 101 K

62 M 103 R

62 M 107 L

62 M 108 Q

62 M 244 Y

62 M 245 D

62 M 246 T

62 M 247 N

65 T 68 R

65 T 74 Y

65 T 97 Y

65 T 99 K

65 T 101 K

65 T 103 R

68 R 70 Q

68 R 71 K

68 R 74 Y

68 R 97 Y

68 R 98 L

68 R 99 K

68 R 101 K

68 R 103 R

68 R 107 L

68 R 108 Q

68 R 111 Q

68 R 120 G

68 R 193 H

68 R 199 H

68 R 244 Y

68 R 245 D

68 R 246 T

68 R 247 N

88 A 244 Y

88 A 245 D

88 A 246 T

97 Y 98 L

97 Y 99 K

97 Y 101 K

97 Y 103 R

97 Y 107 L

97 Y 108 Q

97 Y 111 Q

97 Y 113 N

97 Y 244 Y

97 Y 245 D

97 Y 246 T

97 Y 247 N

100 L 105 D

100 L 107 L

101 K 103 R

101 K 107 L

101 K 108 Q

101 K 111 Q

101 K 244 Y

101 K 245 D

101 K 246 T

101 K 247 N

103 R 107 L

103 R 108 Q

103 R 111 Q

103 R 128 E

103 R 244 Y

103 R 245 D

103 R 246 T

103 R 247 N

107 L 108 Q

107 L 111 Q

107 L 244 Y

107 L 245 D

107 L 246 T

107 L 247 N

108 Q 111 Q

108 Q 135 D

108 Q 244 Y

108 Q 245 D

108 Q 246 T

108 Q 247 N

125 K 135 D

150 L 151 D

150 L 157 Q

150 L 166 T

150 L 170 L

150 L 172 L

150 L 173 R

150 L 193 H

150 L 196 R

150 L 197 H

150 L 208 F

150 L 220 I

150 L 227 D

151 D 157 Q

151 D 166 T

151 D 170 L

151 D 172 L

151 D 173 R

151 D 193 H

151 D 196 R

151 D 197 H

151 D 227 D

160 E 167 N

163 L 169 T

163 L 175 A

163 L 178 Y

163 L 180 M

163 L 182 L

163 L 183 Q

163 L 187 N

163 L 188 Q

163 L 189 E

163 L 198 H

163 L 200 Q

163 L 205 S

163 L 207 A

163 L 213 E

163 L 219 Q

163 L 223 Q

170 L 173 R

170 L 193 H

170 L 196 R

170 L 197 H

170 L 227 D

193 H 196 R

193 H 197 H

193 H 227 D

193 H 244 Y

193 H 245 D

193 H 246 T

193 H 247 N

196 R 197 H

196 R 227 D

197 H 227 D

211 P 213 E

211 P 214 C

211 P 215 E

211 P 216 P

211 P 217 I

211 P 219 Q

211 P 220 I

211 P 221 G

211 P 222 Y

211 P 223 Q

211 P 225 Q

211 P 226 Q

211 P 227 D

211 P 230 A

211 P 231 G

211 P 232 P

211 P 233 S

211 P 235 N

211 P 236 N

214 C 215 E

214 C 217 I

214 C 219 Q

214 C 220 I

214 C 222 Y

214 C 223 Q

214 C 224 G

214 C 225 Q

214 C 226 Q

214 C 228 G

214 C 229 G

214 C 230 A

214 C 231 G

214 C 232 P

214 C 233 S

214 C 235 N

214 C 236 N

214 C 237 Y

214 C 239 L

214 C 240 G

216 P 217 I

216 P 218 L

216 P 220 I

216 P 221 G

217 I 218 L

217 I 219 Q

217 I 220 I

217 I 221 G

217 I 222 Y

217 I 223 Q

217 I 224 G

217 I 225 Q

217 I 226 Q

217 I 227 D

217 I 228 G

217 I 229 G

217 I 230 A

217 I 231 G

217 I 232 P

217 I 234 V

217 I 235 N

217 I 236 N

217 I 237 Y

217 I 239 L

217 I 240 G

217 I 242 L

218 L 220 I

218 L 221 G

219 Q 220 I

219 Q 223 Q

219 Q 224 G

219 Q 225 Q

219 Q 226 Q

219 Q 227 D

219 Q 228 G

219 Q 229 G

219 Q 230 A

219 Q 231 G

219 Q 232 P

219 Q 234 V

219 Q 235 N

219 Q 236 N

219 Q 237 Y

219 Q 239 L

219 Q 240 G

219 Q 242 L

220 I 221 G

220 I 222 Y

220 I 223 Q

220 I 224 G

220 I 225 Q

220 I 226 Q

220 I 227 D

220 I 228 G

220 I 229 G

220 I 230 A

220 I 231 G

220 I 232 P

220 I 234 V

220 I 235 N

220 I 236 N

220 I 237 Y

220 I 239 L

220 I 240 G

220 I 242 L

223 Q 224 G

223 Q 225 Q

223 Q 226 Q

223 Q 227 D

223 Q 228 G

223 Q 229 G

223 Q 230 A

223 Q 231 G

223 Q 232 P

223 Q 233 S

223 Q 234 V

223 Q 235 N

223 Q 236 N

223 Q 237 Y

223 Q 239 L

223 Q 240 G

223 Q 242 L

224 G 225 Q

224 G 226 Q

224 G 227 D

224 G 228 G

224 G 229 G

224 G 230 A

224 G 231 G

224 G 232 P

224 G 234 V

224 G 235 N

224 G 236 N

224 G 237 Y

224 G 239 L

224 G 240 G

224 G 242 L

225 Q 226 Q

225 Q 227 D

225 Q 228 G

225 Q 229 G

225 Q 230 A

225 Q 231 G

225 Q 232 P

225 Q 233 S

225 Q 234 V

225 Q 235 N

225 Q 236 N

225 Q 237 Y

225 Q 239 L

225 Q 240 G

225 Q 242 L

226 Q 227 D

226 Q 228 G

226 Q 229 G

226 Q 230 A

226 Q 231 G

226 Q 232 P

226 Q 233 S

226 Q 234 V

226 Q 235 N

226 Q 236 N

226 Q 237 Y

226 Q 239 L

226 Q 240 G

226 Q 242 L

227 D 228 G

227 D 229 G

227 D 230 A

227 D 233 S

227 D 234 V

227 D 235 N

227 D 236 N

227 D 237 Y

227 D 239 L

227 D 240 G

227 D 242 L

228 G 229 G

228 G 230 A

228 G 231 G

228 G 232 P

228 G 234 V

228 G 235 N

228 G 236 N

228 G 237 Y

228 G 239 L

228 G 240 G

228 G 242 L

229 G 230 A

229 G 231 G

229 G 232 P

229 G 234 V

229 G 235 N

229 G 236 N

229 G 237 Y

229 G 239 L

229 G 240 G

229 G 242 L

230 A 231 G

230 A 232 P

230 A 233 S

230 A 234 V

230 A 235 N

230 A 236 N

230 A 237 Y

230 A 239 L

230 A 240 G

230 A 242 L

231 G 232 P

231 G 233 S

231 G 235 N

231 G 236 N

231 G 237 Y

231 G 239 L

231 G 240 G

231 G 242 L

232 P 233 S

232 P 235 N

232 P 236 N

232 P 237 Y

232 P 239 L

232 P 240 G

232 P 242 L

233 S 235 N

233 S 236 N

233 S 237 Y

233 S 238 M

233 S 239 L

233 S 240 G

234 V 235 N

234 V 236 N

234 V 237 Y

234 V 239 L

234 V 240 G

234 V 242 L

235 N 236 N

235 N 237 Y

235 N 239 L

235 N 240 G

235 N 242 L

236 N 237 Y

236 N 239 L

236 N 240 G

236 N 242 L

237 Y 239 L

237 Y 240 G

237 Y 242 L

239 L 240 G

239 L 242 L

240 G 242 L

244 Y 245 D

244 Y 246 T

244 Y 247 N

245 D 246 T

245 D 247 N

246 T 247 N

**SHP1**

2 E 3 G

2 E 6 S

2 E 8 D

2 E 11 S

3 G 6 S

3 G 7 H

3 G 8 D

3 G 11 S

3 G 13 K

5 S 6 S

5 S 7 H

5 S 8 D

5 S 9 A

6 S 7 H

6 S 8 D

6 S 11 S

6 S 13 K

7 H 8 D

8 D 9 A

8 D 11 S

8 D 12 S

8 D 13 K

9 A 12 S

11 S 13 K

51 V 56 E

114 Q 115 I

114 Q 118 I

191 L 192 N

226 P 227 V

226 P 228 N

226 P 229 L

226 P 230 L

226 P 231 E

226 P 232 P

226 P 233 N

226 P 234 Q

226 P 235 Q

226 P 236 F

226 P 237 S

226 P 238 G

226 P 239 Q

226 P 241 Q

226 P 242 P

226 P 243 P

226 P 244 L

226 P 245 Q

226 P 246 L

231 E 232 P

231 E 233 N

231 E 234 Q

231 E 235 Q

231 E 236 F

231 E 237 S

231 E 238 G

231 E 239 Q

231 E 241 Q

231 E 242 P

231 E 243 P

231 E 244 L

231 E 245 Q

231 E 246 L

232 P 233 N

232 P 234 Q

232 P 235 Q

232 P 236 F

232 P 237 S

232 P 238 G

232 P 239 Q

232 P 240 D

232 P 241 Q

232 P 242 P

232 P 243 P

232 P 244 L

232 P 245 Q

232 P 246 L

232 P 247 V

234 Q 235 Q

234 Q 236 F

234 Q 237 S

234 Q 238 G

234 Q 239 Q

234 Q 241 Q

234 Q 242 P

234 Q 243 P

234 Q 244 L

234 Q 245 Q

234 Q 246 L

234 Q 247 V

235 Q 236 F

235 Q 237 S

235 Q 238 G

235 Q 239 Q

235 Q 241 Q

235 Q 242 P

235 Q 243 P

235 Q 244 L

235 Q 245 Q

235 Q 246 L

235 Q 247 V

236 F 237 S

236 F 238 G

236 F 239 Q

236 F 240 D

236 F 241 Q

236 F 242 P

236 F 243 P

236 F 244 L

236 F 245 Q

236 F 246 L

237 S 238 G

237 S 239 Q

237 S 240 D

237 S 241 Q

237 S 242 P

237 S 243 P

237 S 244 L

237 S 245 Q

237 S 246 L

238 G 239 Q

238 G 240 D

238 G 241 Q

238 G 242 P

238 G 243 P

238 G 244 L

238 G 245 Q

238 G 246 L

239 Q 240 D

239 Q 241 Q

239 Q 242 P

239 Q 243 P

239 Q 244 L

239 Q 245 Q

239 Q 246 L

240 D 241 Q

240 D 242 P

240 D 243 P

240 D 244 L

240 D 246 L

241 Q 242 P

241 Q 243 P

241 Q 244 L

241 Q 245 Q

241 Q 246 L

241 Q 247 V

242 P 243 P

242 P 244 L

242 P 245 Q

242 P 246 L

243 P 244 L

243 P 245 Q

243 P 246 L

244 L 245 Q

244 L 246 L

245 Q 246 L

245 Q 247 V

**SOC1**

34 L 35 S

34 L 39 D

34 L 45 I

39 D 42 V

39 D 45 I

42 V 45 I

49 P 90 K

67 R 68 Y

67 R 110 L

67 R 159 L

67 R 162 E

68 Y 110 L

68 Y 159 L

155 K 156 E

163 N 166 L

199 V 200 E

199 V 201 T

199 V 202 Q

199 V 203 L

199 V 204 F

199 V 205 I

199 V 206 G

199 V 207 L

199 V 208 P

199 V 209 C

199 V 210 S

199 V 211 S

199 V 212 R

201 T 202 Q

201 T 203 L

201 T 204 F

201 T 205 I

201 T 206 G

201 T 207 L

201 T 208 P

201 T 209 C

201 T 210 S

201 T 211 S

201 T 212 R

202 Q 204 F

202 Q 205 I

202 Q 206 G

202 Q 207 L

202 Q 208 P

202 Q 209 C

202 Q 210 S

202 Q 211 S

202 Q 212 R

202 Q 213 K

203 L 206 G

203 L 208 P

204 F 205 I

204 F 206 G

204 F 207 L

204 F 208 P

204 F 209 C

204 F 210 S

204 F 211 S

204 F 212 R

204 F 213 K

205 I 206 G

205 I 207 L

205 I 208 P

205 I 209 C

205 I 210 S

205 I 211 S

205 I 212 R

206 G 207 L

206 G 208 P

206 G 209 C

206 G 210 S

206 G 211 S

206 G 212 R

206 G 213 K

207 L 208 P

207 L 209 C

207 L 210 S

207 L 211 S

207 L 212 R

207 L 213 K

208 P 209 C

208 P 210 S

208 P 211 S

208 P 212 R

208 P 213 K

209 C 210 S

209 C 211 S

209 C 212 R

210 S 211 S

210 S 212 R

210 S 213 K

211 S 212 R

211 S 213 K

212 R 213 K

**STK**

87 A 89 Y

87 A 93 S

87 A 106 S

87 A 121 K

87 A 132 K

87 A 142 H

87 A 147 V

87 A 152 A

87 A 161 N

153 Q 154 K

153 Q 158 E

153 Q 161 N

153 Q 163 N

153 Q 166 L

153 Q 169 K

153 Q 170 V

153 Q 171 A

153 Q 172 E

153 Q 174 E

153 Q 175 R

153 Q 198 R

153 Q 199 N

153 Q 205 I

153 Q 218 Y

153 Q 226 L

153 Q 227 H

154 K 158 E

154 K 161 N

154 K 163 N

154 K 166 L

154 K 169 K

154 K 170 V

154 K 171 A

154 K 172 E

154 K 174 E

154 K 175 R

154 K 198 R

154 K 199 N

154 K 205 I

154 K 218 Y

154 K 226 L

154 K 227 H

163 N 166 L

163 N 169 K

163 N 170 V

163 N 171 A

163 N 172 E

163 N 174 E

163 N 175 R

163 N 198 R

163 N 199 N

163 N 200 Y

163 N 205 I

163 N 218 Y

163 N 224 K

163 N 226 L

163 N 227 H

166 L 169 K

166 L 170 V

166 L 171 A

166 L 172 E

166 L 174 E

166 L 175 R

166 L 198 R

166 L 199 N

166 L 205 I

166 L 218 Y

166 L 226 L

166 L 227 H

169 K 170 V

169 K 171 A

169 K 172 E

169 K 174 E

169 K 175 R

169 K 198 R

169 K 199 N

169 K 200 Y

169 K 205 I

169 K 218 Y

169 K 224 K

169 K 226 L

169 K 227 H

170 V 171 A

170 V 172 E

170 V 174 E

170 V 175 R

170 V 198 R

170 V 199 N

170 V 200 Y

170 V 205 I

170 V 218 Y

170 V 224 K

170 V 226 L

170 V 227 H

172 E 174 E

172 E 175 R

172 E 178 Q

172 E 198 R

172 E 199 N

172 E 200 Y

172 E 205 I

172 E 218 Y

172 E 224 K

172 E 226 L

172 E 227 H

175 R 177 Q

175 R 178 Q

175 R 179 H

175 R 181 H

175 R 183 M

175 R 198 R

175 R 199 N

175 R 200 Y

175 R 205 I

175 R 218 Y

175 R 219 S

175 R 223 K

175 R 224 K

175 R 226 L

175 R 227 H

202 A 203 H

202 A 204 S

202 A 205 I

202 A 206 M

202 A 207 T

202 A 208 A

202 A 215 G

202 A 216 G

202 A 217 S

202 A 218 Y

202 A 219 S

202 A 220 D

202 A 221 P

202 A 222 D

202 A 223 K

202 A 224 K

202 A 225 I

202 A 227 H

203 H 204 S

203 H 205 I

203 H 206 M

203 H 207 T

203 H 208 A

203 H 214 N

203 H 215 G

203 H 216 G

203 H 217 S

203 H 218 Y

203 H 219 S

203 H 220 D

203 H 221 P

203 H 222 D

203 H 223 K

203 H 224 K

203 H 225 I

203 H 227 H

204 S 205 I

204 S 206 M

204 S 207 T

204 S 208 A

204 S 215 G

204 S 216 G

204 S 217 S

204 S 218 Y

204 S 219 S

204 S 220 D

204 S 221 P

204 S 222 D

204 S 223 K

204 S 224 K

204 S 225 I

204 S 227 H

205 I 206 M

205 I 207 T

205 I 208 A

205 I 215 G

205 I 216 G

205 I 217 S

205 I 218 Y

205 I 219 S

205 I 220 D

205 I 221 P

205 I 222 D

205 I 223 K

205 I 224 K

205 I 225 I

205 I 226 L

205 I 227 H

205 I 228 L

206 M 207 T

206 M 208 A

206 M 214 N

206 M 215 G

206 M 216 G

206 M 217 S

206 M 218 Y

206 M 219 S

206 M 220 D

206 M 221 P

206 M 222 D

206 M 223 K

206 M 224 K

206 M 225 I

206 M 227 H

207 T 208 A

207 T 215 G

207 T 216 G

207 T 217 S

207 T 218 Y

207 T 219 S

207 T 220 D

207 T 221 P

207 T 222 D

207 T 223 K

207 T 224 K

207 T 225 I

207 T 227 H

208 A 215 G

208 A 216 G

208 A 217 S

208 A 218 Y

208 A 219 S

208 A 220 D

208 A 221 P

208 A 222 D

208 A 223 K

208 A 224 K

208 A 225 I

208 A 227 H

215 G 216 G

215 G 217 S

215 G 218 Y

215 G 219 S

215 G 220 D

215 G 221 P

215 G 222 D

215 G 223 K

215 G 224 K

215 G 225 I

215 G 227 H

216 G 217 S

216 G 218 Y

216 G 219 S

216 G 220 D

216 G 221 P

216 G 222 D

216 G 223 K

216 G 224 K

216 G 225 I

216 G 227 H

217 S 218 Y

217 S 219 S

217 S 220 D

217 S 221 P

217 S 222 D

217 S 223 K

217 S 224 K

217 S 225 I

217 S 227 H

218 Y 219 S

218 Y 220 D

218 Y 221 P

218 Y 222 D

218 Y 223 K

218 Y 224 K

218 Y 225 I

218 Y 226 L

218 Y 227 H

218 Y 228 L

219 S 220 D

219 S 221 P

219 S 222 D

219 S 223 K

219 S 224 K

219 S 225 I

219 S 227 H

220 D 221 P

220 D 222 D

220 D 223 K

220 D 224 K

220 D 225 I

220 D 227 H

221 P 222 D

221 P 223 K

221 P 224 K

221 P 225 I

221 P 227 H

222 D 223 K

222 D 224 K

222 D 225 I

222 D 227 H

223 K 224 K

223 K 225 I

223 K 227 H

224 K 225 I

224 K 226 L

224 K 227 H

224 K 228 L

225 I 227 H

226 L 227 H

227 H 228 L

**SVP**

11 D 97 S

11 D 110 M

11 D 135 T

11 D 12 N

11 D 71 Q

11 D 72 S

11 D 81 P

15 A 120 I

15 A 136 R

15 A 144 K

15 A 149 I

15 A 89 E

159 L 161 D

159 L 162 E

159 L 163 N

159 L 166 L

162 E 163 N

163 N 166 L

185 S 186 T

185 S 187 I

185 S 188 N

185 S 206 H

185 S 207 T

186 T 187 I

186 T 188 N

186 T 206 H

186 T 207 T

186 T 209 R

187 I 188 N

187 I 189 V

187 I 190 H

187 I 206 H

187 I 207 T

187 I 209 R

188 N 189 V

188 N 190 H

188 N 206 H

188 N 207 T

188 N 209 R

189 V 206 H

189 V 209 R

190 H 206 H

190 H 207 T

203 M 204 F

206 H 207 T

206 H 209 R

207 T 209 R

For ease of reference of sequence position numbers in this and subsequent tables, we here provide also the sequences of the relevant MADS domain proteins:

>AG

TAYQSELGGDSSPLRKSGRGKIEIKRIENTTNRQVTFCKRRNGLLKKAYELSVLCDAEVA

LIVFSSRGRLYEYSNNSVKGTIERYKKAISDNSNTGSVAEINAQYYQQESAKLRQQIISI

QNSNRQLMGETIGSMSPKELRNLEGRLERSITRIRSKKNELLFSEIDYMQKREVDLHNDN

QILRAKIAENERNNPSISLMPGGSNYEQLMPPPQTQSQPFDSRNYFQVAALQPNNHHYSS

AGRQDQTALQLV

>AGL6

GRGRVEMKRIENKINRQVTFSKRRNGLLKKAYELSVLCDAEVALIIFSSRGKLYEFGSVG

IESTIERYNRCYNCSLSNNKPEETTQSWCQEVTKLKSKYESLVRTNRNLLGEDLGEMGVK

ELQALERQLEAALTATRQRKTQVMMEEMEDLRKKERQLGDINKQLKIKFETEGHAFKTFQ

DLWANSAASVAGDPNNSEFPVEPSHPNVLDCNTEPFLQIGFQQHYYVQGEGSSVSKSNVA

GETNFVQGWVL

>AP1

GRGRVQLKRIENKINRQVTFSKRRAGLLKKAHEISVLCDAEVALVVFSHKGKLFEYSTDS

CMEKILERYERYSYAERQLIAPESDVNTNWSMEYNRLKAKIELLERNQRHYLGEDLQAMS

PKELQNLEQQLDTALKHIRTRKNQLMYESINELQKKEKAIQEQNSMLSKQIKEREKILRA

QQEQWDQQNQGHNMPPPLPPQQHQIQHPYMLSHQPSPFLNMGGLYQEDDPMAMRRNDLEL

TLEPVYNCNLGCFAA

>AP3

ARGKIQIKRIENQTNRQVTYSKRRNGLFKKAHELTVLCDARVSIIMFSSSNKLHEYISPN

TTTKEIVDLYQTISDVDVWATQYERMQETKRKLLETNRNLRTQIKQRLGECLDELDIQEL

RRLEDEMENTFKLVRERKFKSLGNQIETTKKKNKSQQDIQKNLIHELELRAEDPHYGLVD

NGGDYDSVLGYQIEGSRAYALRFHQNHHHYYPNHGLHAPSASDIITFHLLE

>FUL

GRGRVQLKRIENKINRQVTFSKRRSGLLKKAHEISVLCDAEVALIVFSSKGKLFEYSTDS

CMERILERYDRYLYSDKQLVGRDVSQSENWVLEHAKLKARVEVLEKNKRNFMGEDLDSLS

LKELQSLEHQLDAAIKSIRSRKNQAMFESISALQKKDKALQDHNNSLLKKIKEREKKTGQ

QEGQLVQCSNSSSVLLPQYCVTSSRDGFVERVGGENGGASSLTEPNSLLPAWMLRPTTTN

E

>PI

GRGKIEIKRIENANNRVVTFSKRRNGLVKKAKEITVLCDAKVALIIFASNGKMIDYCCPS

MDLGAMLDQYQKLSGKKLWDAKHENLSNEIDRIKKENDSLQLELRHLKGEDIQSLNLKNL

MAVEHAIEHGLDKVRDHQMEILISKRRNEKMMAEEQRQLTFQLQQQEMAIASNARGMMMR

DHDGQFGYRVQPIQPNLQEKIMSLVID

>SEP1

GRGRVELKRIENKINRQVTFAKRRNGLLKKAYELSVLCDAEVALIIFSNRGKLYEFCSSS

NMLKTLDRYQKCSYGSIEVNNKPAKELENSYREYLKLKGRYENLQRQQRNLLGEDLGPLN

SKELEQLERQLDGSLKQVRSIKTQYMLDQLSDLQNKEQMLLETNRALAMKLDDMIGVRSH

HMGGGGGWEGGEQNVTYAHHQAQSQGLYQPLECNPTLQMGYDNPVCSEQITATTQAQAQQ

GNGYIPGWML

>SEP3

GRGRVELKRIENKINRQVTFAKRRNGLLKKAYELSVLCDAEVALIIFSNRGKLYEFCSSS

SMLRTLERYQKCNYGAPEPNVPSREALAVELSSQQEYLKLKERYDALQRTQRNLLGEDLG

PLSTKELESLERQLDSSLKQIRALRTQFMLDQLNDLQSKERMLTETNKTLRLRLADGYQM

PLQLNPNQEEVDHYGRHHHQQQQHSQAFFQPLECEPILQIGYQGQQDGMGAGPSVNNYML

GWLPYDTNSI

>SHP1

EEGGSSHDAESSKKLGRGKIEIKRIENTTNRQVTFCKRRNGLLKKAYELSVLCDAEVALV

IFSTRGRLYEYANNSVRGTIERYKKACSDAVNPPSVTEANTQYYQQEASKLRRQIRDIQN

SNRHIVGESLGSLNFKELKNLEGRLEKGISRVRSKKNELLVAEIEYMQKREMELQHNNMY

LRAKIAEGARLNPDQQESSVIQGTTVYESGVSSHDQSQHYNRNYIPVNLLEPNQQFSGQD

QPPLQLV

>SOC1

VRGKTQMKRIENATSRQVTFSKRRNGLLKKAFELSVLCDAEVSLIIFSPKGKLYEFASSN

MQDTIDRYLRHTKDRVSTKPVSEENMQHLKYEAANMMKKIEQLEASKRKLLGEGIGTCSI

EELQQIEQQLEKSVKCIRARKTQVFKEQIEQLKQKEKALAAENEKLSEKWGSHESEVWSN

KNQESTGRGDEESSPSSEVETQLFIGLPCSSRK

>STK

GRGKIEIKRIENSTNRQVTFCKRRNGLLKKAYELSVLCDAEVALIVFSTRGRLYEYANNN

IRSTIERYKKACSDSTNTSTVQEINAAYYQQESAKLRQQIQTIQNSNRNLMGDSLSSLSV

KELKQVENRLEKAISRIRSKKHELLLVEIENAQKREIELDNENIYLRTKVAEVERYQQHH

HQMVSGSEINAIEALASRNYFAHSIMTAGSGSGNGGSYSDPDKKILHLG

>SVP

AREKIQIRKIDNATARQVTFSKRRRGLFKKAEELSVLCDADVALIIFSSTGKLFEFCSSS

MKEVLERHNLQSKNLEKLDQPSLELQLVENSDHARMSKEIADKSHRLRQMRGEELQGLDI

EELQQLEKALETGLTRVIETKSDKIMSEISELQKKGMQLMDENKRLRQQVCVLPSLLITN

PFLLSTINVHTPKFNPQLSTTHMFDHTVR
