# Supplementary material for: Conserved and variable correlated mutations in the plant MADS protein network
Source: BMC Genomics. 2010 Oct 28;11:607. doi: 10.1186/1471-2164-11-607 (PMC3017862; doi:10.1186/1471-2164-11-607)
Supplement: Additional file 4 — Intermolecular correlated mutation results. This files contains the correlated mutation pairs obtained from the intermolecular analysis. [file 1471-2164-11-607-S4.DOC]

**Additional File 4. Intermolecular correlated mutation results**

**AG_AGL16**

98 V 9 R

98 V 42 V

98 V 5 I

98 V 7 I

101 I 5 I

**AGL12_AGL16**

116 S 9 R

116 S 42 V

116 S 53 L

116 S 5 I

116 S 7 I

**AGL12_AGL21**

175 L 123 N

**AGL21_FUL**

62 K 109 R

62 K 27 L

62 K 67 E

63 S 109 R

86 K 109 R

110 M 109 R

110 M 167 L

114 L 27 L

116 G 109 R

116 G 167 L

119 V 109 R

119 V 155 K

119 V 167 L

162 N 109 R

**AGL6_AP1**

27 L 36 V

27 L 41 E

37 L 36 V

37 L 41 E

78 N 197 P

78 N 198 L

78 N 199 P

78 N 200 P

78 N 201 Q

78 N 202 Q

78 N 203 H

78 N 28 L

138 Q 76 E

138 Q 89 N

138 Q 100 K

138 Q 122 K

138 Q 149 S

138 Q 74 Y

214 E 197 P

214 E 198 L

214 E 199 P

217 L 76 E

217 L 122 K

**AGL6_FUL**

49 S 158 K

138 Q 125 Q

154 K 158 K

**AGL6_SEP1**

28 L 96 K

28 L 102 E

28 L 108 Q

28 L 109 R

28 L 112 L

28 L 123 E

31 A 93 E

31 A 97 L

31 A 98 K

31 A 102 E

31 A 108 Q

31 A 109 R

31 A 123 E

31 A 134 S

31 A 66 L

31 A 68 R

31 A 69 Y

32 Y 108 Q

32 Y 109 R

32 Y 123 E

32 Y 134 S

49 S 46 I

49 S 55 E

50 R 102 E

51 G 112 L

51 G 65 T

53 L 91 Y

53 L 94 Y

53 L 134 S

55 E 164 N

55 E 215 Q

58 S 68 R

63 S 91 Y

65 I 164 N

65 I 215 Q

104 R 140 S

115 G 140 S

117 M 102 E

117 M 112 L

117 M 140 S

117 M 170 K

117 M 58 S

120 K 91 Y

120 K 125 E

120 K 140 S

123 Q 65 T

133 L 140 S

133 L 170 K

146 E 140 S

154 K 46 I

154 K 55 E

172 E 181 H

183 W 91 Y

**AGL6_SEP3**

51 G 125 K

51 G 152 Q

89 C 34 L

89 C 35 S

89 C 41 E

89 C 42 V

89 C 44 L

89 C 45 I

89 C 46 I

89 C 53 L

96 K 5 V

96 K 9 R

96 K 10 I

123 Q 125 K

123 Q 152 Q

147 E 93 S

147 E 2 R

147 E 5 V

147 E 9 R

147 E 10 I

147 E 11 E

147 E 15 N

147 E 21 A

**AGL6_SOC1**

28 L 62 Q

28 L 65 I

28 L 34 L

28 L 36 V

28 L 37 L

28 L 39 D

28 L 40 A

28 L 41 E

28 L 45 I

28 L 46 I

28 L 52 K

28 L 55 E

28 L 56 F

30 K 34 L

30 K 36 V

30 K 37 L

30 K 39 D

30 K 40 A

30 K 41 E

31 A 62 Q

31 A 65 I

31 A 34 L

31 A 36 V

31 A 37 L

31 A 39 D

31 A 40 A

31 A 41 E

31 A 45 I

31 A 46 I

31 A 52 K

31 A 55 E

31 A 56 F

34 L 34 L

34 L 36 V

34 L 37 L

34 L 39 D

34 L 40 A

34 L 41 E

36 V 34 L

36 V 36 V

36 V 37 L

36 V 39 D

42 V 34 L

42 V 36 V

42 V 37 L

42 V 39 D

42 V 41 E

42 V 52 K

43 A 58 S

43 A 113 E

43 A 152 L

43 A 38 C

46 I 62 Q

46 I 65 I

46 I 42 V

46 I 44 L

46 I 45 I

46 I 46 I

46 I 47 F

46 I 51 G

46 I 52 K

46 I 54 Y

46 I 55 E

46 I 56 F

49 S 61 M

49 S 72 T

49 S 75 R

49 S 92 E

49 S 106 S

49 S 117 T

50 R 62 Q

50 R 22 K

50 R 24 R

50 R 52 K

51 G 38 C

53 L 62 Q

53 L 65 I

53 L 67 R

53 L 90 K

53 L 107 K

53 L 109 K

53 L 45 I

53 L 46 I

53 L 50 K

53 L 51 G

53 L 52 K

53 L 54 Y

53 L 56 F

54 Y 62 Q

54 Y 65 I

54 Y 42 V

54 Y 44 L

54 Y 45 I

54 Y 46 I

54 Y 47 F

54 Y 51 G

54 Y 52 K

54 Y 54 Y

54 Y 55 E

54 Y 56 F

56 F 62 Q

56 F 65 I

56 F 42 V

56 F 44 L

56 F 45 I

56 F 46 I

56 F 47 F

56 F 51 G

56 F 52 K

56 F 54 Y

56 F 55 E

56 F 56 F

58 S 62 Q

58 S 64 T

58 S 65 I

58 S 67 R

58 S 83 E

58 S 43 S

58 S 45 I

58 S 46 I

58 S 52 K

58 S 56 F

104 R 62 Q

104 R 22 K

104 R 24 R

104 R 52 K

109 L 22 K

109 L 24 R

169 F 38 C

186 S 124 Q

186 S 212 R

187 A 124 Q

187 A 212 R

193 D 124 Q

193 D 212 R

194 P 96 M

218 Q 96 M

218 Q 35 S

223 Q 124 Q

244 N 72 T

244 N 96 M

244 N 98 K

244 N 121 E

244 N 144 V

244 N 147 E

244 N 173 H

244 N 174 E

244 N 188 R

244 N 35 S

248 G 65 I

248 G 67 R

248 G 38 C

248 G 45 I

248 G 46 I

**AG_SEP1**

13 P 15 N

13 P 54 Y

13 P 57 C

93 S 65 T

93 S 67 D

96 G 97 L

96 G 98 K

96 G 102 E

96 G 134 S

96 G 93 E

96 G 94 Y

120 I 140 S

120 I 170 K

120 I 58 S

121 Q 45 I

122 N 170 K

122 N 58 S

177 H 15 N

177 H 54 Y

189 E 91 Y

**AG_SEP3**

108 Q 101 K

112 K 101 K

121 Q 39 D

121 Q 41 E

121 Q 42 V

121 Q 44 L

149 R 101 K

**ANR1_SOC1**

65 I 73 K

65 I 93 A

65 I 101 E

65 I 109 K

65 I 130 L

65 I 132 K

66 I 101 E

66 I 109 K

68 R 73 K

68 R 99 K

68 R 109 K

68 R 50 K

116 S 62 Q

116 S 65 I

116 S 7 M

116 S 27 L

116 S 36 V

116 S 41 E

116 S 42 V

116 S 45 I

116 S 46 I

116 S 54 Y

116 S 55 E

116 S 58 S

223 E 76 V

223 E 77 S

223 E 86 M

223 E 96 M

223 E 114 G

223 E 150 E

223 E 204 F

223 E 25 N

223 E 57 A

226 I 76 V

226 I 77 S

226 I 96 M

226 I 114 G

226 I 150 E

226 I 25 N

227 R 76 V

227 R 77 S

227 R 96 M

227 R 114 G

227 R 150 E

227 R 25 N

229 G 76 V

229 G 77 S

229 G 86 M

229 G 96 M

229 G 114 G

229 G 150 E

229 G 204 F

229 G 25 N

**AP1_SEP1**

42 V 139 R

42 V 153 L

55 E 102 E

55 E 108 Q

55 E 112 L

61 C 212 P

61 C 213 T

61 C 60 S

72 Y 93 E

72 Y 94 Y

72 Y 98 K

72 Y 102 E

72 Y 105 Q

72 Y 134 S

72 Y 66 L

72 Y 68 R

72 Y 69 Y

74 Y 65 T

76 E 65 T

84 S 82 K

84 S 99 G

84 S 106 R

84 S 120 N

84 S 121 S

84 S 129 R

84 S 159 M

84 S 168 A

84 S 169 M

84 S 179 S

84 S 189 E

84 S 190 Q

84 S 192 V

84 S 4 R

84 S 196 H

84 S 211 N

84 S 213 T

84 S 220 N

84 S 230 T

84 S 238 G

84 S 75 G

93 E 58 S

111 Y 80 N

111 Y 120 N

111 Y 136 K

111 Y 145 Y

111 Y 155 N

111 Y 191 N

111 Y 194 Y

111 Y 197 H

111 Y 205 Y

111 Y 212 P

111 Y 213 T

111 Y 231 T

111 Y 241 Y

111 Y 15 N

111 Y 57 C

132 D 145 Y

148 E 96 K

149 S 94 Y

149 S 95 L

149 S 102 E

149 S 105 Q

149 S 134 S

149 S 68 R

149 S 69 Y

215 P 166 A

250 L 58 S

**AP1_SEP3**

200 P 54 Y

**AP1_SOC1**

71 R 130 L

71 R 131 E

135 L 113 E

135 L 127 E

135 L 138 R

135 L 152 L

154 Q 38 C

**FUL_SEP1**

65 I 162 E

65 I 164 N

125 Q 91 Y

135 I 83 P

135 I 99 G

135 I 145 Y

135 I 166 A

135 I 173 D

135 I 191 N

135 I 212 P

144 Q 162 E

144 Q 164 N

144 Q 215 Q

149 S 162 E

204 S 83 P

204 S 99 G

**FUL_SOC1**

55 E 101 E

55 E 104 E

97 L 109 K

97 L 127 E

97 L 130 L

156 K 101 E

156 K 42 V

156 K 50 K

205 R 88 H

205 R 170 W

205 R 178 W

205 R 187 G

**SEP1_SHP1**

4 R 78 G

4 R 82 R

4 R 87 C

4 R 143 G

4 R 150 S

4 R 157 N

4 R 180 Y

4 R 208 E

4 R 17 R

4 R 30 N

36 V 78 G

36 V 82 R

36 V 143 G

36 V 150 S

36 V 157 N

36 V 180 Y

36 V 208 E

36 V 17 R

36 V 30 N

41 E 102 Q

41 E 139 K

42 V 102 Q

49 N 78 G

49 N 82 R

49 N 87 C

49 N 103 Y

49 N 143 G

49 N 150 S

49 N 157 N

49 N 175 Q

49 N 180 Y

49 N 208 E

49 N 17 R

49 N 30 N

58 S 104 Y

58 S 172 M

60 S 78 G

60 S 87 C

74 Y 78 G

75 G 78 G

75 G 82 R

75 G 87 C

75 G 143 G

75 G 150 S

75 G 157 N

75 G 175 Q

75 G 180 Y

75 G 183 A

75 G 208 E

75 G 17 R

75 G 30 N

77 I 78 G

77 I 82 R

77 I 87 C

77 I 103 Y

77 I 143 G

77 I 147 K

77 I 180 Y

77 I 208 E

77 I 17 R

77 I 30 N

78 E 78 G

81 N 78 G

81 N 82 R

81 N 143 G

81 N 157 N

81 N 180 Y

81 N 17 R

89 N 78 G

89 N 87 C

89 N 208 E

89 N 30 N

90 S 104 Y

90 S 172 M

90 S 207 Y

90 S 30 N

91 Y 65 R

91 Y 69 Y

91 Y 100 N

91 Y 102 Q

91 Y 120 N

91 Y 123 R

91 Y 139 K

92 R 78 G

92 R 82 R

92 R 180 Y

92 R 17 R

92 R 30 N

94 Y 65 R

94 Y 100 N

94 Y 144 R

95 L 65 R

95 L 69 Y

101 Y 78 G

101 Y 82 R

101 Y 143 G

101 Y 150 S

101 Y 157 N

101 Y 180 Y

101 Y 208 E

101 Y 17 R

102 E 69 Y

102 E 100 N

102 E 120 N

102 E 123 R

102 E 139 K

102 E 148 G

111 L 78 G

111 L 82 R

111 L 87 C

111 L 143 G

111 L 150 S

111 L 157 N

111 L 180 Y

111 L 208 E

111 L 17 R

111 L 30 N

112 L 102 Q

112 L 24 R

129 R 78 G

129 R 82 R

129 R 87 C

129 R 143 G

129 R 150 S

129 R 157 N

129 R 180 Y

129 R 17 R

129 R 30 N

131 L 78 G

131 L 82 R

131 L 87 C

131 L 124 H

131 L 143 G

131 L 150 S

131 L 157 N

131 L 180 Y

131 L 17 R

131 L 30 N

133 G 78 G

133 G 82 R

133 G 87 C

133 G 103 Y

133 G 143 G

133 G 147 K

133 G 150 S

133 G 157 N

133 G 180 Y

133 G 208 E

133 G 17 R

133 G 30 N

134 S 65 R

134 S 69 Y

135 L 65 R

136 K 78 G

136 K 82 R

136 K 87 C

136 K 147 K

136 K 175 Q

136 K 180 Y

136 K 208 E

136 K 17 R

136 K 30 N

143 T 78 G

143 T 82 R

143 T 143 G

143 T 150 S

143 T 157 N

143 T 180 Y

143 T 17 R

143 T 30 N

156 K 47 Y

156 K 29 T

156 K 40 N

158 Q 78 G

158 Q 82 R

158 Q 87 C

158 Q 143 G

158 Q 157 N

158 Q 180 Y

158 Q 208 E

158 Q 17 R

158 Q 30 N

163 T 78 G

163 T 82 R

163 T 87 C

163 T 103 Y

163 T 143 G

163 T 150 S

163 T 157 N

163 T 180 Y

163 T 208 E

163 T 17 R

163 T 30 N

165 R 78 G

165 R 82 R

165 R 87 C

165 R 103 Y

165 R 143 G

165 R 150 S

165 R 157 N

165 R 180 Y

165 R 208 E

165 R 17 R

165 R 30 N

170 K 102 Q

177 V 78 G

177 V 82 R

177 V 143 G

177 V 150 S

177 V 157 N

177 V 180 Y

177 V 17 R

177 V 30 N

179 S 78 G

179 S 82 R

179 S 87 C

179 S 143 G

179 S 150 S

179 S 157 N

179 S 180 Y

179 S 208 E

179 S 17 R

179 S 30 N

180 H 78 G

180 H 82 R

180 H 87 C

180 H 143 G

180 H 157 N

180 H 180 Y

180 H 208 E

180 H 17 R

180 H 30 N

181 H 78 G

181 H 82 R

181 H 87 C

181 H 143 G

181 H 150 S

181 H 157 N

181 H 180 Y

181 H 208 E

181 H 17 R

181 H 30 N

185 W 47 Y

185 W 24 R

185 W 40 N

187 G 78 G

187 G 82 R

187 G 87 C

187 G 143 G

187 G 150 S

187 G 157 N

187 G 180 Y

187 G 208 E

187 G 17 R

187 G 30 N

188 G 78 G

188 G 82 R

188 G 143 G

188 G 150 S

188 G 157 N

188 G 180 Y

188 G 208 E

188 G 17 R

188 G 30 N

189 E 78 G

189 E 82 R

189 E 87 C

189 E 143 G

189 E 150 S

189 E 157 N

189 E 180 Y

189 E 208 E

189 E 17 R

189 E 30 N

192 V 78 G

192 V 82 R

192 V 87 C

192 V 143 G

192 V 150 S

192 V 157 N

192 V 180 Y

192 V 208 E

192 V 17 R

192 V 30 N

193 T 78 G

193 T 82 R

193 T 180 Y

193 T 17 R

193 T 30 N

195 A 78 G

195 A 82 R

195 A 87 C

195 A 143 G

195 A 150 S

195 A 157 N

195 A 180 Y

195 A 208 E

195 A 17 R

195 A 30 N

196 H 78 G

196 H 82 R

200 Q 78 G

200 Q 82 R

200 Q 87 C

200 Q 143 G

200 Q 150 S

200 Q 157 N

200 Q 180 Y

200 Q 208 E

200 Q 17 R

202 Q 78 G

202 Q 82 R

202 Q 87 C

202 Q 157 N

202 Q 180 Y

202 Q 208 E

202 Q 17 R

202 Q 30 N

203 G 78 G

203 G 82 R

203 G 87 C

203 G 143 G

203 G 150 S

203 G 157 N

203 G 180 Y

203 G 208 E

203 G 17 R

203 G 30 N

207 P 78 G

207 P 82 R

207 P 87 C

207 P 180 Y

207 P 208 E

207 P 17 R

207 P 30 N

218 Y 78 G

218 Y 82 R

218 Y 17 R

218 Y 30 N

220 N 78 G

220 N 82 R

220 N 87 C

220 N 143 G

220 N 150 S

220 N 157 N

220 N 180 Y

220 N 208 E

220 N 17 R

220 N 30 N

221 P 87 C

221 P 104 Y

221 P 172 M

221 P 208 E

221 P 30 N

223 C 78 G

223 C 82 R

223 C 17 R

230 T 78 G

230 T 87 C

230 T 103 Y

230 T 104 Y

230 T 147 K

230 T 172 M

230 T 208 E

230 T 30 N

235 A 78 G

237 P 78 G

237 P 82 R

237 P 143 G

237 P 157 N

237 P 180 Y

237 P 17 R

237 P 30 N

238 G 78 G

238 G 82 R

238 G 143 G

238 G 150 S

238 G 157 N

238 G 180 Y

238 G 17 R

238 G 30 N

243 P 78 G

243 P 82 R

243 P 87 C

243 P 143 G

243 P 150 S

243 P 157 N

243 P 180 Y

243 P 208 E

243 P 17 R

243 P 30 N

245 W 78 G

245 W 17 R

**SEP1_SOC1**

45 I 67 R

45 I 68 Y

45 I 110 L

45 I 123 L

45 I 159 L

45 I 162 E

45 I 163 N

66 L 62 Q

66 L 45 I

66 L 46 I

66 L 48 S

66 L 55 E

67 D 45 I

68 R 46 I

68 R 48 S

69 Y 62 Q

69 Y 42 V

69 Y 45 I

69 Y 46 I

69 Y 48 S

69 Y 51 G

69 Y 54 Y

69 Y 55 E

69 Y 56 F

91 Y 62 Q

91 Y 42 V

91 Y 45 I

91 Y 46 I

91 Y 47 F

91 Y 48 S

91 Y 51 G

91 Y 54 Y

91 Y 55 E

91 Y 56 F

93 E 62 Q

93 E 45 I

93 E 46 I

93 E 48 S

93 E 54 Y

93 E 56 F

97 L 62 Q

97 L 65 I

97 L 45 I

97 L 46 I

97 L 55 E

98 K 62 Q

98 K 42 V

98 K 45 I

98 K 46 I

98 K 48 S

98 K 51 G

98 K 54 Y

98 K 55 E

98 K 56 F

102 E 62 Q

102 E 42 V

102 E 45 I

102 E 46 I

102 E 48 S

102 E 51 G

102 E 54 Y

102 E 55 E

102 E 56 F

105 Q 62 Q

105 Q 45 I

105 Q 46 I

105 Q 48 S

105 Q 51 G

105 Q 55 E

105 Q 56 F

108 Q 62 Q

108 Q 34 L

108 Q 36 V

108 Q 37 L

108 Q 39 D

108 Q 45 I

108 Q 46 I

108 Q 51 G

108 Q 55 E

115 D 62 Q

115 D 45 I

115 D 46 I

115 D 48 S

134 S 62 Q

134 S 42 V

134 S 45 I

134 S 46 I

134 S 48 S

134 S 51 G

134 S 55 E

134 S 56 F

135 L 62 Q

135 L 42 V

135 L 45 I

135 L 46 I

135 L 47 F

135 L 48 S

135 L 51 G

135 L 54 Y

135 L 55 E

135 L 56 F

**SEP1_STK**

14 I 94 A

14 I 110 L

14 I 114 S

14 I 117 S

14 I 160 D

14 I 188 E

14 I 191 A

14 I 192 I

14 I 193 E

14 I 197 S

14 I 209 G

15 N 94 A

15 N 110 L

15 N 114 S

15 N 117 S

15 N 160 D

15 N 188 E

15 N 191 A

72 C 13 S

74 Y 81 V

74 Y 89 Y

74 Y 132 K

91 Y 83 E

91 Y 89 Y

91 Y 121 K

91 Y 132 K

93 E 81 V

94 Y 132 K

94 Y 134 I

94 Y 152 A

96 K 171 A

97 L 84 I

97 L 152 A

97 L 165 Y

97 L 171 A

98 K 152 A

108 Q 99 Q

108 Q 25 N

125 E 83 E

125 E 89 Y

125 E 94 A

125 E 114 S

125 E 121 K

125 E 132 K

131 L 99 Q

131 L 13 S

131 L 25 N

131 L 50 R

131 L 61 I

134 S 152 A

135 L 152 A

139 R 80 T

139 R 81 V

143 T 82 Q

143 T 98 Q

143 T 110 L

143 T 114 S

143 T 117 S

143 T 13 S

144 Q 99 Q

144 Q 110 L

144 Q 13 S

144 Q 25 N

146 M 98 Q

146 M 101 Q

146 M 110 L

146 M 160 D

146 M 226 L

149 Q 81 V

149 Q 98 Q

149 Q 101 Q

149 Q 103 I

149 Q 226 L

150 L 98 Q

150 L 101 Q

150 L 110 L

150 L 160 D

150 L 226 L

150 L 13 S

156 K 98 Q

156 K 101 Q

156 K 226 L

173 D 114 S

173 D 188 E

185 W 83 E

185 W 89 Y

185 W 121 K

185 W 132 K

215 Q 94 A

215 Q 114 S

215 Q 117 S

215 Q 160 D

215 Q 188 E

215 Q 191 A

215 Q 203 H

215 Q 204 S

244 G 89 Y

244 G 94 A

244 G 114 S

244 G 117 S

244 G 118 L

244 G 160 D

**SEP3_SHP1**

216 P 4 G

216 P 5 S

216 P 6 S

216 P 7 H

218 L 3 G

218 L 4 G

218 L 5 S

218 L 6 S

218 L 7 H

218 L 9 A

218 L 10 E

**SEP3_SOC1**

65 T 159 L

65 T 166 L

97 Y 159 L

97 Y 166 L

98 L 130 L

98 L 159 L

98 L 166 L

111 Q 130 L

111 Q 159 L

111 Q 166 L

113 N 159 L

113 N 166 L

114 L 58 S

115 L 38 C

125 K 38 C

156 L 67 R

156 L 68 Y

156 L 110 L

156 L 123 L

156 L 43 S

158 S 114 G

158 S 145 F

158 S 25 N

**SEP3_STK**

31 A 1 G

31 A 5 I

31 A 6 E

31 A 8 K

33 E 1 G

33 E 5 I

33 E 6 E

33 E 8 K

34 L 6 E

35 S 1 G

35 S 5 I

35 S 6 E

35 S 8 K

36 V 71 A

36 V 106 S

36 V 120 V

36 V 122 E

36 V 187 S

36 V 210 S

41 E 52 R

41 E 161 N

50 R 52 R

101 K 83 E

101 K 140 K

101 K 161 N

111 Q 52 R

111 Q 161 N

113 N 52 R

113 N 161 N

114 L 1 G

114 L 5 I

114 L 6 E

114 L 8 K

115 L 52 R

115 L 161 N

117 E 52 R

117 E 161 N

125 K 52 R

125 K 161 N

128 E 83 E

128 E 140 K

128 E 161 N

135 D 83 E

135 D 140 K

135 D 161 N

144 L 87 A

150 L 52 R

157 Q 52 R

160 E 52 R

160 E 83 E

160 E 140 K

160 E 161 N

174 L 144 L

**SHP1_SOC1**

22 I 7 M

22 I 9 R

22 I 11 E

22 I 18 V

22 I 24 R

22 I 27 L

27 N 7 M

27 N 9 R

27 N 11 E

27 N 18 V

27 N 24 R

27 N 27 L

33 V 7 M

33 V 9 R

33 V 11 E

33 V 18 V

33 V 24 R

36 C 7 M

36 C 9 R

36 C 11 E

36 C 18 V

36 C 24 R

40 N 7 M

40 N 9 R

40 N 11 E

40 N 18 V

40 N 24 R

42 L 7 M

42 L 9 R

42 L 11 E

42 L 18 V

42 L 24 R

42 L 27 L

47 Y 7 M

47 Y 9 R

47 Y 11 E

47 Y 18 V

47 Y 24 R

50 S 7 M

50 S 9 R

50 S 11 E

50 S 18 V

50 S 24 R

106 Q 34 L

107 E 113 E

107 E 58 S

110 K 113 E

110 K 58 S

111 L 113 E

111 L 58 S

114 Q 113 E

114 Q 58 S

115 I 113 E

115 I 58 S

118 I 113 E

118 I 58 S

120 N 9 R

120 N 11 E

120 N 18 V

120 N 24 R

120 N 27 L

120 N 34 L

159 L 67 R

159 L 110 L

159 L 159 L

168 Q 61 M

168 Q 67 R

168 Q 110 L

168 Q 123 L

168 Q 162 E

168 Q 163 N

**SOC1_SVP**

102 Q 139 E

102 Q 142 S

102 Q 36 V

102 Q 88 V

Here and in all tables in Supporting Information, residue numbers refer to positions of amino acids in the respective sequences.
