# Supplementary material for: Conserved and variable correlated mutations in the plant MADS protein network
Source: BMC Genomics. 2010 Oct 28;11:607. doi: 10.1186/1471-2164-11-607 (PMC3017862; doi:10.1186/1471-2164-11-607)
Supplement: Additional file 5 — Short distance enrichment significance. This file contains an analysis of the statistical significance of the observed short distance enrichment for the intermolecular correlated mutations. [file 1471-2164-11-607-S5.DOC]

**Additional File 5. Short distance enrichment significance**

To further analyze the significance of the observed short distance enrichment for the intermolecular correlated mutations, we applied a resampling analysis were the original pairs of multiple sequence alignments were shuffled such that pairs of sequences no longer occurred in the same species but were randomly assigned to each other; this was performed 1000 times. As this is computationally rather costly, we limited randomization trials to a (large) subset of the interacting pairs.

When comparing with the MADS structural data, the interacting pairs can be divided in the following subsets: (I) those that had more than 30 sequences (our starting criterion) and for which at least one correlated mutation position was predicted in the MADS domain (for which structural information is available); (II) those that had more than 30 sequences and for which no correlated mutation position was predicted in the MADS domain; and (III), those with less than 30 sequences. Note that the latter were left out in all results presented in the paper, but were only included here for the sake of comparison.

For the protein pairs in set I, the randomization trial indicated in most cases that the observed enrichment of residues at short distances was significant. Specifically, for SEP1-SOC1 (98 sequences), SEP1-SHP1 (76) AGL6-AP1 (48), AGL6-SEP1 (59) and ANR1-SOC1 (47), in at most 11 out of 1000 randomly shuffled pairs of alignments the fraction of residues at short distances among the predicted correlated mutation positions was the same or higher than that in the original analysis. For the two cases in set I with the fewest sequences, AGL6-SOC1 (45 sequences) and AGL21-FUL (36), the result was close to significance (85, respectively 61 out of 1000 random trials had the same or higher fraction).

For the protein pairs in set II, in most cases (16 out of 23) in at least 950 out of 1000 random trials there were no correlated mutation pairs predicted in the MADS domain. In addition, for 19 out of 23 pairs, in those trials where there were correlated mutation positions predicted in the MADS domain, the distance enrichment was lower than the average enrichment in the original dataset (55%); on average, this distance enrichment in those randomizations was 38% (+/- 19%), which is similar to the percentage observed in the crystal structure (39%).

One protein pair with less than 30 sequences was tested (SEP1-SVP, 24 sequences). This was the only pair for which the overrepresentation at short distances was clearly not significant (207 out of 1000 random trials had the same or higher enrichment for residue pairs at short distances).
