# Supplementary material for: Conserved and variable correlated mutations in the plant MADS protein network
Source: BMC Genomics. 2010 Oct 28;11:607. doi: 10.1186/1471-2164-11-607 (PMC3017862; doi:10.1186/1471-2164-11-607)
Supplement: Additional file 6 — Correlated mutations that have consistent overlap with predicted interaction motifs. This file contains correlated mutations that have consistent overlap with predicted interaction motifs and are strong candidates to be important residues for protein-protein interactions of the MADS domain proteins. [file 1471-2164-11-607-S6.DOC]

Additional File 6 Correlated mutations that have consistent overlap with predicted interaction motifs

| **Protein1** | **Protein2** | **Residue1** | **AminoAcid1** | **Residue2** | **AminoAcid2** |  |
| --- | --- | --- | --- | --- | --- | --- |
| AP1 | SEP1 | 61 | C | 60 | S |  |
| AGL6 | SEP1 | 117 | M | 58 | S |  |
| AGL6 | SEP1 | 117 | M | 112 | L |  |
| AGL21 | FUL | 62 | K | 67 | E |  |
| SEP1 | SHP1 | 158 | Q | 143 | G |  |
| SEP1 | SHP1 | 131 | L | 124 | H |  |
| SEP1 | SOC1 | 66 | L | 62 | Q |  |
| SEP1 | SOC1 | 66 | L | 55 | E |  |
| SEP1 | SOC1 | 69 | Y | 56 | F | ** |
| SEP1 | SOC1 | 69 | Y | 62 | Q |  |
| SEP1 | SOC1 | 134 | S | 62 | Q |  |
| SEP1 | SOC1 | 134 | S | 56 | F |  |
| ANR1 | SOC1 | 223 | E | 57 | A |  |
| SEP1 | SHP1 | 60 | S | 78 | G |  |
| SEP1 | SHP1 | 111 | L | 143 | G |  |
| SEP1 | SHP1 | 129 | R | 143 | G |  |
| SEP1 | SHP1 | 131 | L | 143 | G |  |
| SEP1 | SHP1 | 158 | Q | 157 | N | * |
| AGL6 | SOC1 | 46 | I | 56 | F |  |
| AGL6 | SOC1 | 46 | I | 62 | Q |  |
| AGL6 | SOC1 | 49 | S | 61 | M |  |
| AGL6 | SOC1 | 50 | R | 62 | Q |  |
| AGL6 | SOC1 | 53 | L | 56 | F |  |
| AGL6 | SOC1 | 53 | L | 62 | Q |  |
| AGL6 | FUL | 138 | Q | 125 | Q | * |
| AGL6 | AP1 | 138 | Q | 122 | K | * |
| SEP3 | STK | 160 | E | 140 | K | * |

Consistent overlap of a correlated mutation residue pair with a predicted interaction motif pair means that both residues of the correlated mutation residue pair each overlap with one of the two motifs that constitute an interaction motif pair. Such correlated mutation residue pairs are likely to be involved in determination of interaction specificity of MADS domain proteins.

Single stars indicate group of four correlated mutations in four different pairs of interacting proteins which overlap with one and the same interaction motif pair in those proteins. Double star indicates correlated mutation that overlaps an interaction motif pair which also overlaps two other correlated mutation pairs in two interacting protein pairs; these two pairs are however not listed in the table because these positions in the alignment map to a gap in the sequence of the respective proteins.
