# Supplementary material for: Conserved and variable correlated mutations in the plant MADS protein network
Source: BMC Genomics. 2010 Oct 28;11:607. doi: 10.1186/1471-2164-11-607 (PMC3017862; doi:10.1186/1471-2164-11-607)
Supplement: Additional file 7 — Conserved correlated mutations. This file contains correlated mutations which are conserved, i.e. appearing in more than one MADS domain protein (both intramolecular and intermolecular correlated mutations). [file 1471-2164-11-607-S7.DOC]

**Additional File 7. Conserved correlated mutations.**

**Conserved intramolecular correlated mutations^a^**

| AG | 83-111 83-115 186-188 186-189 186-190 186-223 189-190 206-220 206-221 206-223 206-224 206-225 206-227 212-213 212-215 212-216 212-217 212-218 212-225 212-226 212-233 212-240 213-215 213-216 213-217 213-218 213-226 213-233 213-234 213-236 213-240 215-216 215-217 215-218 215-219 215-225 215-226 215-233 215-234 215-236 215-240 215-241 216-217 216-218 216-219 216-225 216-226 216-233 216-234 216-236 216-240 216-241 217-234 217-240 218-219 218-220 218-224 218-225 218-226 218-229 218-230 218-233 218-234 218-235 218-236 218-239 218-240 218-241 218-243 218-244 219-220 219-224 219-225 219-226 219-229 219-230 219-233 219-235 219-236 219-239 219-240 219-243 221-223 221-224 221-225 221-227 221-238 223-224 223-225 223-227 227-228 227-229 227-230 227-231 227-232 227-233 227-235 227-236 227-237 227-238 227-239 227-242 227-243 227-244 227-245 227-246 227-247 227-248 227-249 227-250 227-251 228-229 228-230 228-231 228-232 228-234 228-235 228-236 228-237 228-239 228-243 228-244 229-230 229-232 229-233 229-234 229-235 229-236 229-237 229-239 229-243 229-244 229-245 229-246 229-248 230-231 230-232 230-233 230-234 230-235 230-236 230-237 230-239 230-242 230-243 230-244 230-245 230-247 230-248 230-249 231-232 231-235 231-237 231-239 231-242 231-243 231-244 231-245 231-247 231-248 231-249 231-250 232-234 232-235 232-236 232-237 232-239 232-242 232-243 232-244 232-245 232-246 232-247 232-248 232-250 233-234 233-235 233-236 233-237 233-239 233-240 233-241 233-242 233-243 233-244 233-245 233-247 233-248 233-249 233-250 234-235 234-236 234-237 234-239 234-240 234-241 234-242 234-243 234-244 234-245 234-247 234-249 234-250 235-236 235-237 235-239 235-242 235-243 235-244 235-245 235-246 235-247 235-248 235-249 235-250 235-251 235-252 236-237 236-239 236-240 236-241 236-242 236-243 236-244 236-245 236-246 236-247 236-248 236-250 237-239 237-240 237-241 237-242 237-243 237-244 237-245 237-246 237-247 237-248 237-249 237-250 239-242 239-243 239-244 239-245 239-246 239-247 239-248 239-249 239-250 239-251 239-252 240-241 240-250 241-250 242-243 242-244 242-245 242-246 242-247 242-248 242-249 242-250 242-251 243-244 243-245 243-246 243-247 243-248 243-249 243-250 243-251 243-252 244-245 244-246 244-247 244-248 244-249 244-250 244-251 244-252 245-246 245-247 245-248 245-249 245-250 245-251 245-252 246-247 246-248 246-249 246-250 246-251 247-248 247-249 247-250 247-251 247-252 248-249 248-250 248-251 248-252 249-250 249-251 249-252 250-251 |
| --- | --- |
| AGL12 | 191-193 191-194 191-195 191-198 191-199 191-203 191-204 191-205 191-206 191-207 191-209 192-199 192-206 193-194 193-195 193-196 193-198 193-199 193-203 193-204 193-205 193-206 193-207 193-209 194-195 194-196 194-197 194-198 194-199 194-203 194-204 194-205 194-206 194-207 194-209 195-196 195-197 195-198 195-199 195-203 195-204 195-205 195-206 195-207 195-208 195-209 196-197 196-198 196-199 196-203 196-204 196-205 196-206 196-207 196-208 196-209 197-198 197-199 197-203 197-204 197-205 197-206 197-207 197-208 197-209 198-199 198-203 198-204 198-205 198-206 198-207 198-208 198-209 199-203 199-204 199-205 199-206 199-207 199-208 199-209 203-204 203-205 203-206 203-207 203-208 203-209 204-205 204-206 204-207 204-208 204-209 205-206 205-207 205-208 205-209 206-207 206-208 206-209 207-208 207-209 208-209 |
| AGL14 | 88-89 150-186 166-190 166-192 172-173 175-188 181-184 181-199 181-201 181-210 183-185 184-186 184-188 184-197 184-198 184-199 184-200 184-201 184-205 184-206 185-209 186-188 186-189 186-192 186-193 186-198 186-199 186-200 186-205 187-188 187-190 187-191 187-192 187-193 190-191 190-192 190-204 191-192 191-193 191-194 191-195 191-201 191-202 192-193 192-194 192-195 192-200 192-201 192-202 193-194 193-195 193-200 193-201 193-202 194-195 195-201 197-198 197-199 197-200 197-201 197-205 197-206 197-207 197-210 197-211 197-212 198-199 198-200 198-201 198-205 198-206 198-207 198-210 199-200 199-201 199-205 199-206 199-207 199-210 199-211 199-212 200-201 200-205 200-206 200-207 200-211 201-202 201-204 201-205 201-206 201-207 201-210 202-204 202-205 205-206 205-207 205-210 205-211 206-207 206-210 206-211 206-212 207-210 207-211 210-211 210-212 211-212 |
| AGL15 | 196-197 196-198 196-199 196-200 196-201 196-204 196-205 196-214 196-216 198-199 198-200 198-201 198-203 198-204 198-205 198-214 198-216 199-200 199-201 199-204 199-205 199-214 199-216 200-201 200-204 200-205 200-214 200-216 203-204 203-205 203-214 203-223 203-226 203-229 205-214 205-216 205-223 205-226 205-229 227-228 227-230 227-231 227-232 227-233 227-234 227-235 227-236 227-237 227-238 227-240 227-241 227-242 228-230 228-231 228-232 228-233 228-234 228-235 228-236 228-237 228-238 228-240 228-241 228-242 230-231 230-232 230-233 230-234 230-235 230-237 230-238 231-232 231-233 231-234 231-235 231-236 231-237 231-238 231-240 231-241 231-242 232-233 232-234 232-235 232-236 232-237 232-238 232-240 232-241 232-242 233-234 233-235 233-236 233-237 233-238 233-240 233-241 233-242 234-235 234-236 234-237 234-238 234-240 234-241 234-242 235-236 235-237 235-238 235-240 235-241 235-242 236-237 236-238 236-240 236-241 236-242 237-238 237-240 237-241 237-242 238-240 238-241 238-242 240-241 240-242 241-242 |
| AGL16 | 212-214 212-216 212-217 212-218 212-219 212-220 212-221 212-222 221-222 221-223 221-224 221-225 221-226 221-227 221-228 221-229 221-230 221-232 221-233 221-234 221-235 222-223 222-224 222-225 222-226 222-227 222-228 222-229 222-230 222-232 222-233 222-234 222-235 223-224 223-225 223-226 223-227 223-228 223-229 223-230 223-232 223-233 223-234 223-235 224-225 224-226 224-227 224-228 224-229 224-230 224-232 224-233 224-234 224-235 225-226 225-227 225-228 225-229 225-230 225-232 225-233 225-234 225-235 226-227 226-228 226-229 226-230 226-232 226-233 226-234 226-235 227-228 227-229 227-230 227-232 227-233 227-234 227-235 228-229 228-230 228-232 228-233 228-234 228-235 229-230 229-232 229-233 229-234 229-235 230-232 230-233 230-234 230-235 232-233 232-234 232-235 233-234 233-235 234-235 237-238 |
| AGL17 | 200-205 200-218 201-205 201-218 205-218 |
| AGL19 | 200-201 200-202 200-203 200-204 200-205 200-206 200-207 200-208 200-209 200-210 200-211 200-212 200-213 200-214 200-215 200-216 201-202 201-203 201-204 201-205 201-206 201-207 201-208 201-209 201-210 201-211 201-212 201-214 201-215 201-216 202-203 202-204 202-205 202-206 202-207 202-208 202-209 202-210 202-211 202-212 202-213 202-214 202-215 202-216 202-217 203-204 203-205 203-206 203-207 203-208 203-209 203-210 203-211 203-212 203-213 203-214 203-215 203-216 203-217 204-205 204-206 204-207 204-208 204-209 204-210 204-211 204-212 204-213 204-214 204-215 204-216 204-217 205-206 205-207 205-208 205-209 205-210 205-211 205-212 205-213 205-214 205-215 206-207 206-208 206-209 206-210 206-211 206-212 206-213 206-214 206-215 206-216 206-217 207-208 207-209 207-210 207-211 207-212 207-213 207-214 207-215 207-216 207-217 208-209 208-210 208-211 208-212 208-213 208-214 208-215 208-216 208-217 209-210 209-211 209-212 209-213 209-214 209-215 209-216 209-217 210-211 210-212 210-213 210-214 210-215 210-216 210-217 211-212 211-213 211-214 211-215 211-216 211-217 212-213 212-214 212-215 212-216 212-217 213-214 213-215 213-216 213-217 214-215 214-216 214-217 215-216 215-217 216-217 |
| AGL21 | 172-173 173-196 194-195 194-196 194-197 194-198 194-199 194-200 194-201 194-203 194-204 194-205 194-206 194-207 194-208 194-210 194-211 194-212 194-213 194-214 194-216 194-217 194-218 195-196 195-197 195-198 195-199 195-200 195-201 195-203 195-204 195-205 195-206 195-207 195-208 195-210 195-211 195-212 195-213 195-215 195-216 195-218 197-198 197-199 197-200 197-201 197-203 197-204 197-205 197-206 197-207 197-208 197-210 197-211 197-212 197-213 197-214 197-215 197-216 197-217 197-218 198-199 198-200 198-201 198-203 198-204 198-205 198-206 198-207 198-208 198-210 198-211 198-212 198-213 198-214 198-215 198-216 198-217 198-218 199-200 199-201 199-203 199-204 199-205 199-206 199-207 199-208 199-210 199-211 199-212 199-213 199-214 199-215 199-216 199-217 199-218 200-201 200-203 200-204 200-205 200-206 200-207 200-208 200-211 200-212 200-213 200-215 200-216 200-217 200-218 201-203 201-204 201-205 201-206 201-207 201-208 201-210 201-211 201-212 201-213 201-214 201-215 201-216 201-217 201-218 203-204 203-205 203-206 203-207 203-208 203-209 203-210 203-211 203-212 203-213 203-214 203-215 203-216 203-217 203-218 204-205 204-206 204-207 204-208 204-210 204-211 204-212 204-213 204-215 204-216 204-217 205-206 205-207 205-208 205-209 205-210 205-211 205-212 205-213 205-214 205-215 205-216 205-217 205-218 206-207 206-208 206-209 206-210 206-211 206-212 206-213 206-214 206-215 206-216 206-217 206-218 207-208 207-209 207-210 207-211 207-212 207-213 207-214 207-215 207-216 207-217 207-218 208-209 208-210 208-211 208-212 208-213 208-214 208-215 208-216 208-217 208-218 209-210 209-211 209-212 209-213 209-214 209-215 209-216 209-217 209-218 210-211 210-212 210-213 210-214 210-215 210-216 210-217 210-218 211-212 211-213 211-214 211-215 211-216 211-217 211-218 212-213 212-214 212-215 212-216 212-217 212-218 212-219 212-220 212-221 212-222 212-223 212-224 212-225 213-214 213-215 213-216 213-217 213-218 213-219 213-220 213-222 213-223 213-224 213-225 213-226 214-215 214-216 214-217 214-218 214-219 214-220 214-221 214-222 214-223 214-224 214-225 214-226 215-216 215-217 215-218 216-217 216-218 217-218 219-220 219-221 219-222 219-223 219-224 219-225 219-226 220-221 220-222 220-223 220-224 220-225 220-226 221-222 221-223 221-224 221-225 221-226 222-223 222-224 222-225 222-226 223-224 223-225 223-226 224-225 224-226 225-226 |
| AGL24 | 180-181 181-184 184-186 184-197 184-201 185-187 197-198 197-199 197-200 197-201 197-202 197-203 197-204 197-206 197-207 197-209 198-199 198-200 198-201 198-202 198-203 198-204 198-206 198-207 198-208 198-209 198-210 198-212 198-216 198-217 199-200 199-201 199-202 199-203 199-204 199-206 199-207 199-208 199-209 199-210 199-212 199-216 199-217 200-201 200-202 200-203 200-204 200-206 200-207 200-208 200-209 200-210 200-212 200-216 200-217 201-202 201-203 201-204 201-206 201-207 201-208 201-209 201-210 201-212 201-216 201-217 202-203 202-204 202-206 202-207 202-208 202-209 202-210 202-212 202-216 202-217 203-204 203-206 203-207 203-208 203-209 203-210 203-212 203-216 203-217 204-206 204-207 204-208 204-209 204-210 204-212 204-216 204-217 206-207 206-208 206-209 206-210 206-212 206-216 206-217 207-208 207-209 207-210 207-212 207-216 207-217 208-209 208-210 208-212 208-216 208-217 209-210 209-212 209-216 209-217 210-212 210-216 210-217 212-216 212-217 216-217 |
| AGL42 | 193-194 193-195 193-196 193-197 193-198 193-199 193-201 193-202 193-203 193-204 193-206 193-207 194-195 194-196 194-197 194-198 194-199 194-201 194-202 194-203 194-204 194-206 194-207 195-196 195-197 195-198 195-199 195-201 195-202 195-203 195-204 195-206 195-207 196-197 196-198 196-199 196-201 196-202 196-203 196-204 196-206 196-207 197-198 197-199 197-201 197-202 197-203 197-204 197-206 197-207 198-199 198-201 198-202 198-203 198-204 198-206 198-207 199-201 199-202 199-203 199-204 199-206 199-207 201-202 201-203 201-204 201-206 201-207 202-203 202-204 202-206 202-207 203-204 203-206 203-207 204-206 204-207 206-207 |
| AGL6 | 31-32 31-34 34-35 186-187 186-192 187-192 192-213 192-214 193-200 193-211 193-218 211-218 211-219 211-229 213-214 214-223 218-229 218-230 218-231 218-234 218-235 218-240 218-241 218-242 236-237 236-238 236-239 236-241 236-242 236-243 236-244 236-245 236-246 236-247 236-248 236-249 236-250 237-238 237-239 237-241 237-242 237-243 237-244 237-245 237-246 237-247 237-248 237-249 237-250 238-239 238-241 238-242 238-243 238-244 238-245 238-246 238-247 238-248 238-249 238-250 239-241 239-242 239-243 239-244 239-245 239-246 239-247 239-248 239-249 239-250 241-242 241-243 241-244 241-245 241-246 241-247 241-248 241-249 241-250 242-243 242-244 242-245 242-246 242-247 242-248 242-249 242-250 243-244 243-245 243-246 243-247 243-248 243-249 243-250 244-245 244-246 244-247 244-248 244-249 244-250 245-246 245-247 245-248 245-249 245-250 246-247 246-248 246-249 246-250 247-248 247-249 247-250 248-249 248-250 249-250 |
| AGL71 | 59-72 59-94 72-74 72-107 72-133 147-203 151-188 152-194 152-203 155-191 184-191 185-186 185-187 185-188 185-189 185-192 185-193 185-198 185-199 185-200 185-201 186-188 186-193 186-199 186-200 186-203 187-188 187-189 187-192 187-193 187-198 187-199 187-200 187-201 188-189 188-190 188-191 188-193 188-194 188-195 188-198 188-200 188-201 188-203 189-190 189-193 189-194 189-195 189-198 189-199 189-200 189-201 189-203 190-191 190-195 191-195 191-198 193-194 193-199 193-200 193-201 193-203 194-199 194-200 194-201 194-203 195-198 198-200 199-201 199-203 200-201 200-203 201-203 |
| AGL72 | 186-187 186-189 186-190 186-191 186-194 186-195 186-196 186-197 186-199 186-202 186-203 186-205 187-189 187-190 187-191 187-194 187-195 187-196 187-197 187-199 187-202 187-203 187-205 189-190 189-191 189-194 189-195 189-196 189-197 189-199 189-202 189-203 189-205 190-191 190-194 190-195 190-196 190-197 190-199 190-202 190-203 190-205 191-194 191-195 191-196 191-197 191-199 191-202 191-203 191-205 194-195 194-196 194-197 194-199 194-202 194-203 194-205 195-196 195-197 195-199 195-202 195-203 195-205 196-197 196-199 196-202 196-203 196-205 197-199 197-202 197-203 197-205 199-202 199-203 199-205 202-203 202-205 203-205 |
| ANR1 | 97-107 152-162 187-188 187-190 187-193 187-199 187-201 206-207 206-208 206-209 206-210 206-211 206-213 206-214 206-215 206-216 206-217 206-218 206-219 206-220 206-221 208-209 208-210 208-211 208-213 208-214 208-215 208-216 208-217 208-218 208-219 208-220 208-221 210-211 210-213 210-214 210-215 210-216 210-217 210-218 210-219 210-220 210-221 211-213 211-214 211-215 211-216 211-217 211-218 211-219 211-220 211-221 213-214 213-215 213-216 213-217 213-218 213-219 213-220 213-221 214-215 214-216 214-217 214-218 214-219 214-220 214-221 215-216 215-217 215-218 215-219 215-220 215-221 216-217 216-218 216-219 216-220 216-221 217-218 217-219 217-220 217-221 217-222 217-223 217-224 217-225 217-227 217-229 217-230 217-231 218-219 218-220 218-221 219-220 219-221 220-221 220-222 223-225 223-229 223-230 223-231 224-225 224-229 224-230 224-231 225-227 225-228 225-229 225-230 225-231 227-229 227-231 228-229 228-230 228-231 229-230 229-231 230-231 |
| AP1 | 36-40 36-100 59-94 73-133 155-158 155-160 171-173 171-174 171-175 171-217 173-174 176-217 200-201 200-202 200-203 200-204 201-202 201-203 201-204 201-206 201-207 201-208 201-209 201-214 202-203 202-204 202-205 202-206 202-207 202-208 202-209 202-225 203-204 203-205 203-206 203-207 203-208 203-209 204-205 204-206 204-207 204-208 204-209 204-225 205-206 205-207 205-208 205-209 206-207 206-208 206-209 207-208 217-220 217-242 217-244 221-222 221-223 221-224 221-225 221-226 221-227 221-228 221-230 221-231 221-233 221-234 221-235 221-237 221-238 221-240 221-241 221-243 221-244 221-245 229-230 229-231 229-232 229-233 229-234 229-235 229-236 229-237 229-238 229-239 229-240 229-241 229-242 229-243 229-244 229-245 229-246 229-249 229-250 229-251 229-252 232-233 232-234 232-235 232-236 232-237 232-238 232-239 232-240 232-241 232-242 232-243 232-244 232-245 232-247 232-248 232-249 232-250 232-251 232-252 233-234 233-235 233-236 233-237 233-238 233-239 233-240 233-241 233-242 233-243 233-244 233-245 233-246 233-247 233-248 233-249 233-250 233-251 233-252 234-235 234-236 234-237 234-238 234-239 234-240 234-241 234-242 234-243 234-244 234-245 234-248 234-249 234-250 234-251 234-252 235-236 235-237 235-238 235-239 235-240 235-241 235-243 235-244 235-245 235-246 235-247 235-248 235-249 235-250 235-251 235-252 236-237 236-238 236-239 236-240 236-241 236-242 236-243 236-244 236-245 236-246 236-247 236-248 236-249 236-250 236-251 236-252 237-238 237-239 237-240 237-241 237-243 237-244 237-245 237-246 237-247 237-248 237-249 238-239 238-240 238-241 238-242 238-243 238-244 238-245 238-246 238-247 238-248 238-249 238-250 238-251 238-252 238-253 238-254 239-240 239-241 239-242 239-243 239-244 239-245 239-246 239-247 239-248 239-249 239-250 239-251 239-252 239-253 239-254 240-241 240-243 240-244 240-245 240-246 240-247 240-248 240-249 240-250 240-251 240-252 240-253 240-254 241-242 241-243 241-244 241-245 241-246 241-247 241-248 241-249 241-250 241-251 241-252 241-253 241-254 242-243 242-244 243-244 243-245 243-246 243-250 243-251 243-252 243-253 243-254 244-245 244-246 244-247 244-248 244-249 244-250 244-251 244-252 244-253 244-254 245-250 245-252 245-254 246-247 246-248 246-249 246-250 246-251 246-252 246-253 246-254 247-248 247-249 247-250 247-251 247-252 247-253 248-249 248-250 248-251 248-252 248-253 249-250 249-251 249-252 249-253 249-254 250-251 250-252 250-253 250-254 251-252 251-253 251-254 252-253 252-254 253-254 |
| CAL | 241-242 241-243 241-244 241-245 241-246 241-247 241-248 241-249 241-250 241-251 241-252 242-243 242-244 242-245 242-246 242-247 242-248 242-249 242-250 242-251 242-252 243-244 243-245 243-246 243-247 243-248 243-249 243-250 243-251 243-252 244-245 244-246 244-247 244-248 244-249 244-250 244-251 244-252 245-246 245-247 245-248 245-249 245-250 245-251 245-252 246-247 246-248 246-249 246-250 246-251 246-252 247-248 247-249 247-250 247-251 247-252 248-249 248-250 248-251 248-252 249-250 249-251 249-252 250-251 250-252 251-252 |
| FUL | 36-40 36-41 36-58 153-163 160-164 207-208 207-210 207-213 207-215 207-216 207-217 207-219 207-220 207-225 207-226 207-228 208-210 208-213 208-215 208-216 208-217 208-219 208-220 208-225 219-220 219-221 219-222 219-223 219-224 219-225 219-226 219-227 219-228 219-229 219-231 219-235 220-221 220-222 220-223 220-224 220-225 220-226 220-227 220-228 220-229 220-231 220-235 220-236 220-237 220-238 221-222 221-223 221-224 221-225 221-226 221-227 221-228 221-229 221-231 221-235 221-236 221-237 221-238 222-223 222-224 222-225 222-226 222-227 222-228 222-229 222-231 222-234 222-235 222-236 222-237 223-224 223-225 223-226 223-227 223-228 223-229 223-231 223-234 223-235 223-236 223-237 223-238 224-225 224-226 224-227 224-228 224-229 224-231 224-234 224-235 224-236 224-237 224-238 225-226 225-227 225-228 225-229 225-231 225-234 225-235 225-236 225-237 225-238 226-227 226-228 226-229 226-231 226-234 226-235 226-236 226-237 226-238 227-228 227-229 227-231 227-234 227-235 227-236 227-237 227-238 228-229 228-231 228-234 228-235 228-236 228-237 228-238 229-230 229-231 229-233 229-234 229-235 229-236 229-237 229-238 230-232 231-235 231-236 231-237 231-238 234-235 234-236 234-237 234-238 235-236 235-237 235-238 236-237 236-238 237-238 240-241 |
| SEP1 | 58-94 58-108 97-102 98-105 98-108 160-177 160-199 160-204 218-244 230-231 230-232 230-234 230-235 230-236 230-237 230-238 230-239 230-240 230-241 230-242 230-243 230-244 230-245 230-246 231-232 231-233 231-234 231-235 231-236 231-237 231-238 231-239 231-240 231-241 231-242 231-243 231-244 231-245 231-246 232-233 232-234 232-235 232-236 232-238 232-240 232-241 232-242 232-243 232-244 232-246 233-235 233-236 233-238 233-239 233-240 233-241 233-242 233-243 233-244 234-235 234-236 234-237 234-238 234-239 234-240 234-241 234-242 234-243 234-244 234-245 234-246 235-236 235-237 235-238 235-239 235-240 235-241 235-242 235-243 235-244 235-245 235-246 236-237 236-238 236-239 236-240 236-241 236-242 236-243 236-244 236-245 236-246 237-238 237-239 237-240 237-241 237-242 237-243 237-244 237-246 238-239 238-240 238-241 238-242 238-243 238-244 238-245 238-246 239-240 239-241 239-242 239-243 239-244 239-245 239-246 240-241 240-242 240-243 240-244 240-245 240-246 241-242 241-243 241-244 241-246 242-243 242-244 242-245 242-246 243-244 243-245 243-246 244-245 244-246 |
| SEP2 | 229-230 229-231 229-232 229-233 229-234 229-235 229-238 229-242 229-243 229-244 229-245 230-231 230-232 230-233 230-234 230-235 230-236 230-237 230-238 230-242 230-243 230-244 230-245 231-232 231-233 231-234 231-235 231-242 231-243 231-244 231-245 231-246 232-233 232-234 232-235 232-236 232-237 232-238 232-242 232-243 232-244 232-245 232-246 233-234 233-235 233-236 233-237 233-238 233-242 233-243 233-244 233-245 233-246 234-235 234-242 234-243 234-244 234-245 234-246 235-236 235-237 235-238 235-242 235-243 235-244 235-245 235-246 235-248 236-237 236-238 236-243 236-244 237-238 237-243 237-244 238-243 238-244 242-243 242-244 242-245 242-246 242-248 243-244 243-245 243-246 243-248 244-245 244-246 244-248 245-246 245-248 246-248 |
| SEP3 | 31-32 31-34 36-41 36-58 36-103 58-97 58-111 68-99 68-103 100-105 101-108 101-111 150-220 163-178 163-200 163-205 170-193 193-244 193-245 193-246 193-247 211-213 211-214 211-215 211-216 211-217 211-219 211-220 211-221 211-222 211-225 211-226 211-227 211-231 211-232 211-233 211-235 211-236 214-215 214-217 214-219 214-220 214-222 214-223 214-224 214-225 214-226 214-228 214-230 214-232 214-235 214-236 216-217 216-218 216-220 216-221 217-218 217-219 217-220 217-221 217-222 217-223 217-224 217-225 217-226 217-227 217-232 217-234 217-235 217-236 218-220 218-221 219-220 219-223 219-224 219-225 219-226 219-227 219-228 219-230 219-231 219-232 219-234 219-235 219-236 220-221 220-222 220-223 220-224 220-225 220-226 220-227 220-228 220-229 220-230 220-231 220-232 220-234 220-235 220-236 220-237 220-239 220-240 220-242 223-224 223-225 223-227 223-229 223-231 223-232 223-234 223-235 223-236 223-237 223-239 223-240 223-242 224-225 224-226 224-227 224-228 224-229 224-230 224-231 224-232 224-234 224-235 224-236 224-237 224-239 224-240 224-242 225-226 225-227 225-228 225-229 225-231 225-232 225-233 225-234 225-235 225-236 225-237 225-239 225-240 225-242 226-227 226-228 226-229 226-230 226-231 226-232 226-233 226-234 226-235 226-236 226-237 226-239 226-240 226-242 227-228 227-229 227-230 227-233 227-234 227-235 227-236 227-237 227-239 227-240 227-242 228-229 228-230 228-231 228-232 228-234 228-235 228-236 228-237 228-239 228-240 228-242 229-230 229-231 229-232 229-234 229-235 229-236 229-237 229-239 229-240 229-242 230-231 230-232 230-233 230-234 230-235 230-236 230-237 230-239 230-240 230-242 231-232 231-233 231-235 231-236 231-237 231-239 231-240 231-242 232-233 232-235 232-236 232-237 232-239 232-240 232-242 233-235 233-236 233-237 233-238 233-239 233-240 234-235 234-236 234-237 234-239 234-240 234-242 235-236 235-237 235-239 235-240 235-242 236-237 236-239 236-240 236-242 237-239 237-240 237-242 239-240 239-242 240-242 244-245 244-246 244-247 245-246 245-247 246-247 |
| SEP4-I | 89-90 155-205 181-182 182-185 182-191 182-213 185-188 185-199 187-188 187-189 187-234 187-236 188-189 188-191 188-234 188-236 191-205 191-213 191-217 205-213 205-234 205-236 207-208 207-214 207-221 207-223 207-224 207-227 207-230 208-214 208-221 208-223 208-224 208-227 208-230 213-217 213-236 214-221 214-223 214-224 214-227 214-230 214-239 214-240 214-241 214-243 214-248 221-223 221-224 221-227 221-230 221-239 221-248 223-224 223-227 223-230 224-227 224-230 224-239 224-240 224-241 224-243 224-248 227-230 227-239 227-240 227-241 227-243 227-248 230-239 230-240 230-241 230-243 230-248 234-236 239-240 239-241 239-243 239-248 240-241 240-243 240-248 241-243 241-248 243-248 |
| SHP1 | 226-227 226-228 226-229 226-230 |
| SHP2 | 215-216 215-217 215-219 215-220 215-221 215-223 215-224 215-225 215-228 215-229 215-231 215-232 215-233 215-234 215-235 215-236 215-241 216-217 216-219 216-220 216-221 216-223 216-224 216-225 216-228 216-229 216-231 216-232 216-233 216-234 216-235 216-236 216-241 217-219 217-220 217-221 217-223 217-224 217-225 217-228 217-229 217-231 217-232 217-233 217-234 217-235 217-236 217-241 219-220 219-221 219-223 219-224 219-225 219-228 219-229 219-231 219-232 219-233 219-234 219-235 219-236 220-221 220-223 220-224 220-225 220-228 220-229 220-232 220-233 220-234 220-235 221-223 221-224 221-225 221-228 221-229 221-231 221-232 221-233 221-234 221-235 221-236 221-238 221-239 221-241 223-224 223-225 223-228 223-229 223-231 223-232 223-233 223-234 223-235 223-236 223-238 223-239 223-241 224-225 224-228 224-229 224-231 224-232 224-233 224-234 224-235 224-236 225-228 225-229 225-231 225-232 225-233 225-234 225-235 225-236 225-238 225-239 225-241 225-243 228-229 228-231 228-232 228-233 228-234 228-235 228-236 228-238 228-239 228-241 228-243 229-231 229-232 229-233 229-234 229-235 229-236 229-238 229-239 229-241 229-243 231-232 231-233 231-234 231-235 231-236 231-238 231-239 231-241 231-243 232-233 232-234 232-235 232-236 232-238 232-239 232-241 232-243 233-234 233-235 233-236 233-238 233-239 233-241 233-243 234-235 234-236 234-238 234-239 234-241 234-243 235-236 235-238 235-239 235-241 235-243 236-238 236-239 236-241 236-243 238-239 238-241 238-243 239-241 239-243 241-243 |
| SOC1 | 34-35 155-156 163-166 199-200 199-201 199-202 199-203 199-204 199-205 199-206 199-207 199-208 199-209 199-210 199-211 199-212 201-202 201-203 201-204 201-205 201-206 201-207 201-208 201-209 201-210 201-211 201-212 202-204 202-205 202-206 202-207 202-208 202-209 202-210 202-211 202-212 202-213 203-206 203-208 204-205 204-206 204-207 204-208 204-209 204-210 204-211 204-212 204-213 205-206 205-207 205-208 205-209 205-210 205-211 205-212 206-207 206-208 206-209 206-210 206-211 206-212 206-213 207-208 207-209 207-210 207-211 207-212 207-213 208-209 208-210 208-211 208-212 208-213 209-210 209-211 209-212 210-211 210-212 210-213 211-212 211-213 212-213 |
| STK | 87-89 87-121 87-147 166-218 166-227 169-172 169-174 170-171 203-204 203-205 203-218 203-221 203-224 204-205 204-207 204-216 204-218 205-208 205-218 205-221 205-224 205-226 207-208 207-216 207-217 207-219 208-215 208-216 208-217 208-218 208-219 208-221 208-222 208-223 208-224 208-225 208-227 215-216 215-217 215-218 215-219 215-220 215-221 215-222 215-223 215-224 215-225 215-227 216-217 216-218 216-219 216-220 216-221 216-222 216-223 216-225 216-227 217-218 217-219 217-220 217-221 217-222 217-223 217-224 217-225 217-227 218-219 218-220 218-221 218-222 218-223 218-224 218-225 218-226 218-227 218-228 219-220 219-221 219-222 219-223 219-224 219-225 219-227 220-221 220-222 220-223 220-224 220-225 220-227 221-222 221-223 221-224 221-225 221-227 222-223 222-224 222-225 222-227 223-224 223-225 223-227 224-225 224-226 224-227 224-228 225-227 226-227 227-228 |
| SVP | 159-163 163-166 185-186 185-187 185-188 186-187 186-188 186-206 187-188 187-189 187-190 187-207 187-209 188-189 188-190 188-207 188-209 190-207 203-204 206-207 206-209 207-209 |

**^a^** Conserved intramolecular correlated mutations (correlated mutations that occur at homologous positions in at least one additional protein). Numbers here refer to sequence positions, the corresponding amino acid residues can be found in Additional File 3.

**Conserved intermolecular correlated mutations^a^**

|  | **Correlated mutation pair 1** | | | | | | **Correlated mutation pair 2** | | | | | | | |
| --- | --- | --- | --- | --- | --- | --- | --- | --- | --- | --- | --- | --- | --- | --- |
| **Dist^b^** | **Protein 1** | **Position 1** | | **Protein 2** | | **Position 2** | **Protein 1** | **Position 1** | | **Protein 2** | | **Position 2** | |  |
|  | AGL6 | 37 | AP1 | | 41 | | STK | | 52 | | SEP3 | | 41 |  |
|  | AGL6 | 37 | AP1 | | 36 | | SOC1 | | 37 | | AGL6 | | 36 |  |
|  | AGL6 | 138 | AP1 | | 122 | | SEP1 | | 140 | | AGL6 | | 120 |  |
|  | AGL6 | 49 | FUL | | 158 | | SEP1 | | 49 | | SHP1 | | 157 |  |
|  | AGL6 | 120 | SEP1 | | 140 | | AP1 | | 122 | | AGL6 | | 138 |  |
|  | AGL6 | 53 | SEP1 | | 91 | | SEP3 | | 53 | | AGL6 | | 89 |  |
|  | AGL6 | 120 | SEP1 | | 125 | | SEP3 | | 125 | | AGL6 | | 123 |  |
|  | AGL6 | 123 | SEP3 | | 125 | | SEP1 | | 125 | | AGL6 | | 120 |  |
|  | AGL6 | 89 | SEP3 | | 46 | | SEP1 | | 91 | | SOC1 | | 46 |  |
|  | AGL6 | 89 | SEP3 | | 45 | | SEP1 | | 91 | | SOC1 | | 45 |  |
|  | AGL6 | 89 | SEP3 | | 42 | | SEP1 | | 91 | | SOC1 | | 42 |  |
|  | AGL6 | 34 | SOC1 | | 36 | | SOC1 | | 34 | | AGL6 | | 36 |  |
|  | AGL6 | 34 | SOC1 | | 34 | | SOC1 | | 34 | | AGL6 | | 34 |  |
| ***** | AGL6 | 46 | SOC1 | | 65 | | SOC1 | | 46 | | SEP1 | | 66 |  |
|  | AGL6 | 30 | SOC1 | | 36 | | SHP1 | | 30 | | SEP1 | | 36 |  |
| * | AGL6 | 54 | SOC1 | | 56 | | SOC1 | | 54 | | AGL6 | | 56 |  |
|  | AGL6 | 54 | SOC1 | | 54 | | SOC1 | | 54 | | AGL6 | | 54 |  |
|  | AGL6 | 53 | SOC1 | | 90 | | SEP3 | | 53 | | AGL6 | | 89 |  |
| * | AGL6 | 46 | SOC1 | | 46 | | SOC1 | | 46 | | AGL6 | | 46 |  |
|  | AGL6 | 36 | SOC1 | | 36 | | SOC1 | | 36 | | AGL6 | | 36 |  |
| * | AGL6 | 56 | SOC1 | | 56 | | SOC1 | | 56 | | AGL6 | | 56 |  |
| * | AGL6 | 58 | SOC1 | | 43 | | SOC1 | | 58 | | AGL6 | | 43 |  |
|  | AGL6 | 244 | SOC1 | | 174 | | SEP1 | | 243 | | SHP1 | | 180 |  |
|  | AGL6 | 36 | SOC1 | | 37 | | AP1 | | 36 | | AGL6 | | 37 |  |
| * | AGL6 | 56 | SOC1 | | 46 | | SOC1 | | 56 | | AGL6 | | 46 |  |
| * | AGL6 | 46 | SOC1 | | 54 | | SOC1 | | 46 | | AGL6 | | 54 |  |
|  | AGL6 | 43 | SOC1 | | 152 | | SOC1 | | 43 | | SEP3 | | 156 |  |
|  | ANR1 | 65 | SOC1 | | 73 | | SEP1 | | 65 | | AP1 | | 74 |  |
|  | ANR1 | 229 | SOC1 | | 150 | | SEP1 | | 220 | | SHP1 | | 150 |  |
|  | AP1 | 55 | SEP1 | | 102 | | FUL | | 55 | | SOC1 | | 101 |  |
|  | AP1 | 74 | SEP1 | | 65 | | SOC1 | | 73 | | ANR1 | | 65 |  |
|  | AP1 | 72 | SEP1 | | 98 | | STK | | 83 | | SEP3 | | 101 |  |
|  | FUL | 55 | SOC1 | | 104 | | SOC1 | | 55 | | SEP1 | | 105 |  |
|  | SEP1 | 181 | SHP1 | | 157 | | SHP1 | | 180 | | SEP1 | | 158 |  |
|  | SEP1 | 81 | SHP1 | | 180 | | SHP1 | | 78 | | SEP1 | | 181 |  |
|  | SEP1 | 181 | SHP1 | | 78 | | SHP1 | | 180 | | SEP1 | | 81 |  |
|  | SEP1 | 81 | SHP1 | | 78 | | SHP1 | | 78 | | SEP1 | | 81 |  |
|  | SEP1 | 158 | SHP1 | | 157 | | SHP1 | | 157 | | SEP1 | | 158 |  |
|  | SEP1 | 81 | SHP1 | | 157 | | SHP1 | | 78 | | SEP1 | | 158 |  |
|  | SEP1 | 181 | SHP1 | | 180 | | SHP1 | | 180 | | SEP1 | | 181 |  |
|  | SEP1 | 243 | SHP1 | | 180 | | AGL6 | | 244 | | SOC1 | | 174 |  |
|  | SEP1 | 158 | SHP1 | | 78 | | SHP1 | | 157 | | SEP1 | | 81 |  |
|  | SEP1 | 220 | SHP1 | | 150 | | ANR1 | | 229 | | SOC1 | | 150 |  |
|  | SEP1 | 49 | SHP1 | | 157 | | AGL6 | | 49 | | FUL | | 158 |  |
| * | SEP1 | 36 | SHP1 | | 30 | | SOC1 | | 36 | | AGL6 | | 30 |  |
|  | SEP1 | 105 | SOC1 | | 55 | | SOC1 | | 104 | | FUL | | 55 |  |
| * | SEP1 | 66 | SOC1 | | 46 | | SOC1 | | 65 | | AGL6 | | 46 |  |
| * | SEP1 | 45 | SOC1 | | 68 | | SOC1 | | 45 | | SEP1 | | 69 |  |
|  | SEP1 | 108 | SOC1 | | 36 | | STK | | 122 | | SEP3 | | 36 |  |
|  | SEP1 | 91 | SOC1 | | 46 | | AGL6 | | 89 | | SEP3 | | 46 |  |
|  | SEP1 | 91 | SOC1 | | 45 | | AGL6 | | 89 | | SEP3 | | 45 |  |
|  | SEP1 | 102 | SOC1 | | 55 | | SOC1 | | 101 | | FUL | | 55 |  |
|  | SEP1 | 108 | SOC1 | | 37 | | SEP3 | | 111 | | STK | | 52 |  |
|  | SEP1 | 91 | SOC1 | | 42 | | AGL6 | | 89 | | SEP3 | | 42 |  |
|  | SEP1 | 143 | STK | | 117 | | SVP | | 142 | | SOC1 | | 102 |  |
|  | SEP1 | 146 | STK | | 160 | | STK | | 160 | | SEP1 | | 146 |  |
|  | SEP3 | 156 | SOC1 | | 43 | | SOC1 | | 152 | | AGL6 | | 43 |  |
|  | SEP3 | 36 | STK | | 122 | | SOC1 | | 36 | | SEP1 | | 108 |  |
|  | SEP3 | 111 | STK | | 52 | | SEP1 | | 108 | | SOC1 | | 37 |  |
|  | SEP3 | 101 | STK | | 83 | | SEP1 | | 98 | | AP1 | | 72 |  |
| * | SEP3 | 41 | STK | | 52 | | AP1 | | 41 | | AGL6 | | 37 |  |
|  | SHP1 | 27 | SOC1 | | 27 | | SOC1 | | 27 | | SHP1 | | 27 |  |
|  | SHP1 | 159 | SOC1 | | 159 | | SOC1 | | 159 | | SHP1 | | 159 |  |
|  | SOC1 | 102 | SVP | | 142 | | STK | | 117 | | SEP1 | | 143 |  |

**^a^** Pairs of conserved intermolecular correlated mutations (correlated mutations that occur at homologous positions in two pairs of interacting proteins).

**^b^** Dist indicates conserved intermolecular correlated mutations which can be mapped to the structure of the MADS domain and for which the distance between the two residues is lower than 15A.

In addition to the set of conserved intermolecular correlated mutations consisting of two pairs, shown above, there are two larger groups:

- AGL6-89 – SEP3-53, SOC1-90 – AGL6-53 and SEP1-91 – AGL6-53
- FUL-55 – SOC1-101, AP1-55 – SEP1-102 and SOC1-55 – SEP1-102
