# Supplementary material for: Conserved and variable correlated mutations in the plant MADS protein network
Source: BMC Genomics. 2010 Oct 28;11:607. doi: 10.1186/1471-2164-11-607 (PMC3017862; doi:10.1186/1471-2164-11-607)
Supplement: Additional file 8 — Prediction of helices in K-domain. This file contains predicted helices in the K-domain. [file 1471-2164-11-607-S8.DOC]

**Additional File 8. Prediction of helices in K-domain**

| **Protein** | **Num. helices** | **Positions^a^** | | | | | | | |
| --- | --- | --- | --- | --- | --- | --- | --- | --- | --- |
| AG | 1 | 138 | 152 |  |  |  |  |  |  |
| AGL12 | 3 | 98 | 113 | 149 | 163 | 167 | 180 |  |  |
| AGL13 | 3 | 86 | 101 | 116 | 134 | 136 | 165 |  |  |
| AGL14 | 2 | 74 | 79 | 117 | 164 |  |  |  |  |
| AGL15 | 2 | 84 | 104 | 140 | 168 |  |  |  |  |
| AGL16 | 2 | 116 | 133 | 173 | 186 |  |  |  |  |
| AGL17 | 3 | 83 | 111 | 121 | 134 | 139 | 158 |  |  |
| AGL19 | 3 | 73 | 78 | 90 | 110 | 116 | 168 |  |  |
| AGL21 | 3 | 86 | 110 | 117 | 134 | 138 | 158 |  |  |
| AGL24 | 3 | 92 | 113 | 118 | 134 | 157 | 179 |  |  |
| AGL42 | 1 | 139 | 171 |  |  |  |  |  |  |
| AGL6 | 2 | 118 | 135 | 144 | 166 |  |  |  |  |
| AGL63 | 2 | 94 | 111 | 174 | 187 |  |  |  |  |
| AGL71 | 2 | 82 | 102 | 141 | 173 |  |  |  |  |
| AGL72 | 1 | 140 | 167 |  |  |  |  |  |  |
| ANR1 | 1 | 84 | 106 |  |  |  |  |  |  |
| AP1 | 3 | 89 | 110 | 121 | 139 | 148 | 173 |  |  |
| AP3 | 3 | 82 | 106 | 114 | 136 | 160 | 173 |  |  |
| CAL | 4 | 94 | 112 | 121 | 150 | 152 | 175 | 184 | 197 |
| FUL | 3 | 92 | 111 | 120 | 137 | 160 | 173 |  |  |
| PI | 1 | 79 | 110 |  |  |  |  |  |  |
| SEP1 | 2 | 92 | 114 | 119 | 139 |  |  |  |  |
| SEP2 | 2 | 92 | 114 | 119 | 139 |  |  |  |  |
| SEP3 | 2 | 94 | 114 | 122 | 144 |  |  |  |  |
| SEP4-I | 3 | 81 | 94 | 120 | 140 | 158 | 178 |  |  |
| SHP1 | 2 | 104 | 123 | 136 | 149 |  |  |  |  |
| SHP2 | 2 | 104 | 123 | 134 | 150 |  |  |  |  |
| SOC1 | 3 | 83 | 113 | 118 | 135 | 140 | 169 |  |  |
| STK | 3 | 89 | 110 | 119 | 140 | 148 | 162 |  |  |
| SVP | 3 | 75 | 78 | 118 | 134 | 157 | 170 |  |  |

^a^ Start- and end-positions of predicted helices.
