# Supplementary material for: Conserved and variable correlated mutations in the plant MADS protein network
Source: BMC Genomics. 2010 Oct 28;11:607. doi: 10.1186/1471-2164-11-607 (PMC3017862; doi:10.1186/1471-2164-11-607)
Supplement: Additional file 9 — Predicted intramolecular contacts between helices. This file contains contacts predicted between K-domain helices using correlated mutations. [file 1471-2164-11-607-S9.DOC]

**Additional File 9. Predicted intramolecular contacts between helices^a^**

| Protein | Helix_start1 | Helix_end1 | Helix_start2 | Helix_end2 | Correlated mutation | |
| --- | --- | --- | --- | --- | --- | --- |
| AP1 | 89 | 110 | 148 | 173 | 110 | 162 |
| SEP1 | 92 | 114 | 119 | 139 | 105 | 123 |
| SEP1 | 92 | 114 | 119 | 139 | 105 | 134 |
| SEP1 | 92 | 114 | 119 | 139 | 97 | 134 |
| SEP1 | 92 | 114 | 119 | 139 | 102 | 130 |
| SEP1 | 92 | 114 | 119 | 139 | 93 | 130 |
| SEP1 | 92 | 114 | 119 | 139 | 93 | 123 |
| SEP1 | 92 | 114 | 119 | 139 | 102 | 134 |
| SEP1 | 92 | 114 | 119 | 139 | 102 | 123 |
| SEP1 | 92 | 114 | 119 | 139 | 98 | 134 |
| SEP1 | 92 | 114 | 119 | 139 | 98 | 130 |
| SEP1 | 92 | 114 | 119 | 139 | 93 | 134 |
| SEP1 | 92 | 114 | 119 | 139 | 98 | 123 |
| SEP3 | 94 | 114 | 122 | 144 | 108 | 135 |
| SEP3 | 94 | 114 | 122 | 144 | 103 | 128 |

^a^ Intramolecular correlated mutations connecting two helices.
