# Supplementary material for: Conserved and variable correlated mutations in the plant MADS protein network
Source: BMC Genomics. 2010 Oct 28;11:607. doi: 10.1186/1471-2164-11-607 (PMC3017862; doi:10.1186/1471-2164-11-607)
Supplement: Additional file 10 — Predicted intramolecular contacts helices - MADS/I domain. This file contains contacts predicted between K-domain helices and the MADS/I domain. [file 1471-2164-11-607-S10.DOC]

**Additional File 10. Predicted intramolecular contacts helices – MADS/I domain^a^**

| **Protein** | **Helix start** | **Helix end** | **Correlated mutation** | | **Conserved** |  |
| --- | --- | --- | --- | --- | --- | --- |
| AG | 138 | 152 | 83 | 142 |  |  |
| AG | 138 | 152 | 83 | 138 |  |  |
| AG | 138 | 152 | 83 | 146 |  |  |
| AG | 138 | 152 | 83 | 145 |  |  |
| AP1 | 89 | 110 | 74 | 89 |  |  |
| AP1 | 89 | 110 | 40 | 100 |  |  |
| AP1 | 89 | 110 | 36 | 100 | * |  |
| AP1 | 89 | 110 | 59 | 94 | * |  |
| AP1 | 89 | 110 | 37 | 100 |  |  |
| AP1 | 89 | 110 | 74 | 100 |  |  |
| AP1 | 89 | 110 | 74 | 106 |  |  |
| AP1 | 89 | 110 | 73 | 100 |  |  |
| AP1 | 121 | 139 | 73 | 133 | * |  |
| AP1 | 148 | 173 | 72 | 149 |  |  |
| AP1 | 148 | 173 | 40 | 159 |  |  |
| AP1 | 148 | 173 | 36 | 159 |  |  |
| AP1 | 148 | 173 | 37 | 159 |  |  |
| AP1 | 148 | 173 | 46 | 160 |  |  |
| AP1 | 148 | 173 | 46 | 158 |  |  |
| AP1 | 148 | 173 | 46 | 173 |  |  |
| AP1 | 148 | 173 | 73 | 159 |  |  |
| AP1 | 148 | 173 | 46 | 169 |  |  |
| AP1 | 148 | 173 | 46 | 162 |  |  |
| AP1 | 148 | 173 | 46 | 163 |  |  |
| AP1 | 148 | 173 | 46 | 167 |  |  |
| AP1 | 148 | 173 | 46 | 157 |  |  |
| AP1 | 148 | 173 | 74 | 159 |  |  |
| AP1 | 148 | 173 | 73 | 148 |  |  |
| AP1 | 148 | 173 | 73 | 168 |  |  |
| AP1 | 148 | 173 | 37 | 168 |  |  |
| AP1 | 148 | 173 | 62 | 171 |  |  |
| AP1 | 148 | 173 | 62 | 173 |  |  |
| AP1 | 148 | 173 | 36 | 168 |  |  |
| SOC1 | 83 | 113 | 68 | 110 |  |  |
| SOC1 | 83 | 113 | 49 | 90 |  |  |
| SOC1 | 83 | 113 | 67 | 110 |  |  |
| SOC1 | 140 | 169 | 67 | 159 |  |  |
| SOC1 | 140 | 169 | 67 | 162 |  |  |
| SOC1 | 140 | 169 | 68 | 159 |  |  |
| SEP1 | 92 | 114 | 58 | 108 | * |  |
| SEP1 | 92 | 114 | 66 | 108 |  |  |
| SEP1 | 92 | 114 | 58 | 95 |  |  |
| SEP1 | 92 | 114 | 58 | 94 | * |  |
| SEP1 | 92 | 114 | 66 | 105 |  |  |
| SEP1 | 92 | 114 | 66 | 100 |  |  |
| SEP1 | 92 | 114 | 66 | 102 |  |  |
| SEP1 | 92 | 114 | 66 | 97 |  |  |
| SEP1 | 92 | 114 | 66 | 96 |  |  |
| SEP1 | 92 | 114 | 66 | 98 |  |  |
| SEP1 | 92 | 114 | 66 | 93 |  |  |
| SEP1 | 119 | 139 | 66 | 134 |  |  |
| SEP1 | 119 | 139 | 66 | 135 |  |  |
| SEP1 | 119 | 139 | 66 | 132 |  |  |
| SEP1 | 119 | 139 | 66 | 123 |  |  |
| SEP1 | 119 | 139 | 58 | 136 |  |  |
| SEP1 | 119 | 139 | 66 | 130 |  |  |
| FUL | 120 | 137 | 51 | 129 |  |  |
| FUL | 160 | 173 | 35 | 165 |  |  |
| FUL | 160 | 173 | 36 | 165 |  |  |
| FUL | 160 | 173 | 51 | 163 |  |  |
| SEP3 | 94 | 114 | 68 | 111 |  |  |
| SEP3 | 94 | 114 | 58 | 103 |  |  |
| SEP3 | 94 | 114 | 58 | 101 |  |  |
| SEP3 | 94 | 114 | 58 | 107 |  |  |
| SEP3 | 94 | 114 | 50 | 98 |  |  |
| SEP3 | 94 | 114 | 50 | 99 |  |  |
| SEP3 | 94 | 114 | 50 | 101 |  |  |
| SEP3 | 94 | 114 | 50 | 103 |  |  |
| SEP3 | 94 | 114 | 50 | 107 |  |  |
| SEP3 | 94 | 114 | 62 | 97 |  |  |
| SEP3 | 94 | 114 | 55 | 108 |  |  |
| SEP3 | 94 | 114 | 62 | 107 |  |  |
| SEP3 | 94 | 114 | 50 | 97 |  |  |
| SEP3 | 94 | 114 | 62 | 103 |  |  |
| SEP3 | 94 | 114 | 62 | 99 |  |  |
| SEP3 | 94 | 114 | 62 | 101 |  |  |
| SEP3 | 94 | 114 | 50 | 113 |  |  |
| SEP3 | 94 | 114 | 55 | 99 |  |  |
| SEP3 | 94 | 114 | 36 | 99 |  |  |
| SEP3 | 94 | 114 | 55 | 97 |  |  |
| SEP3 | 94 | 114 | 41 | 111 |  |  |
| SEP3 | 94 | 114 | 36 | 103 | * |  |
| SEP3 | 94 | 114 | 50 | 108 |  |  |
| SEP3 | 94 | 114 | 58 | 99 |  |  |
| SEP3 | 94 | 114 | 58 | 108 |  |  |
| SEP3 | 94 | 114 | 65 | 101 |  |  |
| SEP3 | 94 | 114 | 55 | 107 |  |  |
| SEP3 | 94 | 114 | 55 | 103 |  |  |
| SEP3 | 94 | 114 | 65 | 97 |  |  |
| SEP3 | 94 | 114 | 62 | 108 |  |  |
| SEP3 | 94 | 114 | 65 | 103 |  |  |
| SEP3 | 94 | 114 | 58 | 97 | * |  |
| SEP3 | 94 | 114 | 55 | 101 |  |  |
| SEP3 | 94 | 114 | 41 | 108 |  |  |
| SEP3 | 94 | 114 | 68 | 101 |  |  |
| SEP3 | 94 | 114 | 68 | 99 | * |  |
| SEP3 | 94 | 114 | 68 | 98 |  |  |
| SEP3 | 94 | 114 | 68 | 103 | * |  |
| SEP3 | 94 | 114 | 68 | 97 |  |  |
| SEP3 | 94 | 114 | 50 | 111 |  |  |
| SEP3 | 94 | 114 | 58 | 111 | * |  |
| SEP3 | 94 | 114 | 68 | 107 |  |  |
| SEP3 | 94 | 114 | 34 | 103 |  |  |
| SEP3 | 94 | 114 | 34 | 101 |  |  |
| SEP3 | 94 | 114 | 55 | 111 |  |  |
| SEP3 | 94 | 114 | 68 | 108 |  |  |
| SEP3 | 94 | 114 | 65 | 99 |  |  |
| SEP3 | 94 | 114 | 41 | 97 |  |  |
| SEP3 | 94 | 114 | 41 | 107 |  |  |
| SEP3 | 94 | 114 | 41 | 103 |  |  |
| SEP3 | 94 | 114 | 41 | 99 |  |  |
| SEP3 | 94 | 114 | 41 | 101 |  |  |
| SVP | 118 | 134 | 15 | 120 |  |  |
| PI | 79 | 110 | 45 | 88 |  |  |
| PI | 79 | 110 | 46 | 88 |  |  |

**^a^** Correlated mutations for which one of the two positions falls inside a predicted K-domain helix and the other in the MADS or I domain.

**^b^** Star indicates that this correlated mutation is conserved in at least one other MADS protein.
