# Supplementary material for: Conserved and variable correlated mutations in the plant MADS protein network
Source: BMC Genomics. 2010 Oct 28;11:607. doi: 10.1186/1471-2164-11-607 (PMC3017862; doi:10.1186/1471-2164-11-607)
Supplement: Additional file 11 — Correlated mutations based analysis of intermolecular MADS domain protein helix - helix interactions. This file contains an analysis of intermolecular contacts predicted between K-domain helices. [file 1471-2164-11-607-S11.DOC]

**Additional File 11. Correlated mutations based analysis of intermolecular MADS domain protein helix – helix interactions.^a^**

|  |  |  | **All** ^e^ | | **Minimum 5** ^e^ | |
| --- | --- | --- | --- | --- | --- | --- |
| **Helix1** | **Helix2** | **C_possible_** ^b^ | **C_obs_** ^c^ | **F_obs_** ^d^ | **C_obs_** ^c^ | **F_obs_** ^d^ |
| 1 | 1 | 34 | 10 | 0.29 | 7 | 0.21 |
| 1 | 2 | 62 | 28 | 0.45 | 9 | 0.15 |
| 1 | 3 | 33 | 12 | 0.36 | 5 | 0.15 |
| 2 | 2 | 28 | 11 | 0.39 | 6 | 0.21 |
| 2 | 3 | 30 | 11 | 0.37 | 3 | 0.10 |
| 3 | 3 | 8 | 3 | 0.38 | 1 | 0.13 |

^a^ The total number of possible intermolecular helix-helix contacts and the number of intermolecular helix-helix contacts observed in correlated mutation position pairs were compared. The goal of this analysis was to investigate if there is a preference for specific K-domain helices to interact with each other, or that the various helices are equally likely to contact each other.

^b^ C_possible_, maximum number of possible helix – helix contacts. These were obtained by counting the pairs of interacting proteins containing the respective helices. For example, if a given interacting pair of proteins both contain two helices, there would be one possible contact between helix 1 and helix 1, one possible contact between helix 2 and helix 2, and two possible contacts between helix 1 and helix 2.

^c^ C_obs_, observed number of helix – helix contacts. These were obtained by counting helix contacts as predicted by correlated mutations.

^d^ F_obs_, fraction C_obs_/C_possible_.

^e^ All, to calculate C_obs_, all helix contacts predicted by correlated mutation pairs were taken into account; minimum 5, to calculate C_obs_, only helix contacts supported by at least 5 correlated mutation pairs were taken into account.
